# An Extremely Peramorphic Newt (Urodela: Salamandridae: Pleurodelini) from the Latest Oligocene of Germany, and a New Phylogenetic Analysis of Extant and Extinct Salamandrids

**DOI:** 10.1371/journal.pone.0137068

**Published:** 2015-09-30

**Authors:** David Marjanović, Florian Witzmann

**Affiliations:** 1 Museum für Naturkunde, Leibniz Institute for Evolutionary and Biodiversity Research, Humboldt University, Berlin, Germany; 2 Department of Ecology and Evolutionary Biology, Brown University, Providence, Rhode Island, United States of America; New York Institute of Technology College of Osteopathic Medicine, UNITED STATES

## Abstract

We describe an Oligocene newt specimen from western Germany that has gone practically unnoticed in the literature despite having been housed in the Museum für Naturkunde (Berlin) for a century. It is referable to the coeval *Chelotriton*, but is unusually peramorphic; for many characters it is more peramorphic than all other caudates or even all other lissamphibians. Most noticeable are the position of the jaw joints far caudal to the occiput, the honeycombed sculpture on the maxilla, and the possible presence of a septomaxilla (which would be unique among salamandrids). Referral to a species would require a revision of the genus, but the specimen likely does not belong to the type species. A phylogenetic analysis of nonmolecular characters of Salamandridae, far larger than all predecessors, confirms the referral to *Chelotriton*. It further loosely associates the Oligocene *Archaeotriton* and the Miocene *Carpathotriton* with the extant *Lissotriton*, though the former may alternatively lie outside Pleurodelinae altogether. The Miocene? *I*. *randeckensis* may not belong to the extant *Ichthyosaura*. The Miocene *“Triturus” roehrsi* is found neither with the extant *Ommatotriton* nor with *Lissotriton*, but inside an Asian/aquatic clade or, when geographic distribution is included as a character, as the sister-group to all other European molgins. The main cause for discrepancies between the results and the molecular consensus is not heterochrony, but adaptations to a life in mountain streams; this is the most likely reason why the Paleocene *Koalliella* from western Europe forms the sister-group to some or all of the most aquatic extant newts in different analyses. We would like to urge neontologists working on salamandrids to pay renewed attention to the skeleton, not limited to the skull, as a source of diagnostic and phylogenetically informative characters.

## Introduction

**Fig 1:**
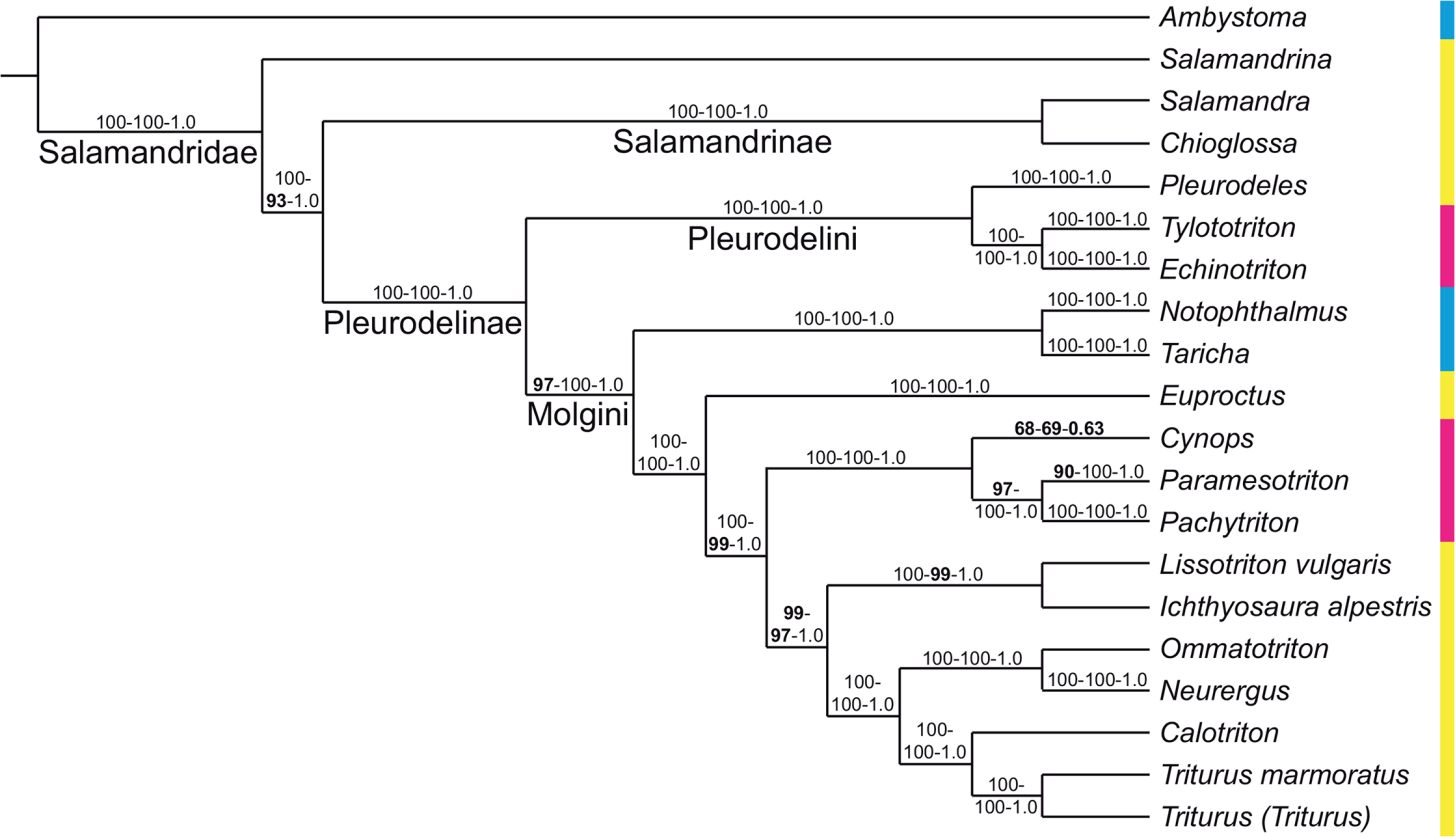
Phylogenetic tree of Salamandridae from Fig 2 of [1], reduced to the taxa it shares with our analysis; branch lengths not shown, names of larger clades added. The dataset consists of mitochondrial genomes. Weighted parsimony, partitioned maximum likelihood and partitioned Bayesian inference all produced the same topology. The numbers at each internode represent parsimony bootstrap percentages, partitioned likelihood bootstrap percentages and partitioned Bayesian posterior probabilities from left to right; values below 100% are in boldface. The colors of the sidebar represent geographic distribution: cyan = North America; yellow = Europe, Mediterranean, Iran; magenta = eastern Asia.

**Fig 2:**
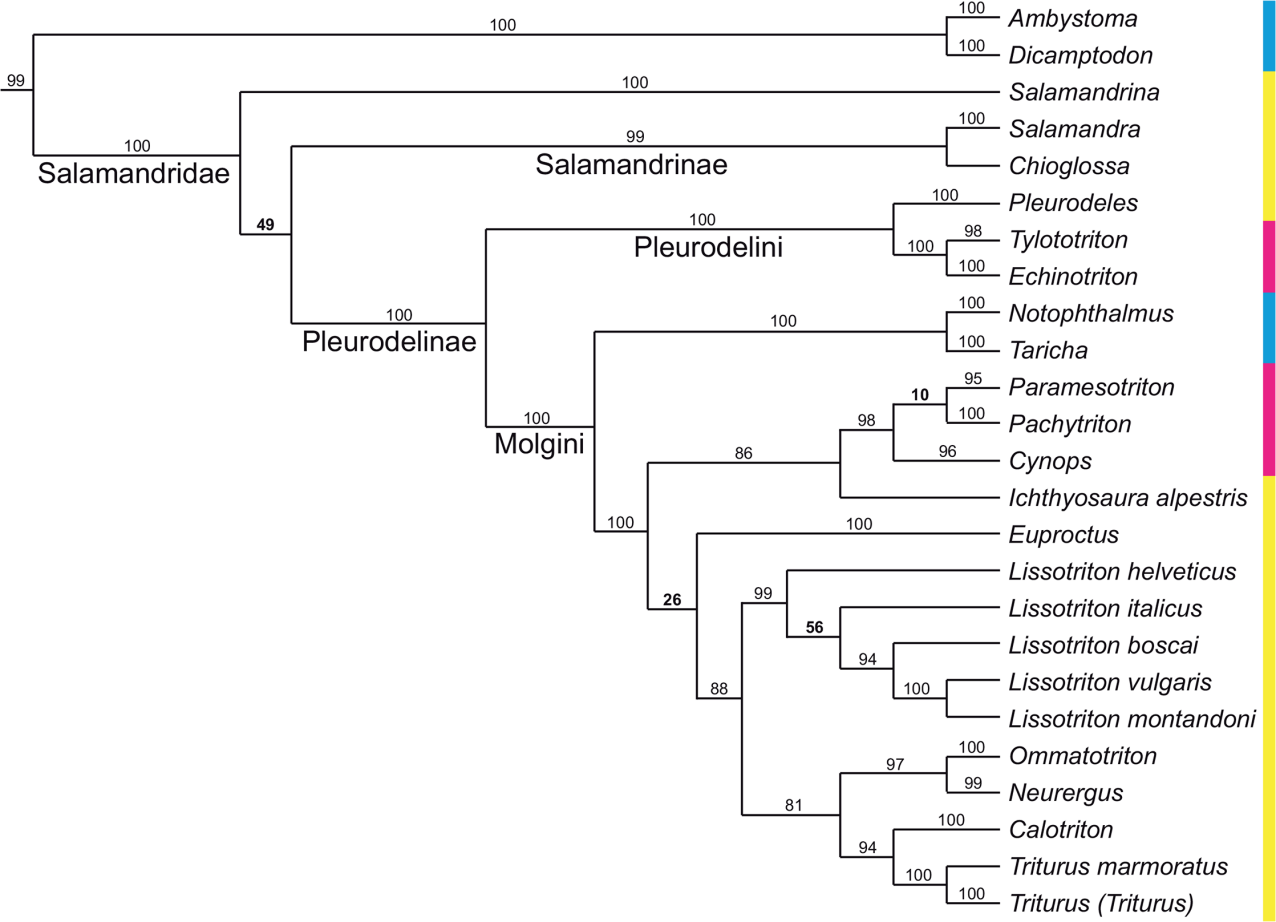
Phylogenetic tree of Salamandridae from the supplementary file amph_shl.tre of [2], reduced to the taxa it shares with our analysis; branch lengths not shown, names of larger clades added. The dataset consists of nine nuclear and three mitochondrial genes and was analysed by maximum likelihood. The numbers at each internode represent percentages of the nonparametric Shimodaira/Hasegawa-like implementation of the approximate likelihood-ratio test; values below 80% are in boldface. Colors as in Fig 1.

**Fig 3:**
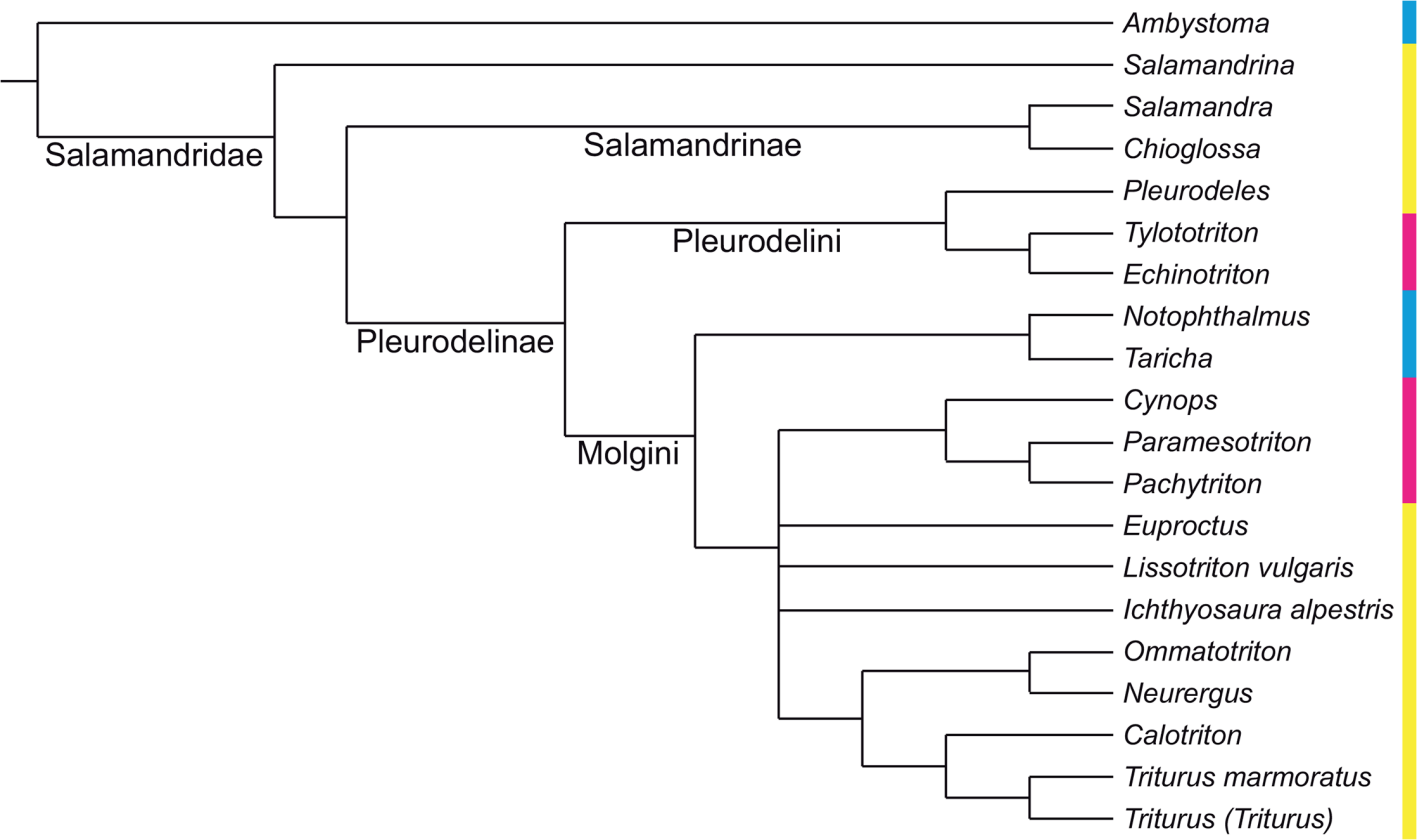
Strict consensus of Figs 1 and 2, reduced to the taxa they both share. This tree was used as a backbone constraint in our constrained analysis. Note the absence of *Dicamptodon* and of all species of *Lissotriton* except one, following [1]. Colors as in Fig 1.

Salamandridae is an extant clade of caudates that is widely distributed in the northern hemisphere and can be found in Asia, Europe and North America, as well as the northern fringe of Africa [1]. The group is traditionally divided into the “true salamanders” (Salamandrinae), which are mostly terrestrial, and the generally more aquatic newts (Pleurodelinae); the terrestrial *Salamandrina* forms a third clade [1–3] (Figs 1–3) that is sometimes called Salamandrininae and appears to form the sister-group to the clade formed by Salamandrinae and Pleurodelinae together ([1]; weakly supported by [2]). The oldest known fossil salamandrid is *Koalliella genzeli* Herre, 1950, a likely crown- or stem-pleurodeline known from a few isolated vertebrae from the late Paleocene of Germany and France ([3–7]; all specimens lost according to [4]). The lack of older fossils, or of any stem-salamandrids, has made it difficult to estimate the time of origin of the salamandrid crown. The molecular divergence dating analysis in [1] put the 95% confidence interval at approximately 73 to 86 Ma ago when the program MultiDivTime was used and at approximately 58 to 83 Ma ago when BEAST was used instead, while the very simple method used in appendix S5 of [2] on its enormous dataset yielded an age of 75.26 Ma without error margins; the paleontological method of [7] yielded broadly compatible but poorly constrained ages (68.2 Ma according to fig 2 of [7]). In the analyses cited above, Pleurodelinae was found to consist of a clade comprising (*Pleurodeles* + (*Tylototriton* + *Echinotriton*)), referred to as Pleurodelini or as “primitive newts” [1], and its sister-group which contains the remaining newts (Molgini, after *Molge*, a junior synonym of *Triturus*). Pleurodelini is characterized by an overall high degree of ossification, strongly sculptured skulls with a complete frontosquamosal arch (or bar or bridge) separating the orbitotemporal fenestra from the postorbital opening, and tubercular processes on the ribs located internal to toxic skin glands; *Tylototriton* and *Echinotriton* additionally possess sculptured spine tables on the tips of their neural spines [4,5,8,9], a trait that also occurs (in less extreme form) in certain molgins. There is a number of Eocene to Pliocene salamandrids from Europe that show a similar morphology and degree of ossification of the skeleton and that are often thought to belong to Pleurodelini. These comprise fossil representatives of *Pleurodeles* sp. from the Miocene/Pliocene of the Iberian Peninsula and North Africa [4], *Palaeopleurodeles hauffi* Herre, 1941, from the Oligocene of Germany [10,11], *Brachycormus noachicus* (Goldfuss, 1831) from the late Oligocene of Germany [3,4,12–15] and the Eocene to Pliocene genus *Chelotriton*. Extant pleurodelins and the abovementioned fossil forms have been referred to as “Group II” salamandrids [4,16] and as the “*Pleurodeles*-*Tylototriton* Clade” [5], although a phylogenetic analysis incorporating all these forms has so far not been carried out.

The diversity of material from which *Chelotriton* is known has been referred to a minimum of three species: *C*. *robustus* Westphal, 1980, which is restricted to the middle Eocene deposits of Messel, Germany [17]; *C*. *pliocenicus* Bailon, 1989, which has been found in an upper Pliocene site in France [18] and possibly a lower to middle Miocene site in Germany [19]; and the type species, *C*. *paradoxus* Pomel, 1853, to which material from lower Oligocene to upper Miocene sites of France, Germany, Austria, Romania and Spain has been referred [4,20–30]. Recently [31,32], two additional types of isolated *Chelotriton* skull bones were reported (“*Chelotriton* sp., type I” and “*Chelotriton* sp., type II”), both of which occur in the early Miocene site of Mokrá in the Czech Republic and in the late Miocene site of Rudabánya in Hungary; the atlantes referred to “type I” in [32] further differ from both those from the early Miocene Czech site of Merkur North and those from the middle Miocene French site of Sansan, while the other vertebrae referred to the same type are identical to those from Sansan ([32]: 474) even though they differ from the single (postatlantal) vertebra referred to “type II” ([32]: 475). Clearly, the genus needs to be revised [19] to determine how many species are represented by the known material (as opposed to ontogenetic variation or sexual dimorphism).

*Chelotriton ogygius* (Goldfuss, 1831) from the late Oligocene of Germany and possibly the early Miocene of Spain further adds to this confusion: the immature and poorly preserved type specimen [21] “seems to be lost” ([4]: 76), and based on the published descriptions (most detailed: [21]) it may not be possible to distinguish *C*. *ogygius* from the material referred to *C*. *paradoxus* except by likely ontogeny-related features. In case of synonymy, the name *C*. *ogygius* would have priority; [4] (page 77) recommended that “the relatively unknown name *ogygius* should probably be suppressed by the ICZN” if it should turn out to be a senior synonym of *C*. *paradoxus*, but no such petition has so far been submitted to the International Commission on Zoological Nomenclature, making it all the more important to determine whether the type specimen is lost and whether a neotype should be designated. (Reportedly–[27]: 175; our translation–“[t]he type material is situated in Berlin”, but we have not been able to find it in the collection of the Museum für Naturkunde. Miklas [27] did not claim to have seen the material, was evidently unaware that *Chelotriton ogygius* and *Salamandra ogygia* Goldfuss, 1831, are objective synonyms, and ascribed the latter name to von Meyer, 1860 [21], who instead created the new genus *Polysemia* for it.) That, however, is beyond the scope of this paper; such work would best be carried out as part of a wholesale revision of *Chelotriton*.

Fossil remains attributed to *Chelotriton* sp. have been reported from Austria [33], England, the Czech Republic, Hungary and Ukraine [5]. The status of the single specimen of *Tylototriton weigelti* Herre, 1935 [34], from the middle Eocene lignite of Geiseltal, Germany, is not clear; neither is that of the isolated vertebrae called “cf. *Tylototriton* sp.” from the lower Oligocene of Möhren, Germany [19]. Whereas [4,8] regarded *T*. *weigelti* as belonging to *Tylototriton*, [5,15] more recently suggested that it probably belongs to *Chelotriton* without commenting on its species-level affinities.

**Fig 4:**
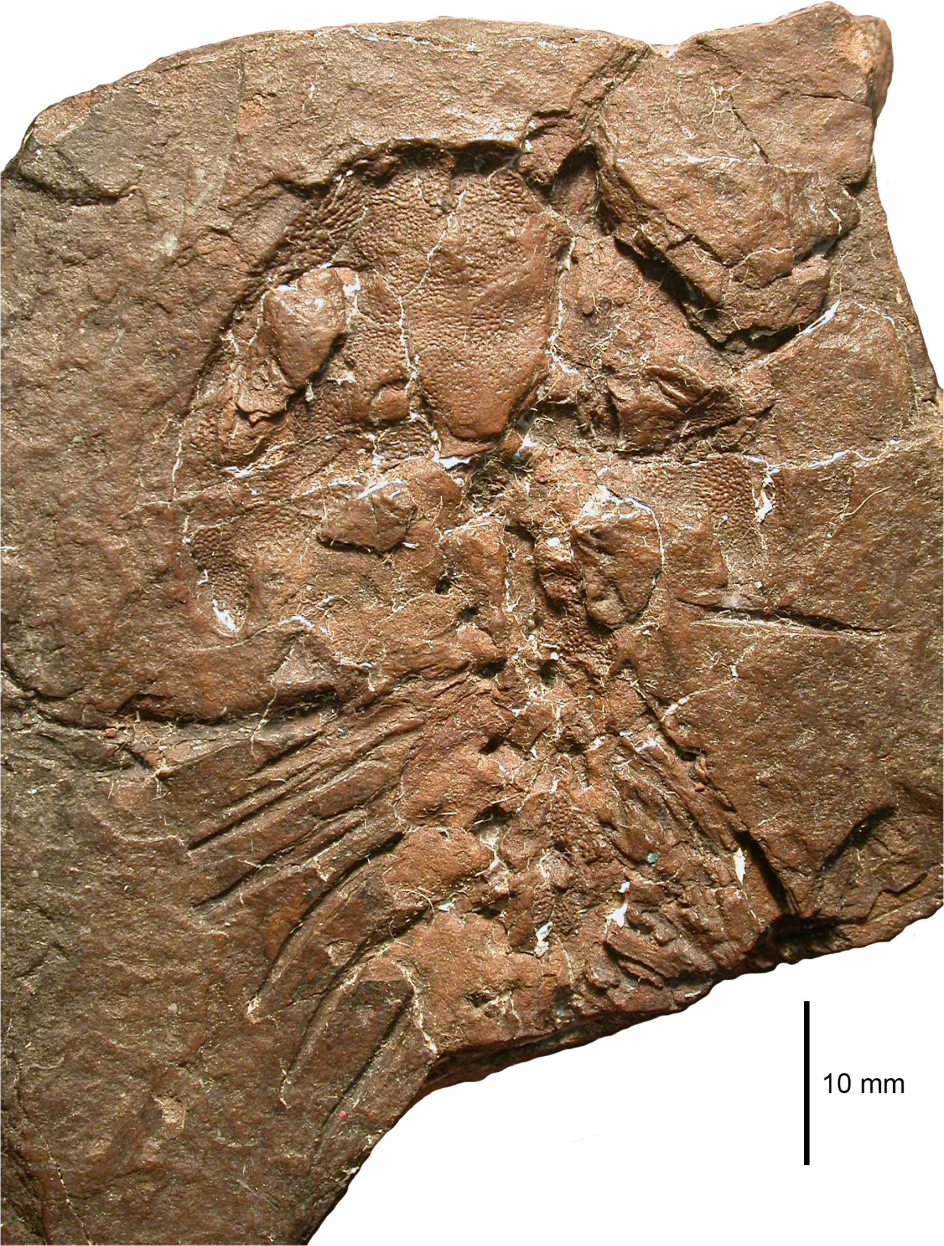
Photograph of MB.Am.45.1.

A salamandrid specimen from the late Oligocene lignite of Orsberg near Erpel, Germany (the type locality of *Chelotriton ogygius*), is stored in the collection of fossil amphibians in the Museum für Naturkunde, Berlin, under the inventory number MB.Am.45 (Fig 4 and S1 Fig). It exhibits peramorphosis to a degree unknown in any other caudate, in the case of a few characters even unknown in any other lissamphibian. The specimen superficially resembles Paleozoic tetrapods such as amphibamid temnospondyls or diplocaulid lepospondyls to such a degree that it was determined as a “dissorophid temnospondyl,? Amphibamus” on a label dated 1981 (the name Amphibamidae Moodie, 1910, was considered a synonym of Dissorophidae at the time). According to the most recent labels, it was later regarded as “*Tylototriton kosswigi* Herre, 1949”, a junior synonym of *Brachycormus noachicus* [4].

The oldest surviving label mentions that the name of the locality was “written in the box by O. Jaekel” (our translation), so MB.Am.45 has been known to science for a century. (The original box is not preserved.) Yet, we have been unable to find a mention of it in the literature, with one exception: the specimen was mentioned and referred to *Chelotriton paradoxus*, but neither described nor illustrated, on page 324 of [29].

### Phylogenetic nomenclature

Our use of the names Caudata and Urodela follows current widespread usage: Urodela is the crown-group of salamanders (the last common ancestor of all extant salamanders, plus all its descendants), while Caudata is the corresponding total group, containing Urodela and everything closer to it than to frogs (crown: Anura) or caecilians (crown: Gymnophiona).

### Aims

We here describe and illustrate MB.Am.45 for the first time, referring it to *Chelotriton* and comparing it to the species and possible species of that taxon; unfortunately, nomenclature has to be left open at least as long as *Chelotriton* is not revised. We discuss the often unusual features that can be attributed to heterochrony. We further present the largest data matrix of nonmolecular characters of salamandrids yet published; four phylogenetic analyses—two unconstrained, two constrained by the current consensus derived from molecular data; two without and two with an additional character that represents geographic distribution—provide phylogenetic context for MB.Am.45 with respect to *Chelotriton*, *Brachycormus* and other extinct salamandrids, as well as *Tylototriton*, *Echinotriton* and other extant salamandrids.

## Material and Methods

No permits were required for the described study, which complied with all relevant regulations.

### Systematic paleontology

Salamandridae Goldfuss, 1820, as Salamandrae; first used in its present form by Gray, 1825

Pleurodelinae Tschudi, 1838, as Pleurodelae; first used in its present form by Risch, 1985

Pleurodelini Tschudi, 1838, as Pleurodelae; first used in its present form by Massalongo, 1853

Genus *Chelotriton* Pomel, 1853 [20]

*Heliarchon* von Meyer, 1860a

*Polysemia* von Meyer, 1860b [21] (preoccupied by *Polysemia* Guenée in Boisduval & Guenée, 1857)

*Grippiella* Herre, 1949 [23]

*Palaeosalamandrina* Herre, 1949 [23]

*Tischleriella* Herre, 1949 [23]

*Epipolysemia* Brame, 1973 (replacement name for *Polysemia* von Meyer, 1860)

#### Type species

*Chelotriton paradoxus* Pomel, 1853 [20]

#### Diagnosis

“A common Middle [sic] Eocene-Upper [sic] Miocene salamandrid from Europe, closely related to living *Tylototriton* [including *Echinotriton* at the time], and differing principally from the latter in having pustular rather than pit and ridge sculpture on dermal bone, reaching a somewhat larger size (adult snout-vent length about 100–140 mm), lacking ossification of carpals and tarsals except in largest individuals and by having the fourteenth or fifteenth vertebra as the sacrum, rather than the sixteenth or seventeenth in *Tylototriton*.

These differences are small, but appear consistent; the slightly longer vertebral column and different head width/length ratio in *Tylototriton* place the latter on a different growth line in graphical representation of skeletal ratios. *Tylototriton* is about equally distant from *Brachycormus* in growth line, suggesting that three genera are involved.” ([4]: 72)

By alluding to a “different head width/length ratio”, [4] must have meant the fact that *Chelotriton* (including *C*. *ogygius*: [21]: 59, 66) has an unusually wide skull that is broad-parabolic in dorsal or ventral view, as pointed out in [30]; this appears to be an autapomorphy and manifests also as an unusually high ratio of interorbital width to skull length.

The bluntly pentagonal shape of the nasals which are about as wide as long [30] appears to be a further autapomorphy.

A third autapomorphy is suggested by our phylogenetic analysis: unusually long rib-bearers (see Results and Discussion: Salamandrid phylogeny or the Appendix, character state 68(2), for quantification) are found in vertebrae referred to *C*. *paradoxus* from a broad range of localities ([4]: figs 18C–F; [26]: fig 6d; [27]: plate 2M; [35]: plate X figs 7 and 8), in *C*. *pliocenicus* [18], borderline in *C*. *robustus* ([17]: Fig 4a), in vertebrae referred to *C*. sp. [33], in *Chelotriton* specimens from Enspel [30] and in MB.Am.45. Other *Chelotriton* vertebrae have not been figured in dorsal view to our knowledge.

*Chelotriton* often occurs together with the neotenic pleurodeline *Brachycormus* von Meyer, 1860 [21], the type and only known species of which—*B*. *noachicus*—is distinguishable from *Chelotriton* by at least one clearly ontogeny-independent feature: its sacral vertebra is most likely the thirteenth [14]. A more recent source [30] confirms that the sacral vertebra is consistently the fourteenth in *Chelotriton* specimens from Enspel and the crater lake (Maar) site of Randeck. It should be noted here, however, that [21] estimated only 12 presacral vertebrae for *C*. *ogygius*; the first ten were preserved, as were the presumed sacral and proximal caudal vertebrae, and the gap, illustrated in plate VIII fig 1 of [21], would not have accommodated three vertebrae in von Meyer’s [21] opinion.

Additionally, [18] (on pages 10–11; translated by DM) remarked that the holotype of *Chelotriton pliocenicus* (a dorsal vertebra, like the neotype of *C*. *paradoxus*) can be referred to *Chelotriton* and distinguished from *Tylototriton* based on “the size of the vertebra, the tubercular relief of the dermal plate, the connection of the prezygapophysial crest with the costal process and the absence of a deep incision behind the costal process”.

At first glance, *Chelotriton* skeletons often look similar to the type and only known specimen of *Salamandra laticeps* von Meyer, 1858. (The specimen was more recently [4] reported lost and cautiously referred to *S*. *sansaniensis* Lartet, 1851.) In that specimen, however, “there is no trace of frontosquamosal arches although the specimen is well-preserved in dorsal view” ([4]: 69) and although the shapes of the skull and the ribs clearly indicate a morphologically adult, even peramorphic individual ([21]: plate VIII fig 2; reproduced in lower resolution as [4]: fig 16A). Furthermore, “[a]lthough detailed vertebral form cannot be made out there appear to be 15–16 presacral vertebrae as in *S*. *salamandra*” ([4]: 69) rather than 13 as in *Chelotriton*, but we caution that the number of presacral vertebrae varies e.g. from 15 to 17 within the extant *Salamandra infraimmaculata infraimmaculata* in Israel alone [36].—Although [21] estimated only 14 presacrals on page 63, noting the supposed distinction to *S*. *salamandra*, this is difficult to reconcile with plate VIII fig 2 of [21].

*Chelotriton* sp.

MB.Am.45

(Figs 4–14, S1–S3 Figs)

**Fig 5:**
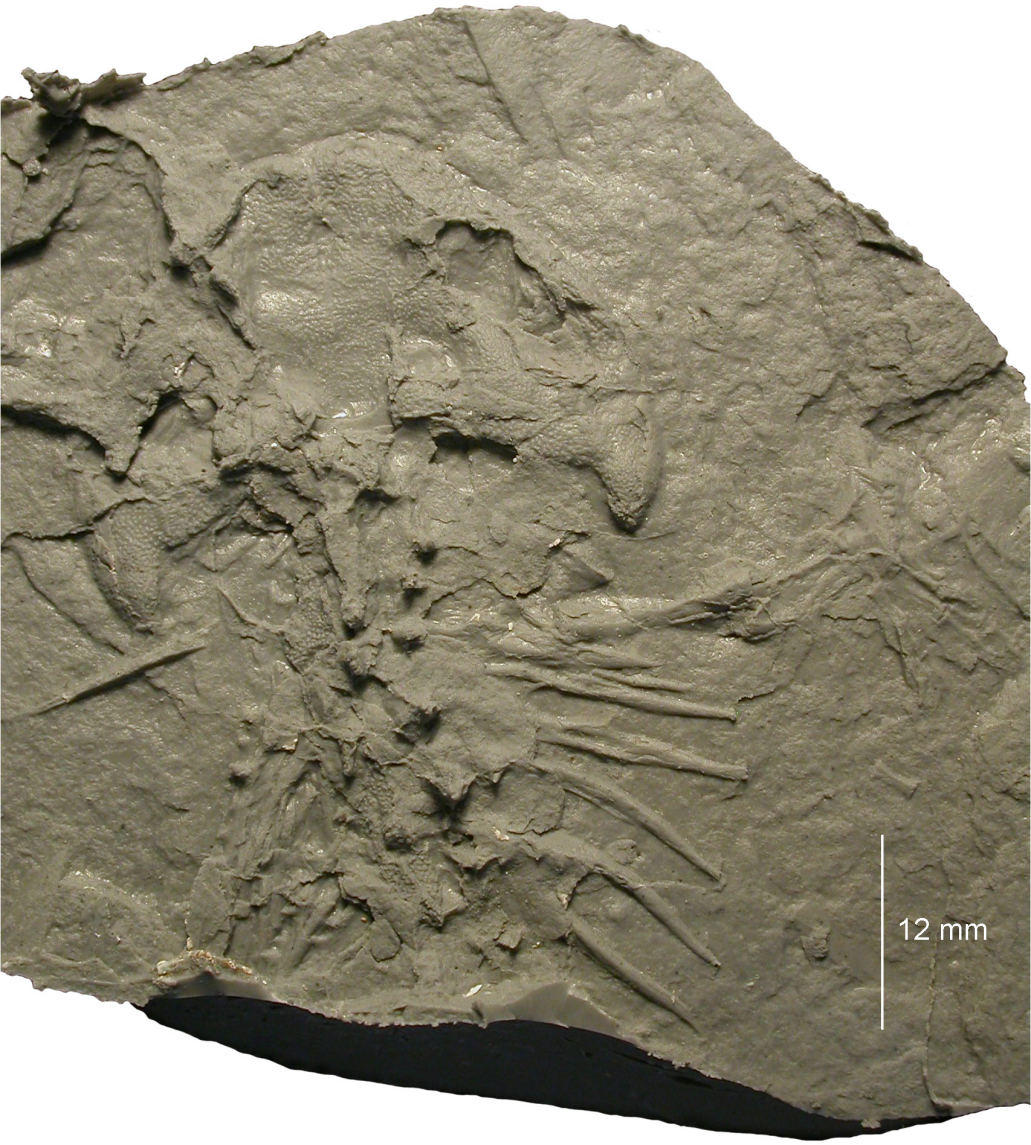
Photograph of the silicone cast MB.Am.45.3.

**Fig 6:**
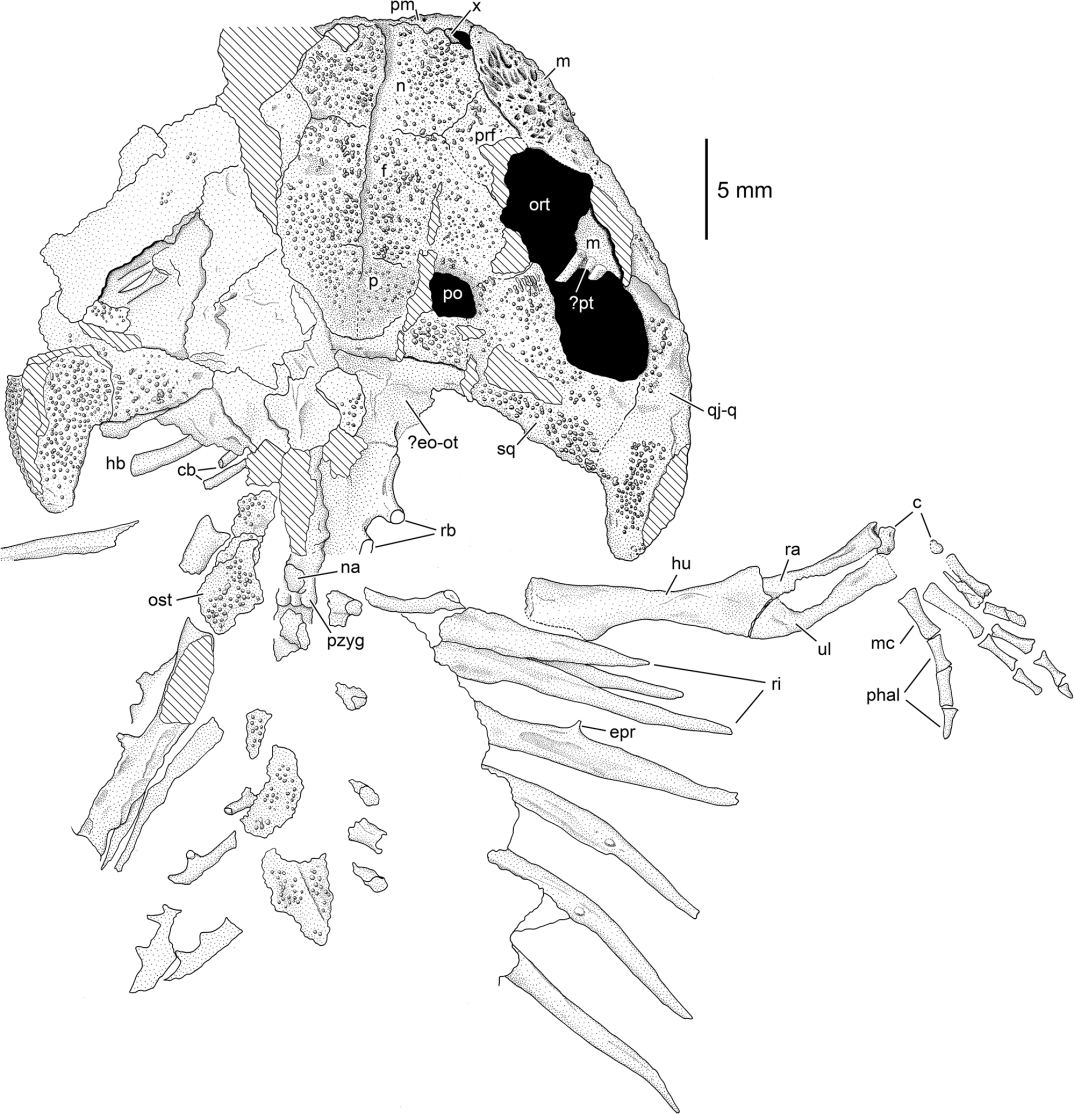
Drawing of MB.Am.45.3. Abbreviations: c, carpals; cb, ceratobranchials;? eo-ot, probably exoccipital and opisthotic (see text); epr, epipleural process; f, frontal; hb, hypobranchial; hu, humerus; m, maxilla; mc, metacarpal; n, nasal; nsp, partial neural spine; ort, orbitotemporal fenestra; spt, spine table; p, parietal; phal, manual phalanges; pm, premaxilla; po, postorbital opening (supratemporal fenestra); prf, prefrontal;? pt, probable pterygoid; pzyg, postzygapophysis; qj-q, quadratojugal-quadrate bone; ra, radius; rb, rib-bearers; ri, rib; sq, squamosal; ul, ulna; x, bone of unclear identity (see text).

**Fig 7:**
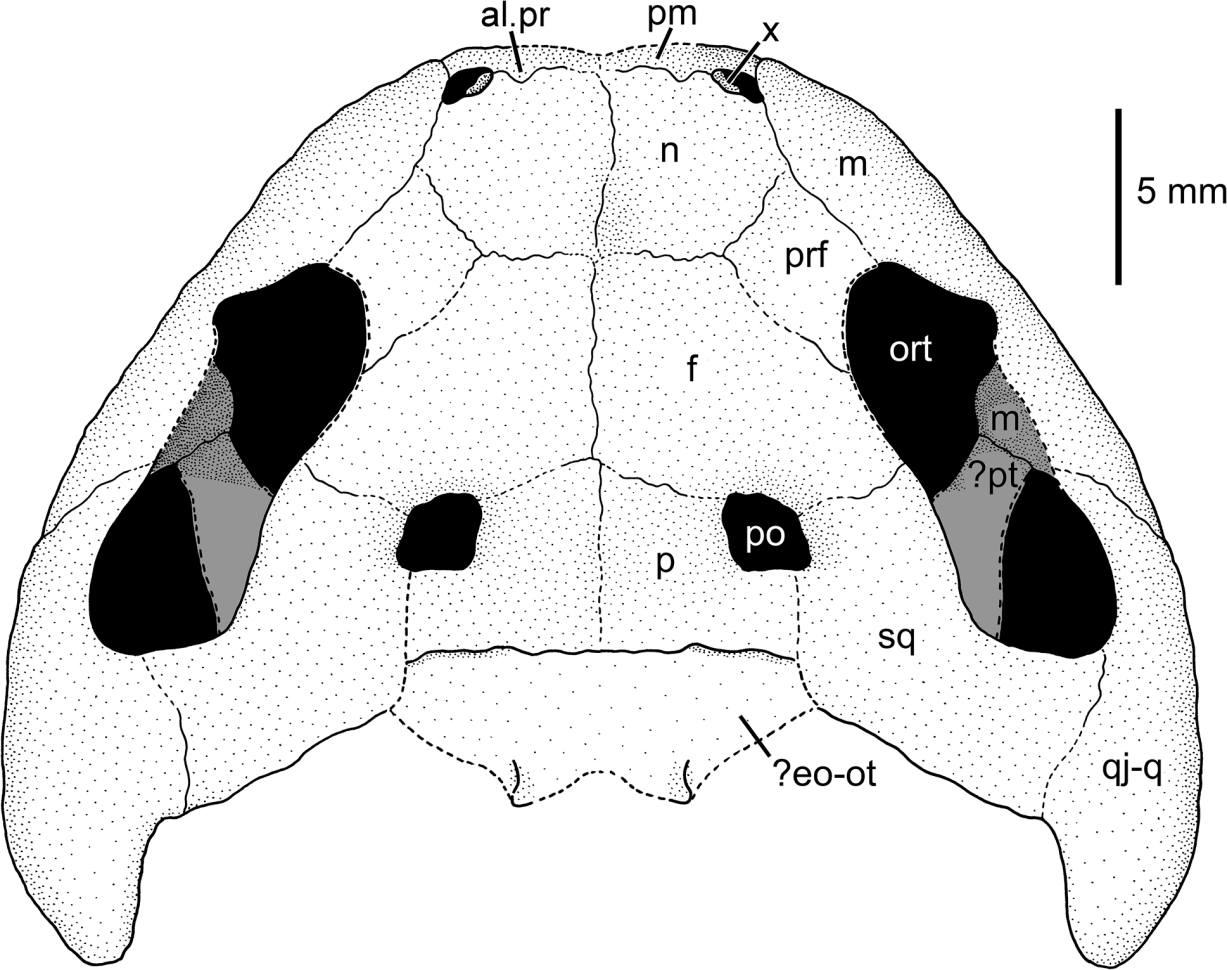
Reconstruction of the skull of MB.Am.45 in dorsal view. Sculpture omitted. We have not corrected for dorsoventral compression because the palate is unknown; note, however, that the skull was clearly rather flat in life, there have not been any anteroposterior shifts, and the dorsoventral compression appears to have slightly decreased the interorbital width. Abbreviations: al.pr, alary process of premaxilla;? eo-ot, probably exoccipital and opisthotic (see text); f, frontal; m, maxilla; n, nasal; ort, orbitotemporal fenestra; p, parietal; pm, premaxilla; po, postorbital opening (supratemporal fenestra); prf, prefrontal;? pt, probable pterygoid; qj-q, quadratojugal-quadrate bone; sq, squamosal; x, bone of unclear identity (see text).

**Fig 8:**
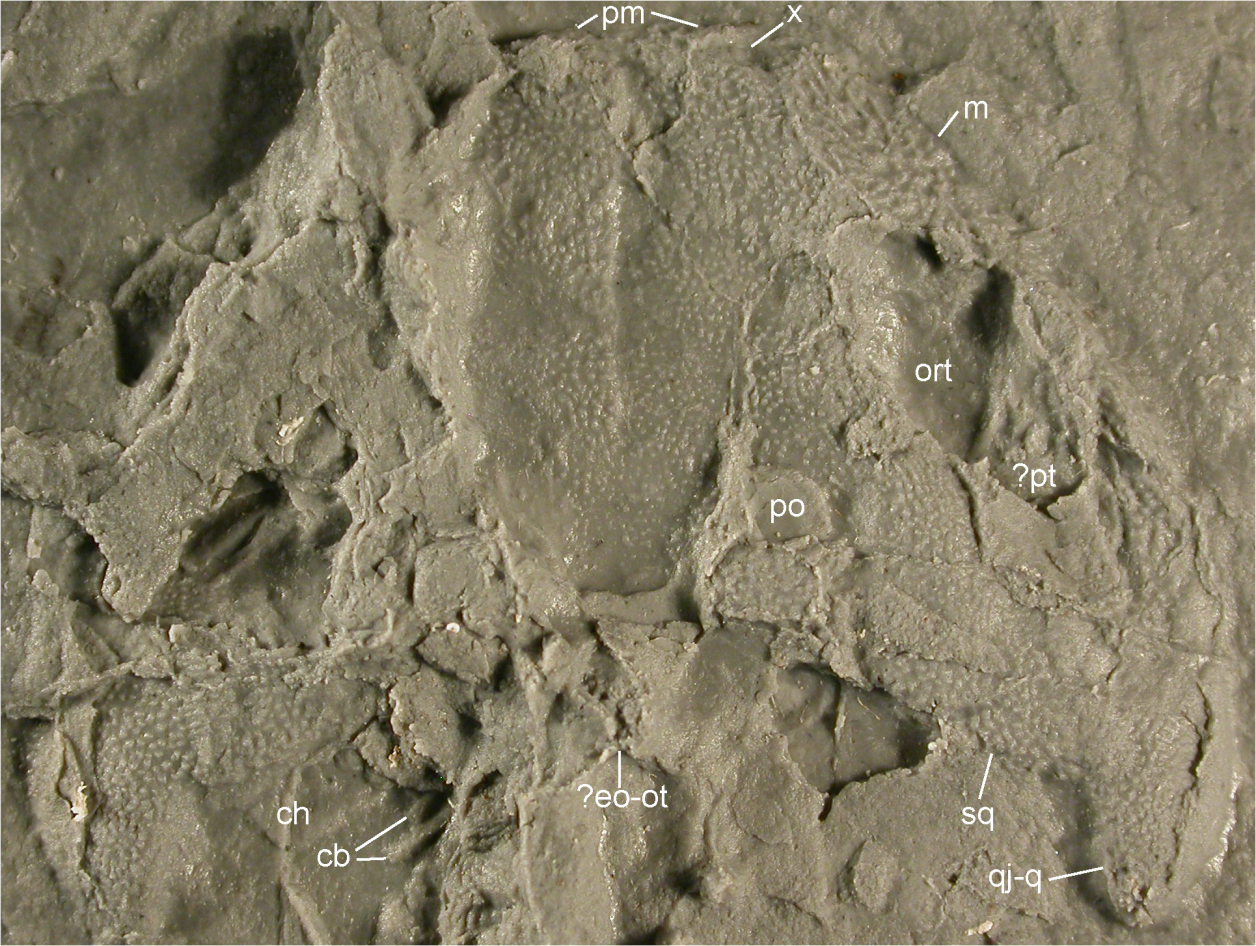
Skull of MB.Am.45 in dorsal view. Photograph of MB.Am.45.3 showing honeycombed sculpture on the right maxilla and tubercles on most other bones (connected to form ridges on the right frontosquamosal arch) as well as the sutures between the parietals, frontals, nasals and prefrontals (compare Fig 7). Additionally, the left unidentified bone is visible, as are the vertical triangular processes of the premaxillae. Abbreviations: cb, ceratobranchials; ch, ceratohyal;? eo-ot, probably exoccipital and opisthotic (see text); m, maxilla; ort, orbitotemporal fenestra; pm, premaxilla; po, postorbital opening (supratemporal fenestra);? pt, probable pterygoid; qj-q, quadratojugal-quadrate bone; sq, squamosal; x, bone of unclear identity (see text). The nasals, prefrontal, frontals and parietals are not marked in order to avoid obscuring their sculpture; compare Fig 7.

**Fig 9:**
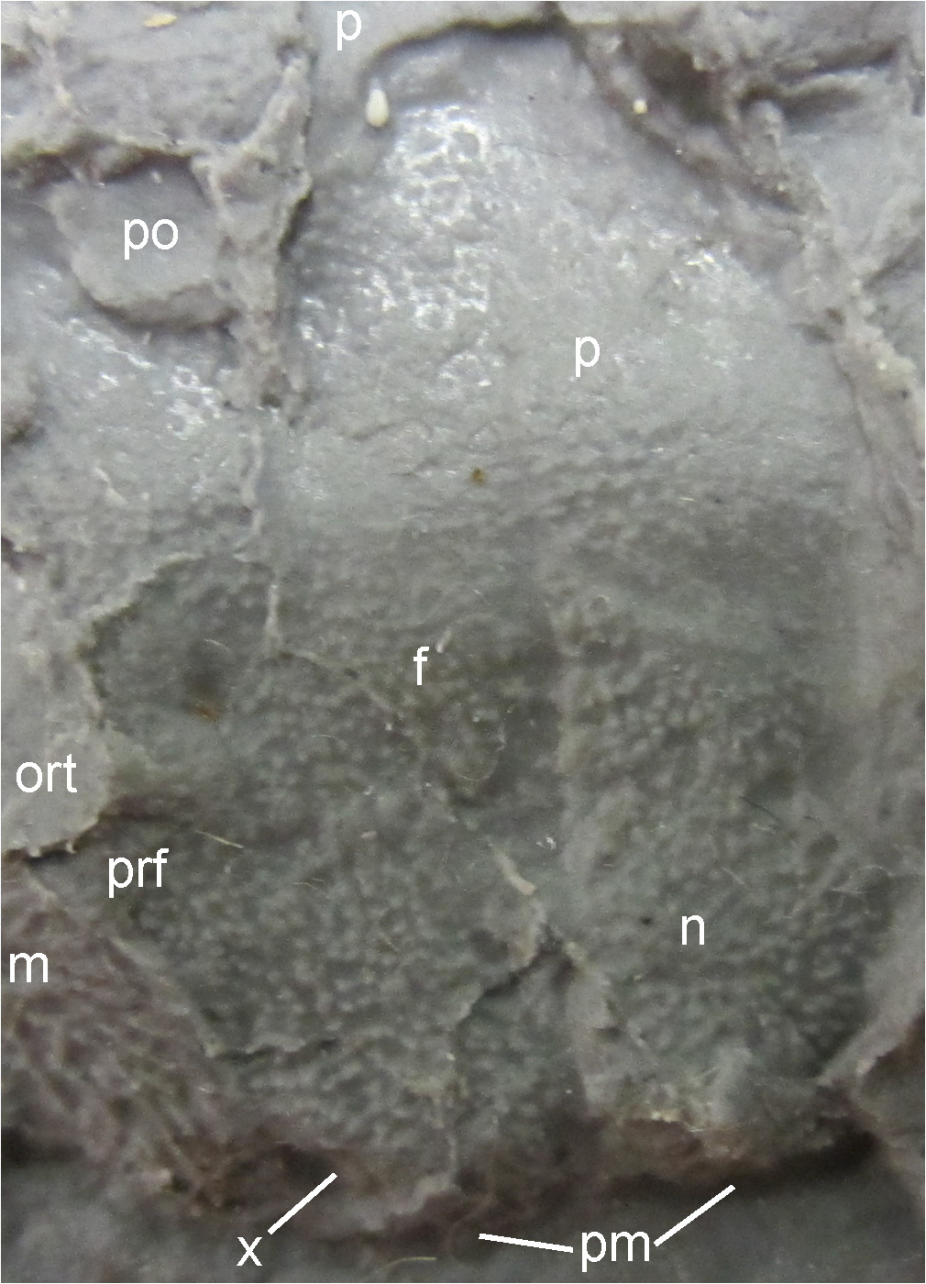
Skull table of MB.Am.45 in anterodorsal view. Photograph of MB.Am.45.3 taken at a low angle under daylight, showing the three-dimensional shape of the skull roof with depressed parietals and a transverse bulge across the frontals; the sutures between the frontals, nasals and prefrontals can also be seen (compare Fig 7). Abbreviations: f, frontal; m, maxilla; n, nasal; ort, orbitotemporal fenestra; p, parietal; pm, premaxilla; prf, prefrontal; po, postorbital opening (supratemporal fenestra); x, bone of unclear identity (see text).

**Fig 10:**
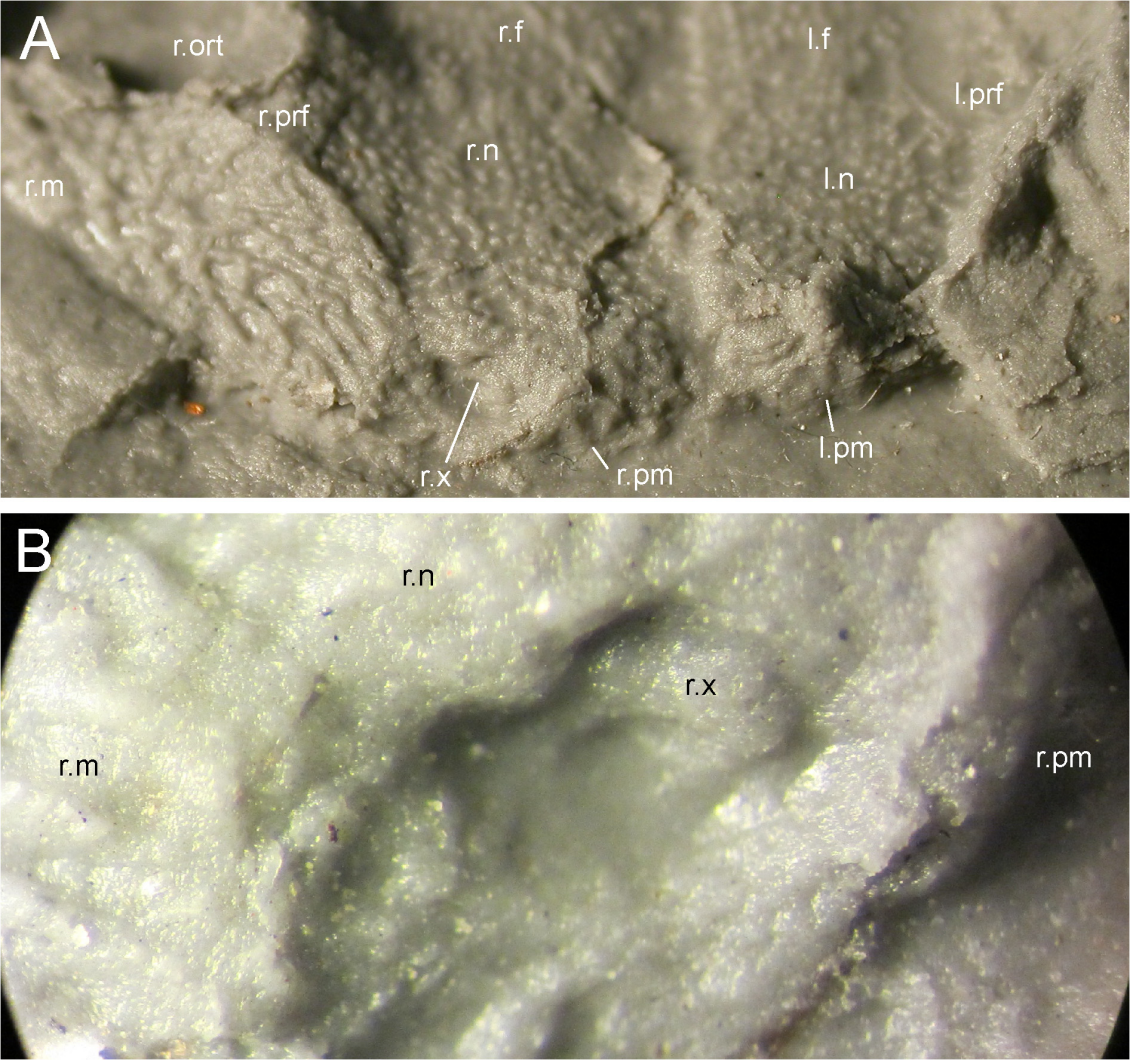
Snout of MB.Am.45 at low angles in anterodorsal view, showing bone of unclear identity (see text). Photographs of MB.Am.45.3, showing premaxillae, right maxilla, right unidentified bone, right nasal and right prefrontal in focus; the sculpture and various sutures are visible. (A) in anterodorsal view; (B) was taken in anterodorsolateral view by holding a camera to an ocular of a binocular microscope and is a close-up on the right nostril (the bottom of which is out of focus) and its surroundings, in particular the right unidentified bone. Abbreviations: l., left; r., right; f, frontal; m, maxilla; n, nasal; ort, orbitotemporal fenestra; pm, premaxilla; prf, prefrontal; x, bone of unclear identity.

**Fig 11:**
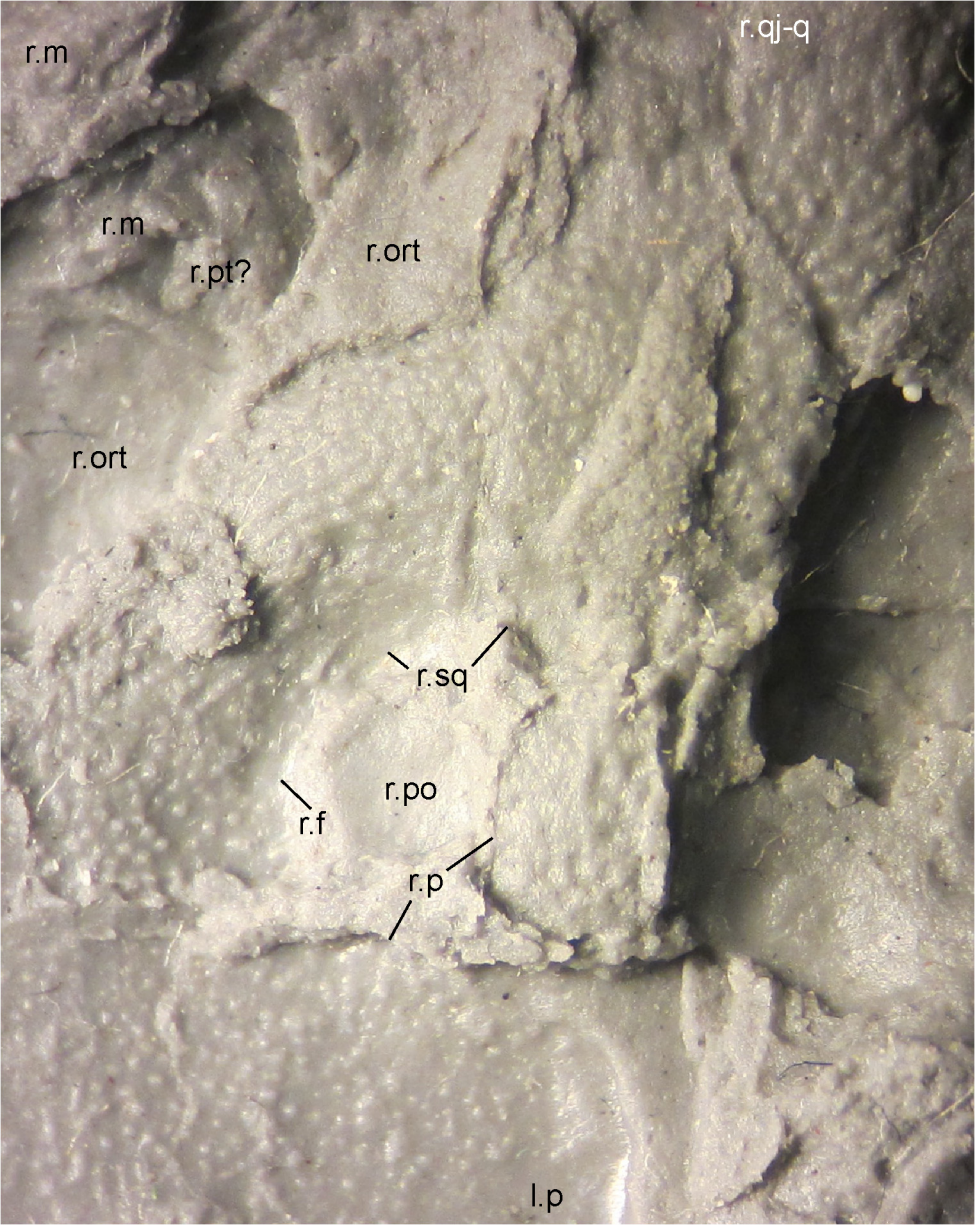
Right postorbital opening (supratemporal fenestra) and surroundings of MB.Am.45. Photograph of MB.Am.45.3 taken by holding a camera to an ocular of a binocular microscope, showing the diversity of sculpture around the postorbital opening. Anterior is to the left. Note how little space remains (covered by a prominent break) for an unsculptured trough on the right parietal that would have connected the posteromedial corner of the postorbital opening to the posterior margin of the skull and would have allowed jaw-closing muscles passing through the fenestra to attach to neural spines. Abbreviations: l., left; r., right; f, frontal; m, maxilla; ort, orbitotemporal fenestra; p, parietal; po, postorbital opening (supratemporal fenestra); pt?, probable pterygoid; qj-q, quadratojugal-quadrate bone; sq, squamosal.

**Fig 12:**
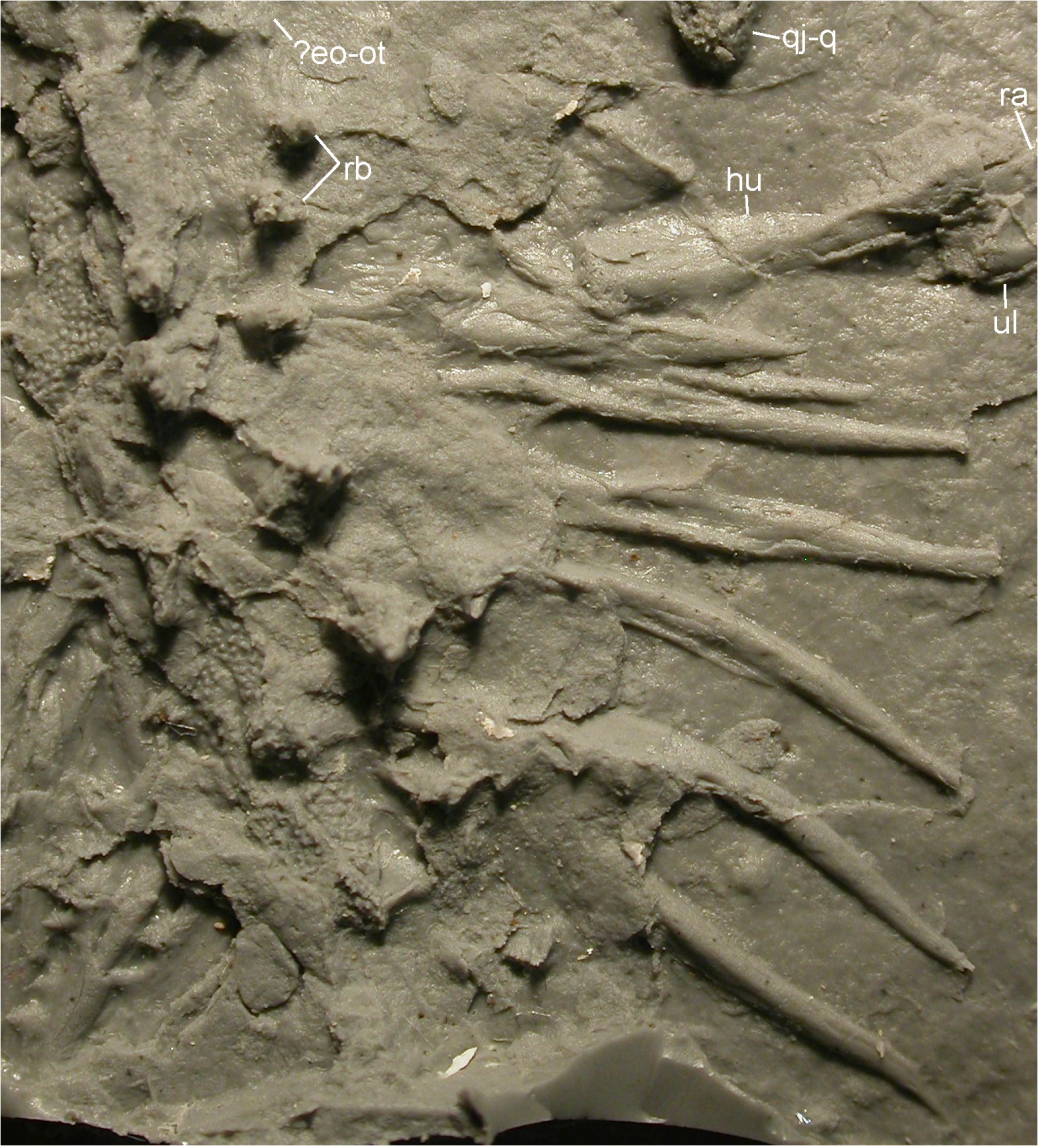
Right side of the trunk of MB.Am.45. Photograph of MB.Am.45.3 showing the vertebrae (with right rib-bearers and sculptured spine tables), the right ribs, the right humerus and elbow, and much of the right posterior rim of the skull. Anterior is to the top left. Abbreviations:? eo-ot, probably exoccipital and opisthotic (see text); hu, humerus; qj-q, quadratojugal-quadrate bone; ra, radius; rb, rib-bearers; ul, ulna.

**Fig 13:**
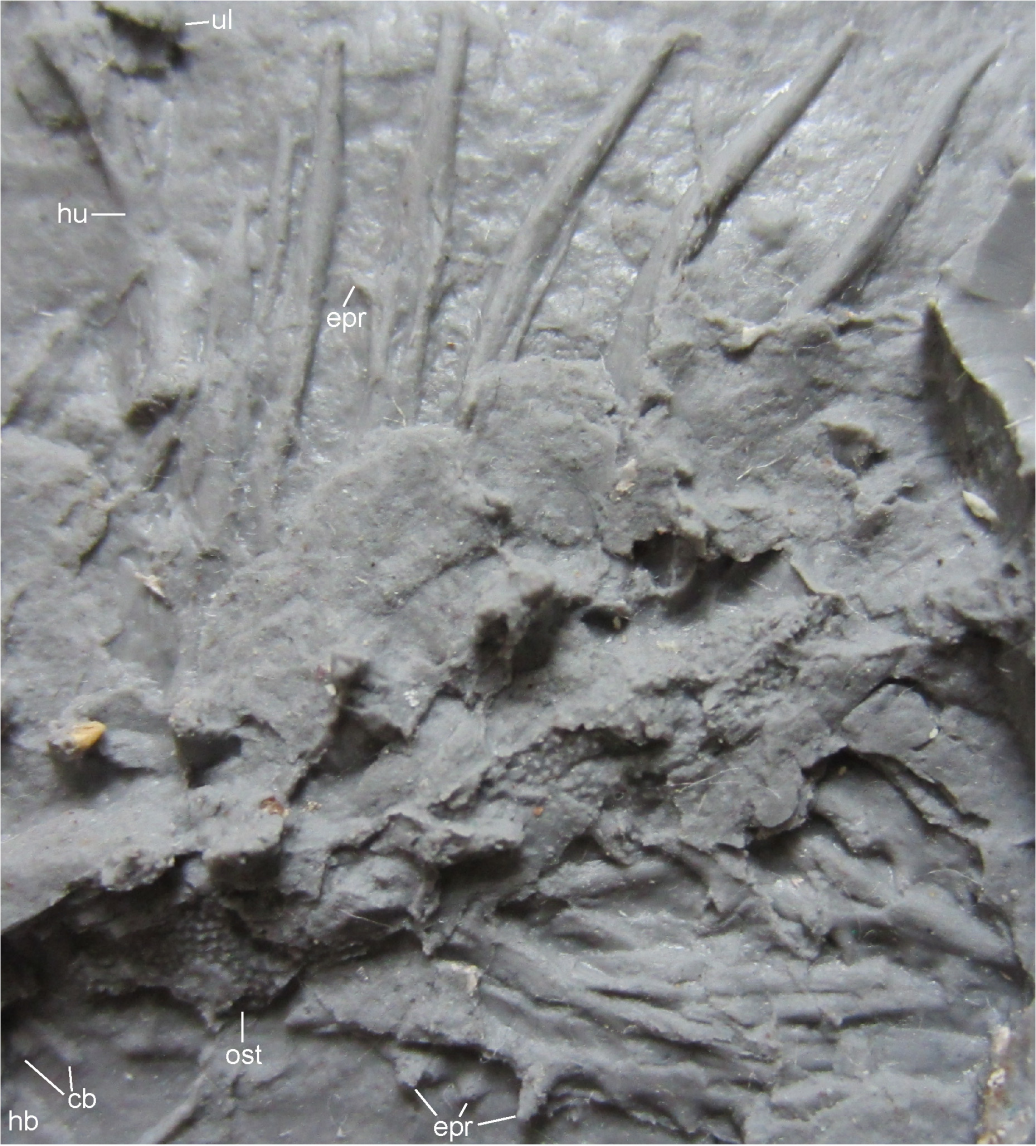
Trunk of MB.Am.45, showing epipleural spines. Photograph of MB.Am.45.3 in dorsal view, taken under daylight; anterior is to the left. Abbreviations: cb, ceratobranchial; epr, epipleural process; hb, hypobranchial; hu, humerus; ost, sculptured spine table; ul, ulna.

**Fig 14:**
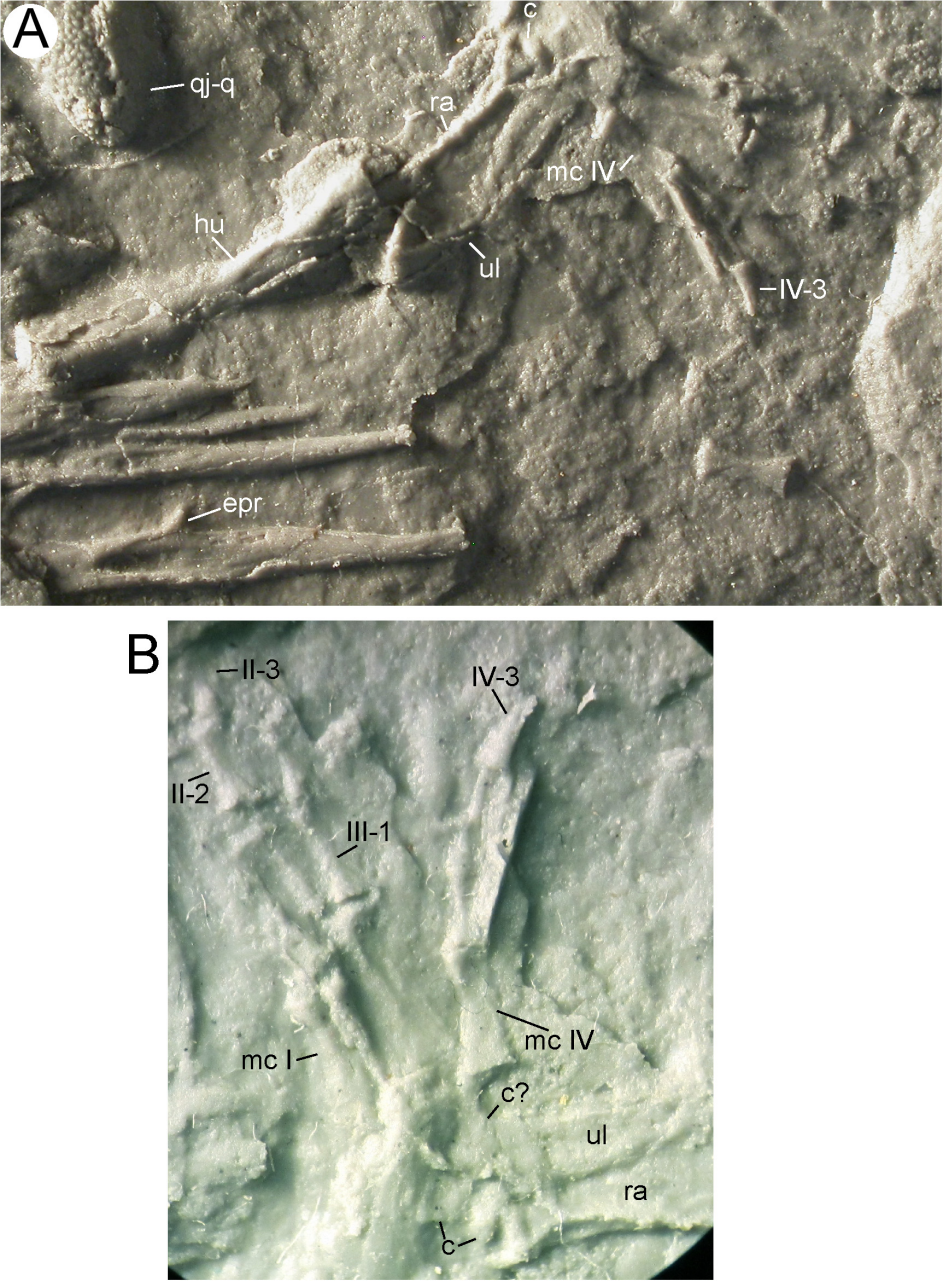
Right forelimb of MB.Am.45. Photographs of MB.Am.45.3. (A) whole forelimb, ribs and quadratojugal-quadrate bone of the right side; anterior is up. (B) close-up on the right hand taken by holding a camera to an ocular of a binocular microscope. Anterior is down and slightly to the left. At least two carpals are visible, as is the serrated crest on the clawlike terminal phalanx of digit IV. Abbreviations: c, carpal; epr, epipleural process; hu, humerus; mc, metacarpal; qj-q, quadratojugal-quadrate bone; ra, radius; ul, ulna. Roman numerals indicate digital rays, Arabic numerals indicate phalanges, so that e.g. IV-3 is the third (and terminal) phalanx of digit IV.

*Chelotriton paradoxus* ([29]: 324)

#### Material and locality

MB.Am.45 consists of the original fossil (MB.Am.45.1) and two casts (MB.Am.45.2–3). MB.Am.45.1 (Fig 4) is a natural mold of the dorsal side of a skull, anterior trunk and right forelimb in articulation apart from broken neural spines, ribs angled out of position, and some crushing and cracking. No original bone is preserved, and there is no counterpart. MB.Am.45.2 is an old plaster cast showing few details of the skeleton; the entire surface is spongy and abraded to the point of being smooth (S2 and S3 Figs). Therefore, a silicone cast (MB.Am.45.3; Figs 5 and 8–14) was made by the preparator Markus Brinkmann (Museum für Naturkunde); the following description is based on that cast, as are the specimen drawing (Fig 6) and the skull reconstruction (Fig 7). Specimen MB.Am.45.1 was found in the lignite (*Blätterkohle*, “leaf coal”) of Orsberg near Erpel, close to Linz in Rhineland-Palatinate, Germany. Apart from Orsberg, the *Blätterkohle* crops out in several localities in the Siebengebirge region near Bonn, like Erpel, Stößchen near Linz, and the famous Rott near Hennef. Based on mammal stratigraphy, the *Blätterkohle* is latest Oligocene in age; it was formed under lacustrine conditions [14,37].

#### Affinities

MB.Am.45 is referrable to *Chelotriton* based on its short, very wide skull, its bluntly pentagonal nasals that are about as wide as long [30], its peramorphic features combined with a paedomorphic hyobranchium (also seen in the Jurassic *Pangerpeton* [38]), and the mostly pustular sculpture that covers all bone surfaces in contact with the skin (including the very large spine tables of the vertebrae).

Our phylogenetic analysis further reveals three synapomorphies of MB.Am.45 with the *Chelotriton* specimens from Enspel and the Randeck Maar: a very high ratio of interorbital width to skull length, a very posterior location for the jaw articulations and very long rib-bearers (see Results and Discussion for quantification). The states of these characters are generally unknown in other *Chelotriton* material; the last, however, is shared with other vertebrae referred to *Chelotriton* (see its diagnosis above).

MB.Am.45 can be distinguished (see Results and Discussion) from *Chelotriton* material from the neotype locality of *C*. *paradoxus*, from the specimens from Enspel and the Randeck Maar traditionally referred to *C*. *paradoxus* [29,30], from “*Tylototriton primigenius*” [22], from both “*Chelotriton* sp., type I” and “*Chelotriton* sp., type II” [13,32], from tentatively referred material from Oberleichtersbach and Sandelzhausen (Germany) [19,39], and finally from *C*. *robustus* [17]. Distinction from *C*. *pliocenicus*, however, is very tenuous because of the lack of clearly overlapping material; and comparison to *C*. *ogygius*–found at the same locality–is hampered by the apparent loss of the type and only known specimen ([4]; see above) as well as by its preservation in ventral view (MB.Am.45 preserves the dorsal view). Therefore, we can neither confidently name a new species nor refer MB.Am.45 to an already known one.

### Description of MB.Am.45

#### General outline of the skull

The massive, broad-parabolic skull (Figs 4–8) is heavily ossified. It measures 18 mm in midline length (measured from the tip of the premaxillae to the posterior end of the parietals) and 35 mm in width (measured as the minimum distance between the lateral edges of the quadratojugal-quadrate bones). Thus, with a width-to-length ratio of 1.94, the skull is proportionally much broader than that of contemporary salamandrids like *Brachycormus* with a ratio of 0.96–1.07 [4] and other *Chelotriton* specimens with a ratio of 1.20–1.40 ([4]; 1.55 in the specimen illustrated in fig 2a_5_ of [29]). In *Palaeopleurodeles*, the type specimen has a skull which is broader than long, but this can be partially attributed to crushing and is not as pronounced as in MB.Am.45 [10], whereas the referred specimen [11] has a skull that is slightly longer than broad. Among extant pleurodelines, *Pleurodeles* [40], *Tylototriton* and *Echinotriton* [8] have skulls that are broader than long, but proportionally still more slender than in MB.Am.45. The quadratojugal-quadrate bones (see below) extend unusually far posteriorly and form long, slender quadrate condyles located far posterior to the skull table and the occiput (Fig 8). This character is unique among salamandrids and even among caudates, in which the quadrate condyles (forming the jaw articulation) are usually located anterior to or at the same level as the occiput [4,41]. In some specimens of *Chelotriton*, they lie slightly caudal to the occiput–never (to our knowledge) as far as in MB.Am.45 [24,29]. The considerably smaller *Salamandra “laticeps”* may come closer, but lacks the caudal projections of the quadratojugal-quadrate bones beyond the squamosals; its suspensoria are caudally rounded ([21]: plate VIII fig 2 = [4]: fig 16A). The caudal margin of the skull roof between the quadrate condyles is concave, because the caudal margins of the squamosals are inclined rostromedially to caudolaterally–the opposite is normally the case in salamanders–to connect the parietals to the quadratojugal-quadrate bones (Fig 8). On the right half of the skull, the skull-roofing bones are articulated and well discernible; on the left side, they are partially obscured by matrix. The dermal skull bones show pronounced, but regular dermal sculpture (or ornament) which is tubercular, except on the maxilla and possibly the premaxilla, where deep pits and tall ridges form “honeycombs” as seen in Palaeozoic temnospondyls [42] as well as in *Chelotriton robustus* [4,17]. Other *Chelotriton* specimens have a less regular and coarser tubercular sculpture ([13,19,32,39]; [29]: figs 2a_5_, 2b_1_). Prominent crests as in *Tylototriton* or *Echinotriton* [8] are absent. The dermal sculpture of MB.Am.45 (Figs 8–11) is described for each skull bone separately below. The external naris is a small, elongate-ovate opening, with the longitudinal axis directed anteromedially. It is bordered anteriorly by the premaxilla, posteromedially by a bone of unclear identity (see below), posteriorly by the nasal, and laterally by the alary process of the maxilla. The orbitotemporal fenestrae (or orbits) are large and directed anteromedially, measuring approximately 14 mm in length and approximately 5 mm in maximum width. They are bordered by the maxilla anterolaterally and laterally, the quadratojugal-quadrate bone posterolaterally, the squamosal posteriorly and posteromedially, the frontal medially and the prefrontal anteromedially. The frontosquamosal bar or arch (*arcus frontotemporalis*) is well developed and unusually broad, almost attaining the width of the orbitotemporal fenestra. Its width is at least comparable to that of the highly ossified skulls commonly referred to *Chelotriton paradoxus* ([22]: fig 4a; [29]: figs 2a_5_, 2b_1_), clearly surpassing *Tylototriton* and *Echinotriton* ([8]: fig 1), and much larger than in *Brachycormus* ([4]: fig 20; [14]). A small, squarish postorbital opening (or supratemporal fenestra) is separated from the orbitotemporal fenestra by the frontosquamosal bar; the opening is bordered anteriorly by the frontal, laterally and posterolaterally by the squamosal and posteriorly and medially by the parietal. It is proportionally smaller than in any other pleurodeline, extant or extinct. The interorbital width cannot be measured with accuracy because the outline of the left orbitotemporal fenestra is not preserved. However, the shortest distance between the medial margin of the fenestra and the interfrontal suture is approximately 7 mm, thus indicating an interorbital width of about 14 mm and a ratio of interorbital width to skull length of 0.78. In the large skull figured in fig 2a_5_ of [29] (referred to *C*. *paradoxus*), the ratio is 0.54, and in the *Brachycormus* skull illustrated as: fig 20a of [4] it is 0.51.

#### Skull-roofing bones

*Premaxilla*. Of the premaxillae, only the anteroposteriorly very short anterodorsal portion is exposed (Figs 9 and 10). Short, rather blunt, triangular alary processes (preserved vertical) are developed that overlap the anteriormost part of the nasals. The alary processes are widely separated from each other and from the median suture between the nasals, even at their bases (Fig 7). They are located medial to the external nares, but do not form their medial margins. As can be judged from the surface of the alary processes, the dermal sculpture of the premaxillae appears to be composed of pits and ridges. Because of incomplete preservation, it cannot be ascertained if the premaxillae were fused ventrally or not, although their anterior surfaces are clearly sutured. Teeth are exposed neither on the premaxilla nor on the maxilla.

*Maxilla*. The maxilla is the longest bone of the skull roof, with a length of approximately 17 mm. Its broad alary process (Figs 8–10) constitutes about the anterior third of the bone and bears a pronounced dermal sculpture of pits in the centre and radiating ridges and furrows in the anterior portion. The maxilla is connected with the premaxilla anteriorly by a short suture and with nasal and prefrontal by a convex suture medially. The posterior portion of the maxilla (dental shelf) is robust and forms an oblique suture with the quadratojugal-quadrate bone (Fig 8). Its surface is partially covered by sediment; where it is exposed, small tubercles which are connected by anteroposteriorly aligned, low ridges are visible.

*Unidentified bone*. A small, semilunate bone with a smooth surface borders the external naris anteromedially (Figs 9 and 10). It is placed more internally than the nasal to which it is connected posteromedially. The suture between the two bones is marked by a groove and might indicate a rather weak connection. The bone appears to have a ventromedial process directed into the external naris. At least three interpretations of its homology are possible. First, this bone could represent the septomaxilla. However, according to [4,30,43] and pages 6–7 of the supplementary information of [44], the septomaxilla is absent in all salamandrids. The septomaxilla has also not been described in the well-ossified skulls of extant and extinct pleurodelines [8,22,34,40], and would be expected to lie in a caudolateral instead of rostromedial position. Dr. Márton Venczel has pointed out to us (pers. comm.) that one of the many processes of the complex septomaxilla of *Salamandrina* is similar to the condition we present here. Such a process has not been reported elsewhere in *Chelotriton* or in any other salamandrid, and, as Dr. Venczel stated, its position is not identical (as far as the compressed condition of the specimen allows one to state). Finally, Dr. Jason Pardo (pers. comm. to DM) has suggested that the structure in question could be an upturned part of the vomer, contributing to an internasal septum. However, the nares lie far apart from each other in MB.Am.45; the vomers could in principle have contributed to the lateral wall of the nasal capsules, but there would have been a great amount of space between these contributions. Given the preservation of the specimen as a natural mold, we cannot decide between these options. If the bone in question is the septomaxilla, its unexpected presence in MB.Am.45 might be a further indicator of extreme peramorphosis: wherever the septomaxilla is present in urodeles, it is one of the last skull bones to ossify [43].

*Nasal*. The nasal (Figs 8–10) is a pentagonal bone, slightly longer than wide, reaching its maximum width on the level of the anterior tip of the prefrontal. It is connected anteriorly to the premaxilla, medially to its counterpart (there is no median fenestra or fontanelle), anterolaterally to the unidentified bone, and laterally to the alary process of the maxilla. Its suture with the prefrontal runs anterolaterally to posteromedially. The dermal sculpture of the nasal consists uniformly of tubercles. Most of the tubercles are interconnected by low ridges, especially in the anterior part of the bone, giving the sculpture a reticulate appearance.

*Frontal*. The frontal (Figs 8, 9 and 11) is slightly longer than wide. It is connected anteriorly with the nasal and medially with its counterpart by rather straight sutures. Posteriorly, it sutures with the parietal and forms the anterior edge of the postorbital opening. It is connected to the squamosal posterolaterally, forming the extensive frontosquamosal bar. In this region, the frontal attains its greatest width. Laterally, the frontal constitutes the medial edge of the orbitotemporal fenestra. Anterolaterally, the frontal forms an anteromedially-to-posterolaterally directed suture with the prefrontal that is difficult to trace (but see Figs 8 and 9). The frontal is only slightly longer than the nasal, but has 1.3 times its width. The dermal sculpture of the frontal corresponds to that on the nasal. However, the size of the tubercles decreases in the posterior portion of the bone, and also the interconnecting ridges become lower and may be absent. The posteromedial portion of the frontosquamosal bar adjacent to the postorbital opening is concave dorsally and rather smooth with the exception of scattered, low tubercles (Fig 11).

*Prefrontal*. The prefrontal (Figs 8–10) is a rhombic bone that borders the orbitotemporal fenestra anteromedially. It has its greatest width on the level of the frontal-nasal suture and tapers anteriorly to terminate at about midlength of the nasal. It forms oblique sutures with the nasal anteromedially and the frontal posteromedially, and a medially convex suture with the alary process of the maxilla. The bone becomes thicker towards the margin of the orbitotemporal fenestra. The dermal sculpture is tubercular, but the interconnecting ridges are rather high, similar to the anterior portion of the nasal.

*Parietal*. The parietal (Figs 8, 9 and 11) is a roughly rectangular bone, wider than long, with an anterolateral embayment that forms the lateral and posterior margin of the postorbital opening (or supratemporal fenestra). It is connected with the frontal anteriorly by a rather straight mediolateral suture. The suture with the squamosal is difficult to follow; in our interpretation, it extends in an anteroposterior direction from the posterolateral corner of the postorbital opening to the posterior margin of the skull table, coinciding with a crack (Figs 8 and 11). The portion medial and posteromedial to the postorbital opening is dorsally concave as preserved, and the dermal sculpture consists of isolated, low tubercles. In contrast, the portion directly posterior to the postorbital opening is flat and has a sculpture composed of low but very wide tubercles which are connected by ridges, forming something of a subdued honeycomb pattern, although these ridges are much lower and much more rounded than on the maxilla, and the individual tubercles still form peaks between which the ridges are distinctly lower still.

It is not clear if there was an unornamented trough between these two sculptured areas, coinciding with another break (Figs 8 and 11). Such a trough, along which jaw-closing muscles passing through the supratemporal fenestra would have been able to attach to neural spines, appears to be present in all other salamandrids that have ornamented parietals and/or squamosals, including other *Chelotriton* specimens [30] and *Brachycormus* ([4]: fig 20). In MB.Am.45, however, such a trough would have had to be extremely narrow, because the concave medial portion with its sculpture curves some distance laterodorsally into the break (mostly visible in Fig 11).

*Squamosal*. The squamosal (Figs 8 and 11) forms the posterior and posteromedial border of the orbitotemporal fenestra and, together with the parietal, the concave posterior margin of the skull roof between the quadratojugal-quadrate bones. It sutures medially with the parietal and laterally with the quadratojugal-quadrate bone. Anteromedially, it meets the frontal to form the robust frontosquamosal bar. The surface of the squamosal is dorsally convex lateral to the orbitotemporal fenestra, and this convexity extends onto the lateral part of the frontosquamosal bar (whereas the medial part of the bar is dorsally concave, see above). The dermal sculpture of the squamosal consists uniformly of tubercles that may be connected by low ridges. The dorsally convex lateral part of the frontosquamosal bar has pronounced tubercles that are connected by ridges which are aligned in parallel to the margins of the bar, whereas the sculpture is subdued in the concave medial part of the bar.

*Quadratojugal-quadrate bone* (Figs 4, 5 and 8). There has been some confusion concerning the designation of the bone posterior to the maxilla which forms the jaw joint in caudates. It is termed either a quadratojugal or quadrate by different authors. Some sources [4,15,29] termed the large, sculptured bone that is in broad contact with the posterior end of the maxilla in *Chelotriton* the quadratojugal, whereas [22] interpreted this bone in the same taxon as the quadrate. A bone in a comparable position was referred to as the quadrate in *Tylototriton* and *Echinotriton* [8,33,45], and a separate quadratojugal bone was described neither in these nor in other pleurodelines. In most anatomical studies dealing with the salamander skull, a quadratojugal bone is not identified. However, the presence of a distinct quadratojugal is a plesiomorphic feature in caudates, found in the Late Jurassic *Karaurus* [46]. In crown-group salamanders (urodeles), it has been shown that quadratojugal and quadrate have separate ossification centers and fuse early in ontogeny, thus forming a compound bone of dermal and endochondral origin ([43] and references therein). For this reason, and because the bone in question in MB.Am.45 most likely has a large dermal component with a sculptured surface while a separate quadrate cannot be observed, we think it is most appropriate to term it the quadratojugal-quadrate bone. The quadratojugal-quadrate bone is an elongate element whose anterior border cannot be determined with certainty since the skull lateral to the orbitotemporal fenestra is crushed and partially covered with sediment, although it clearly appears to meet the maxilla (Fig 8). According to our interpretation, the anterior tip of the quadratojugal-quadrate bone extends almost to the midlength of the orbitotemporal fenestra, where it forms an anteromedially directed suture with the maxilla. It is medially connected to the squamosal (Figs 5 and 8), and the medial part of the anterior portion forms the posterolateral border of the orbitotemporal fenestra (Fig 8). The quadratojugal-quadrate bone forms a distinct posterior quadrate condyle (Figs 4, 5 and 8). The lateral margin of the condyle is convex, the medial one is straight. The quadrate condyle has a dorsally convex surface which is densely covered by tubercles that are smaller than those of the other bones. In the other parts of the quadratojugal-quadrate bone, the bone surface is poorly preserved but the tubercles seem to be larger and less densely arranged. In *Chelotriton* from Enspel and the Randeck Maar, this bone bears anteriorly directed spines similar to the single spine of *Echinotriton* [30]. There is no evidence for such spines in MB.Am.45 (Figs 4 and 5), but this may be due to the preservation in dorsal view: in at least one Randeck specimen, the spines are only visible in ventral view ([30]: figs 1, 4a, 4b). Perhaps more importantly, the lateral surface of the impression of the quadrate condyles has broken away on both sides, possibly obscuring any spines (Fig 4).

#### Other parts of the head skeleton

*Palate*. Almost nothing can be said about the palate. A rather smooth, triangular piece of bone in the right orbitotemporal fenestra (Figs 8 and 11) is connected posteromedially to a fragment of a thicker bone. This could represent the ventromedial process of the maxilla that was sutured to the pterygoid, as in *Tylototriton* [22] or *Echinotriton* [8,45].

*Exoccipital and opisthotic*. Posterior to the hind margin of the parietals and the medial portion of the squamosals is a crushed mass of bone with an irregular outline (Figs 8, 11 and 12). Because of its location posteromedial to the skull table, at the anterior end of the vertebral column, it probably represents the remnants of the exoccipitals and the otic capsules (compare [30]). However, nothing can be said about their morphology.

*Hyobranchium*. In the terminology of the hyobranchial remains, we follow comparative osteological and myological studies [47] which have demonstrated that it is most parsimonious to designate the proximal-most elements of the branchial arches in salamanders as hypobranchials and the distally following segments as ceratobranchials. This view is in accordance with [30,34] and the supplementary information of [44] as well as with the likely homology of comparable elements in other tetrapods ([48] and references therein), but stands in contrast to [3,14,15,29,49–52], among others, who preferred the usage of the terms ceratobranchial and epibranchial for the two segments of the branchial arches that remain in salamanders of the original four.

In MB.Am.45, the distal ends of a broad, flattened rod and of two smaller, rod-like bones posterior to it are exposed posterolateral to the presumed exoccipitals/otic capsules (Figs 5, 8 and 13, S2 Fig, S3 Fig). According to the location posterior to the skull, the anterior, large bone can be interpreted as an ossified hypobranchial, and the posterior rods most likely represent ossified ceratobranchials. If this interpretation is correct, the hyobranchial apparatus of MB.Am.45 was not metamorphosed. Rather, some of the larval branchial arches were retained and ossified, thus representing a paedomorphic condition. Specimens currently referred to *Chelotriton paradoxus* were originally interpreted as having a metamorphosed hyobranchial apparatus consisting of the ossified ceratohyal, hypobranchial 1 and ceratobranchial 1 on each side [13–15], but are more recently considered to have an unmetamorphosed or incompletely metamorphosed hyobranchium, of which hypobranchial 1, ceratobranchial 1 and ceratobranchial 2 were ossified on each side [29,30]; the latter interpretation is consistent with MB.Am.45.

*Brachycormus* appears neotenic in possessing two ossified pairs of hypobranchials and four ossified pairs of ceratobranchials [14,50].

#### Postcranium

Of the postcranium, the anterior part of the trunk with vertebral fragments, ribs and the right forelimb are preserved.

*Vertebrae*. The vertebrae are poorly preserved and provide little information concerning their morphology (Figs 5, 12 and 13). Parts of the vertebral centra are only visible in the presumed atlas and the first trunk vertebra. Otherwise, only fragments of the rib bearers (diapophyses, parapophyses) are preserved. The neural arches of two vertebrae (?the 4^th^ and 5^th^ trunk vertebrae) are preserved in dorsal view, showing the neural spines and articulated pre- and postzygapophyses (Fig 6). The spine tables have broken off, several fragments lie dorsal to the? atlas and lateral to the subsequent vertebrae (Figs 5, 12 and 13). The shape of the fragments is ill-defined, but they seem to have been longer than wide. Their dorsal surface bears tubercular dermal sculpture as on most of the skull-roofing bones. Similar sculptured spine tables have been described in a variety of other salamandrids, extant and extinct (e.g. [4,9]). In specimens traditionally referred to *Chelotriton paradoxus*, the spine tables may be elongate or heart- or chevron-shaped and bear a coarse pustular sculpture [4,29], whereas in *Chelotriton robustus* they form wide plates with a sculpture that consists of pits and very fine pustules [4,17]. In *Brachycormus*, the sculpture on the spine tables is also pustular [4], and the spine tables are strikingly slender and bear pits and furrows in *“Tylototriton” weigelti* [33]. Among extant pleurodelines, both *Tylototriton* and *Echinotriton* have pustular ornament on the spine tables [8,9].

*Ribs*. At least eight pairs of ribs whose proximal portions are not preserved are spread out on the right side (Figs 4, 5, 12 and 13, S2 Fig) and bunched together on the left side (Figs 4, 5 and 13, S2 Fig). The remains of the ribs are long and attain the length of more than three successive vertebrae. Taking into account the missing proximal portion, they were proportionally longer than the elongate ribs of the Enspel specimens of *Chelotriton* [29], as well as those of *Echinotriton* [8,45] or *Palaeopleurodeles* [11]. The first four of the preserved right ribs are straight, whereas the posteriorly following ones are slightly curved ventrally. At their distal ends, the ribs taper to a pointed tip. No bifurcated distal end is preserved. On the right side, the 4^th^ to 6^th^ preserved ribs exhibit one small epipleural (or tubercular) process. On the 4^th^ and the 6^th^ preserved rib, it is located at about the middle of the shaft, whereas it is situated more distally on the 5^th^ preserved one. The left side shows, however, that this is an artefact of exposure in what seems to be posteroventral view. Several left ribs, preserved in posterior view, have three or more epipleural processes (Fig 13), similar to Enspel specimens of *Chelotriton* as illustrated in fig 1 of [29] and to *Echinotriton* [8]; one even has at least five sizable spines (Fig 13), reminiscent of isolated ribs referred to “*C*. aff. *pliocenicus*” [19] which have up to ten such processes. Epipleural or tubercular processes are not confined to pleurodelines, but are widespread among extant and fossil salamandrids, occasionally occur in extant *Salamandra*, and may be present in other groups of caudates as well [4,8,33,53].

*Forelimb*. Only the right forelimb can be identified (Figs 5 and 12–14). The humerus (Figs 12–14A) is a slender, straight bone measuring 12 mm in length. The deltopectoral crest (or ventral humeral crest) appears to be of moderate size, but only its distal part is preserved. The distal end of the humerus is slightly widened; the ulnar trochlea and the radial condyle are missing and were probably cartilaginous in the living animal–indeed, the radius has been pushed into the hollow distal end of the humerus post mortem (Fig 14A). Unlike in the Enspel and Randeck specimens of *Chelotriton* [30], the ulna forms a widened olecranon (Figs 12–14A). The bone measures 8 mm in length, thus the ratio of humerus to ulna length is 1.5. The radius measures about 7 mm. Its distal end appears to be only slightly widened. At least two carpal elements are ossified (Fig 14). One is preserved immediately distal to the distal end of the radius, the other one is located proximal to the first metacarpal. Other parts of the carpal region are obscured by matrix, and it is highly probable that further carpal elements were ossified. Among pleurodelines, ossified carpals have been found in *Brachycormus* [4,14], *Echinotriton* [8,45], *Chelotriton robustus* [17], and large individuals that were referred to *C*. *paradoxus* [24,29,30]. Four metacarpals are preserved (Fig 14). The phalangeal formula was? 2-3-?3–3, conforming to the formula 2-3-3-3 found in *Chelotriton* from Enspel and the Randeck Maar [30]. The preserved fingers are remarkably long. One phalanx, the proximal one, is preserved in the first finger; the second finger is almost complete and consists of three phalanges, with the tip of the terminal phalanx broken off with the surrounding matrix (Fig 14B). Two phalanges are preserved in the third finger, and the fourth one is again complete with three phalanges including a very elongate, clawlike terminal phalanx (Fig 14) which appears to have a coarsely serrated lateral crest (Fig 14B); inspection of MB.Am.45.1 does not clarify whether the serration is an artefact of the casting process, but the crest as such appears to be real.

### Phylogenetic analyses

The matrix (Table 1; S1 Data) was compiled in Mesquite 2.75 [54]. We performed the analyses in PAUP* 4.0a136 [55] on a computer with an Intel Core2 Duo CPU (2.66 GHz) and 3.87 GB of usable RAM. The heuristic searches were constrained to 1000 replicates of stepwise addition with random addition sequence, each followed by unlimited TBR (tree bisection and reconnection).

**Table 1:** Data matrix for phylogenetic analysis of Salamandridae. This is a human-readable version of S1 Data. Parentheses indicate polymorphism, partial uncertainty is shown by curly braces. The characters are listed in the Appendix.

Characters up to	5	10	15	20	25	30	35	40	45	50	55	60	65	70	75	80	85	90	95	98
*Ambystoma*	0?0??	0(01)?00	(01)01(02)2	20?0(01)	?00(01)1	0(01)0(01)0	(01)(01)003	0100(12)	002(01)0	0000?	(01)???0	?0000	(01)0111	100(12)1	010(01)?	???00	?0000	00002	00000	0?0
*Dicamptodon*	211?1	10?00	11111	10?00	?0001	11000	01003	00002	00100	00?0?	1????	?000?	?????	?????	?1???	???00	?0000	000?2	{01}????	??0
*Salamandra*	0?0??	0100(01)	0001(01)	(12)0000	?0010	00000	0000(01)	00102	00011	?0020	00003	00102	00111	21000	(01)1000	??000	?2102	000(12)0	?0000	0?0
*Chioglossa*	0?0??	11003	???01	11?00	?0010	10000	00000	?2100	00011	?1010	10013	012?2	00111	22000	01??0	??000	?2001	10010	?????	??1
*Salamandrina*	10101	11001	1?12(01)	(12)01{12}0	22000	01110	00101	01100	20011	?1020	00011	01200	10000	11100	00??0	??011	??000	00002	20000	0?1
*Tylototriton*	21211	00003	??010	(01)0?12	22000	11110	10002	01001	11000	10101	20100	10011	21000	12111	11101	20001	00001	0111(02)	21000	0?2
*Echinotriton*	21211	00?03	???00	00?12	22100	11111	?0???	0{01}00?	11??0	?01?1	201??	1??10	21000	11101	10?0?	20001	?200?	?????	?????	??2
*Pleurodeles*	212(01)1	01003	1?001	1111(12)	22110	1(01)100	(01)0001	01002	21200	11011	20103	00012	01110	12101	10101	20001	?0000	01012	21000	0?1
*Notophthalmus viridescens*	21211	1111(03)	111(02)1	11?12	22010	10110	01112	021?1	21100	01101	22102	20011	21000	10110	01001	01?01	01001	000(02)1	12000	000
*Taricha torosa*/*granulosa*	21211	10110	00(01)21	2111(12)	22010	1(01)111	0100(12)	1210(12)	21200	0(02)101	22003	2(01)002	20000	21111	01001	01?01	01011	00021	10000	000
*Euproctus*	(01)01?0	11?1(012)	(01)0001	{12}1?10	(012)(12)(01)11	1(01)(01)10	01011	0010?	(12)?(02)??	?????	?2???	??(01)11	00101	11101	0100?	01?11	0?0(01)2	0003?	?????	??1
*Cynops*	20211	11112	1?1(01)2	(12)1?12	22(01)1(01)	11110	(01)1112	12000	21200	13101	22103	2101(12)	2(01)(01)00	(12)(01)120	01011	(01)1101	(01)2011	10001	02000	0?2
*Paramesotriton*	2021?	11?10	1000(01)	(01)1?11	2211(01)	10110	?1112	1{12}100	21000	03101	211?2	20011	21000	12121	01??1	10?01	12011	100(03)1	02000	0?2
*Pachytriton*	102?(01)	0(01)?10	10012	0(01)?{01}2	(12)2111	1(01)110	1?(01)12	?2000	21200	13100	22103	20?11	00110	1111(01)	01001	10100	12011	00101	02000	0?2
*Neurergus*	10100	0(01)11(12)	10122	2101(01)	(02)1010	1(01)111	(01)0(01)0(01)	200?0	21100	03101	12103	2001(012)	(01)0(01)00	11101	?1??1	01?01	02??1	???01	12000	001
*Ommatotriton*	0?1??	0111(12)	1?1(12)(12)	(12)101(12)	(12)2010	11110	11(01)(01)2	2210?	21??0	?31?1	2?1??	2??11	00(01)(01)(01)	111(012)(01)	0?01?	??101	0?01?	???01	1?300	011
*Calotriton*	101?0	11110	10002	00?20	(12)2111	11110	11011	21100	21200	13101	22103	20011	01111	11100	01???	01111	12112	0003?	?????	??1
*Triturus marmoratus*	11200	0111(01)	(01)00(12)2	1101(01)	0(01)010	1(01)011	1111(12)	21100	?????	?????	?2???	???12	01000	11101	0101?	00???	01???	???01	15310	011
*T*. *(Triturus)*	(12)0(12)00	0111(12)	100(01)2	(12)1110	0(01)010	110(01)0	011(01)(012)	22100	21000	13101	22102	21012	00(01)(01)0	2110(01)	01011	00101	01011	10001	15310	001
*Lissotriton vulgaris*	0?0??	01110	11122	10111	02010	10010	(01)1112	221??	?????	?????	?2???	???12	00100	02101	0?01?	00???	0?011	10001	14101	111
*L*. *montandoni*	201??	11110	01021	10122	(12)2010	1001?	01102	221??	?????	?????	?????	???11	10100	00?00	0?01?	?????	0????	???01	14101	111
*L*. *italicus*	0?1??	11110	01022	11122	22110	1111?	11112	221??	?????	?????	?????	???1?	?????	?????	0?01?	?????	0????	???01	13002	111
*L*. *helveticus*	0?1??	01110	(01)(01)(01)21	101{12}2	22110	10010	0101(12)	2210?	21??0	?31?1	2?1??	2??11	10100	00110	0????	??101	0?01?	???01	14101	111
*L*. *boscai*	10100	(01)1110	0(01)0(12)2	(12)001(12)	(012)(12)010	1(01)(01)11	11(01)0(12)	22100	21{12}10	13???	?????	???11	10000	11110	0100?	?????	0?011	10001	13002	011
*Ichthyosaura alpestris*	10100	01(01)11	10122	21010	011(01)0	00010	11112	22100	21000	13101	22102	21011	00100	21111	01011	00101	01011	10001	12201	011
?*I*. *randeckensis*	10110	01?10	10112	21012	02110	1001?	1111?	?21??	?????	?????	?????	???10	0?00?	???0?	0?01?	?????	?????	?????	?????	??1
*Carpathotriton*	{12}0101	???10	0??{12}?	2000?	22100	10111	11???	0??1?	?1???	?????	?????	???1?	2110?	00020	?????	?????	?????	?????	?????	??1
*Archaeotriton*	10100	01100	00012	21010	22110	00100	?????	?2?1?	?1?01	?????	?????	???0{01}	1100?	210{01}0	0?00?	?????	?????	?????	?????	??1
MB.Am.45	21211	00?03	???00	00?{12}3	22100	111??	?????	?0???	?????	?????	?????	?????	2????	???2?	1????	?????	?????	?????	?????	??1
Enspel/Randeck *Chelotriton*	21211	00?03	???00	00?13	22100	1110?	1000{12}	?010?	?????	?????	?????	???10	2?000	???2?	1000?	?????	?????	?????	?????	??1
*Brachycormus*	21211	0(01)?1{01}	1???0	0?0{12}3	221?0	?????	???01	?{12}???	?????	?????	?????	???1?	2????	{12}????	0??0?	?????	?????	?????	?????	??1
*“Triturus” roehrsi*	2021?	?????	?????	???2?	22??0	?????	?0???	2????	?????	?????	?????	????1	20(01)00	11120	?????	?????	?????	?????	?????	??1
?*Taricha miocenica*	?????	?????	?????	?????	?????	?????	?????	?????	?????	?????	?????	????1	20000	20120	?????	?????	?????	?????	?????	??0
?*Notophthalmus crassus*	?????	?????	?????	?????	?????	?????	?????	?????	?????	?????	?????	????1	{12}1000	{01}0120	?????	?????	?????	?????	?????	??0
?*N*. *robustus*	?????	?????	?????	?????	?????	?????	?????	?????	?????	?????	?????	????1	20000	20121	?????	?????	?????	?????	?????	??0
*Koalliella*	?????	?????	?????	?????	?????	?????	?????	?????	?????	?????	?????	????1	10010	2112(01)	?????	?????	?????	?????	?????	??1

We ran four phylogenetic analyses. Two were unconstrained, two were constrained by the current consensus derived from molecular data; one of each was performed with the additional character 98 included.

#### Method of analysis

For a long time, simple parsimony was the only available method for phylogenetic analysis of morphological data. Bayesian analysis is now a strong contender; it has the advantage of being less sensitive to long-branch attraction, and the fear of several potential disadvantages has been shown to be unfounded [56]. However, contrary to popular belief, [56] has not shown that Bayesian analysis can be generally expected to outperform parsimony. Firstly, [56] only tested binary characters; our matrix contains both ordered and unordered multistate characters in addition to binary ones, as well as characters to which more complex stepmatrices apply. We are not aware of a similar test using multistate characters. Secondly, fig 6 of [56] showed that the difference in topological error committed by the two methods decreases for datasets with high average evolutionary rates, in other words for noisy datasets. Real datasets, including the ones used in our analyses, are very noisy: the shortest trees found by our analyses (see the Results section) have consistency indices below 0.5, meaning that the average character state change happens more than twice per tree. For these reasons, we have analysed both of our matrices only with parsimony, although we concur with [56] that Bayesian analysis shows great potential–it should be tested further.

### Character and taxon sample.

“I have *exclusively* considered the bony skull in the present work.”

[57] (footnote on page 260; our translation, italics in the original)

“While anatomical studies of salamanders are not new, most have been done on a relatively few species and are mostly pure descriptive morphologies or superficial comparisons of many species. Somewhat surprisingly, the common California Newt (Taricha torosa) has not been fully nor accurately described. This study provides a detailed description of the head and neck anatomy of T. torosa.”

[58] (page vii; underlining in the original)

“With the growing application of DNA-sequence and other genetic data since the 1990s, osteological work has largely given way to molecular approaches in the study of amphibian biodiversity. Many influential works from the mid-20th century (e. g., Francis, 1934; Tihen, 1958; Hansen and Tanner, 1958; Wake, 1963; Özeti and Wake, 1969) are still cited today, but few such works are produced anew by contemporary biologists. However, as an independent source of data, osteological characters can provide invaluable insights into evolutionary relationships of living taxa that are as important as those derived from nucleotide substitutions.”

([59]: 83)

To evaluate the phylogenetic position of MB.Am.45 with respect to other newts, we have performed four phylogenetic analyses of Salamandridae on a data matrix we have created by merging those of [3,9,51,52,60,61], with two additional characters from the apomorphy list of [16], three from [48], two from [62] and one from tables I and II of [63]. For an additional set of analyses, we added geographic distribution as a new character. The complete list of characters, together with characters in these sources that we ended up excluding from our matrix, is presented in the Appendix. The matrix (Table 1, identical to S1 Data) has 36 OTUs and 97 or 98 characters (all of them parsimony-informative, many of them multistate), making it by far the largest nonmolecular data matrix ever used to analyse salamandrid phylogeny. Most of the characters cover the morphology of the skull, the trunk vertebrae, the skeleton and musculature of the hyobranchial apparatus, cloacal anatomy and courtship behavior; one character each describes the lower jaw, the ribs, the ilium, the tarsus, and the skin. We filled in missing data as much as possible (Table 2 tracks the changing names of species in our sources), and compared most of the existing scores of skull anatomy in particular to published descriptions; all changes to cells that were previously scored are mentioned and justified in the Appendix, as are all scores that seem borderline or otherwise arguable. Much of our information comes from sources even older than those cited in the quote from [59] above: [57] from 1928 and even [49] from 1877. Unfortunately, these–as well as more recent ones like the important [40]–are resolutely craniocentric, mostly disregarding the hyobranchium, the postcranial skeleton and the soft tissues. We were able to fill in the cloacal characters for a few species based on [64–66] and some other missing information based on [67–82].

**Table 2:** Salamandrid synonymies. This table shows how we have interpreted taxon names in our sources; it is not intended to express any taxonomic or nomenclatural opinion beyond acceptance of the conclusions of the most recent sources. Empty cells mean that a source did not mention a taxon. *Bradybates*, mentioned by [21,49] but not shown here, is *Pleurodeles* [85]. Symbols as in the Appendix: AS, [61]; SR, [60]; TL, [52]; V, [3]; WÖ, [51]; YZ, [9]. The abbreviations for taxon names are consistent in each line and spelled out in the last applicable column; note that *T*. means *Triturus* throughout this table, never *Triton*. The *Notophthalmus* and *Taricha* OTUs do not include the extinct species referred to these genera (see Table 1 and text).

[49] (1877)	[57] (1928)^1^	WÖ (1969)^2^	AS (1989), [40] (1994), TL (1995)^3^	[78] (2007), V (2008), [1] (2008), [83] (2009)	YZ (2012)	SR (2013)^2^	[2] (2014)	[84] (2014)	[85] (2014)	OTUs in this work
*Triton v*.	*Diemyctylus v*. *v*.	*N*.	*N*. *v*.	*N*. *v*.	*N*. *v*.	*N*.	*N*. *v*.	*N*. *(N*.*) v*.	*N*. *viridescens*	*Notophthalmus*
*Triton torosus*	*D*. *torosus*	*Ta*.					*Ta*. *t*.	*Ta*. *(Ta*.*) t*.	*Ta*. *torosa*	*Taricha*
			*Ta*. *g*.	*Ta*. *g*.	*Ta*. *g*.		*Ta*. *g*.	*Ta*. *(Ta*.*) g*.	*Ta*. *granulosa*	
	*E*. *m*.	*E*.		*E*. *m*.		*E*.	*E*. *m*.	*E*. *m*.	*E*. *montanus*	*Euproctus*
*Triton p*.	*E*. *Rusconii*			*E*. *p*.	*E*. *p*.		*E*. *p*.	*E*. *p*.	*E*. *platycephalus*	
*Triton subcristatus*	*C*. *p*.	*C*.	*C*. *p*.	*C*. *p*.	*C*. *p*.	*C*.	*C*. *p*.	*C*. *(C*.*) p*.	*C*. *pyrrhogaster*	*Cynops*
	*C*. *Wolterstorffi*	*H*.	*Hypselotriton*	(by implication *C*.)	*H*. *w*.		(by implication *C*.)	*C*. *(Cynotriton) w*.	*H*. *wolterstorffi*	
	*Triton (Palaeotriton) v*. *ciliciensis*	*Triturus*	*T*. *(T*.*) v*. *v*.	*O*. *v*.	*O*. *v*.	*O*.	*O*. *v*.	*O*. *v*.	*O*. *vittatus*	*Ommatotriton*
			*T*. *(T*.*) v*. *o*.	*O*. *o*. *o*.			*O*. *o*.	*O*. *o*.	*O*. *ophryticus*	
	*Triton (Neotriton) m*.		*T*. *(T*.*) m*.	*T*. *m*.	*T*. *m*.	*T*. *m*.	*T*. *m*.	*T*. *(Pyronicia) m*.	*T*. *m*.	*Triturus marmoratus*
*Triton c*.	*Triton (Neo*.*) c*. *c*.		*T*. *(T*.*) c*.	*T*. *c*.		*T*. *c*.	*T*. *c*.	*T*. *(T*.*) c*.	*T*. *cristatus*	*Triturus (Triturus)*
	*Triton (Neo*.*) ca*. *ca*.		*T*. *(T*.*) ca*.	*T*. *ca*.			*T*. *ca*.	*T*. *(T*.*) ca*.	*T*. *carnifex*	
	*Triton (Neo*.*) c*. *danubialis*		*T*. *(T*.*) dobrogicus*	*T*. *d*. *d*.			*T*. *d*.	*T*. *(T*.*) d*.	*T*. *dobrogicus*	
	*Triton (Neo*.*) ca*. *Karelinii*		*T*. *(T*.*) karelini* (sic; AS, TL)	*T*. *k*. *karelinii*			*T*. *k*.	*T*. *(T*.*) k*.	*T*. *karelinii*	
								*T*. *(T*.*) i*.	*T*. *ivanbureschi*	
				*T*. *k*. *arntzeni*					^4^	
							*T*. *mc*.	*T*. *(T*.*) mc*.	*T*. *macedonicus*	
*Triton taeniatus*	*Triton (Pal*.*) v*. *v*.^5^		*T*. *(Pal*.*) v*. *v*.	*L*. *v*. *v*.		*L*. *v*.	*L*. *v*.	*L*. *(L*.*) v*.^6^	*L*. *v*.	*Lissotriton vulgaris*
			*T*. *(Pal*.*) v*. *schmidtlerorum*	*L*. *v*. *s*.						
	*Triton (Pal*.*) v*. *me*.			*L*. *v*. *me*.	*L*. *me*.				*L*. *meridionalis*	
	*Triton (Pal*.*) v*. *g*.			*L*. *v*. *g*.					*L*. *graecus*	
			*T*. *(Pal*.*) v*. *lantzi*	*L*. *v*. *lantzi*				*L*. *(L*.*) lantzi* ^6^	*L*. *lantzi*	
	*Triton (Pal*.*) Montandoni*		*T*. *(Pal*.*) mo*.	*L*. *mo*.		*L*. *mo*.	*L*. *mo*.	*L*. *(L*.*) mo*.	*L*. *mo*.	*L*. *montandoni*
	*Triton (Pal*.*) i*.		*T*. *(Pal*.*) i*.			*L*. *i*.	*L*. *i*.	*L*. *(L*.*) i*.	*L*. *i*.	*L*. *italicus*
*Triton h*.	*Triton (Pal*.*) palmatus*		*T*. *(Pal*.*) h*.	*L*. *h*.			*L*. *h*.	*L*. *(L*.*) h*.	*L*. *h*.	*L*. *helveticus*
	*Triton (Pal*.*) Boscài* ^7^		*T*. *(Pal*.*) boscai*	*L*. *b*.			*L*. *b*.	*L*. *(Meinus) b*.	*L*. *b*.	*L*. *boscai*
*Triton a*.	*Triton (Mes*.*) a*.^8^		*T*. *(T*.*) a*.	*Mesotriton a*. *a*.	*I*. *a*.	*I*. *a*.^9^	*I*. *a*.	*I*. *a*.	*I*. *a*.	*Ichthyosaura alpestris*
	*E*. *asper*	*Euproctus*	*E*. *asper*	*Ca*. *asper*	*Ca*. *asper*		*Ca*. *asper*	*Ca*. *asper*	*Ca*. *asper*	*Calotriton*
							*Ca*. *arnoldi*	*Ca*. *arnoldi*	*Ca*. *arnoldi*	
		*Neurergus*			*Ne*. *cr*.		*Ne*. *cr*.	*Ne*. *(Ne*.*) cr*.	*N*. *crocatus*	*Neurergus*
			*Ne*. *k*.	*Ne*. *k*.			*Ne*. *k*.	*Ne*. *(Ne*.*) k*.	*Ne*. *kaiseri*	
	*Triton (Mes*.*) crocatus* forma *m*.						*Ne*. *microspilotus*	*Ne*. *(Ne*.*) d*.	*Ne*. *derjugini*	
	*Triton (Mes*.*) crocatus* forma *Strauchi* (sic)		*Ne*. *strauchi* (sic [40]); *Ne*. *strauchii*	*Ne*. *strauchii*			*Ne*. *strauchii*	*Ne*. *(Musergus) strauchii*	*Ne*. *strauchii*	
		*Pachytriton*	*P*. *labiatum* (sic!; TL)^10^	*P*. *labiatus*			*P*. *labiatus*	*P*. *i*.	*P*. *inexpectatus*	*Pachytriton*
*S*.	*S*. *m*. *maculosa*	*Salamandra*	*S*. *s*. *s*.	*S*. *s*.	*S*. *s*.	*S*.	*S*. *s*.	*S*. *s*.	*S*. *salamandra*	*Salamandra*
							*S*. *i*.	*S*. *i*.	*S*. *infraimmaculata*	
		*Mertensiella*	*Mer*. *luschani a*.	*Ly*. *a*.			*Ly*. *a*.	*Ly*. *a*.	*Lyciasalamandra atifi*	excluded

^1^ It is not quite clear if the “groups” were meant to be subgenera (as e.g. AS later thought) or informal rankless taxa. [57] presented their names like those of subgenera (with parentheses), but failed to use the name *Triton (Triton)* for the “group” containing the type species (*T*. *cristatus*), opting for *Triton (Neotriton)* instead, and also used the term “group” for the genera other than *Triton*, which he did not subdivide.

^2^ WÖ nowhere indicated which species they actually used to score their OTUs, all of which are genera. For example, while they most likely used *Calotriton asper* to score “*Euproctus*”, it is not evident whether they considered *Euproctus montanus* or *E*. *platycephalus* at all (although the same authors had earlier [50] dissected *E*. *montanus* and occasionally cited literature on *E*. *platycephalus*). The same holds for SR, excepting the species-level OTUs.

^3^ Subgenera recognized only by AS.

^4^ Considered a hybrid population of *Triturus ivanbureschi* and *T*. *macedonicus*, therefore not recognized in nomenclature.

^5^ *Molge vulgaris vulgaris* in fig 35A.

^6^ Inconsistent treatment: the species page for *Lissotriton vulgaris* lists *L*. *v*. *lantzi* as one of several subspecies. *L*. *v*. *schmidtlerorum* of [78,83] is not mentioned anywhere in [84], in contrast to [85].

^7^ Lowercase *Triton boscài* on page 290, *Triton Boscai* without accent in the unnumbered figure on page 309.

^8^ Two subspecies: *Triton (Mesotriton) alpestris alpestris* and *Triton (Mesotriton) alpestris Reiseri*.

^9^ SR (page 61) explicitly preferred *Ichthyosaura* over *Mesotriton*, but two isolated occurrences of “*M*. *alpestris*” remain on pages 61 and 64.

^10^ To agree with the masculine gender of the genus name, the species name had to be *P*. *labiatus*.

[83] stated in Table 2 that foramina on the ventral surface of the atlas are “rare” in *Salamandra salamandra*, *Ichthyosaura alpestris*, *Lissotriton montandoni* and *L*. *vulgaris*, but “frequent” in three species of *Triturus (Triturus)* (see below) and in *Ommatotriton ophryticus*. Not only would this character be parsimony-informative if added to our matrix, but it would support the molecular topology [1,2] (Figs 1–3) according to which *Triturus* and *Ommatotriton* are more closely related to each other than to any of the other taxa mentioned above. Unfortunately, it is impossible to code this character from the illustrations in [40,83]; if we reinterpret “rare” and “frequent” as “few” and “many”, the scores even contradict the illustrations, a problem compounded by the fact that the line drawings in [40] are too simplified for some purposes. Thus, although many salamandrid species can be diagnosed from an isolated atlas, our matrix continues to lack any characters that describe atlas morphology. *Dicamptodon* and *Lissotriton italicus* are even scored as unknown for all characters of the postcranial skeleton; our inability to find published information on it is particularly surprising for *Dicamptodon*, because several extinct taxa known only from isolated vertebrae have been considered close relatives of *Dicamptodon* ([4]; partially accepted in [12]; rejected in [5]) by authors who had evidently seen *Dicamptodon* vertebrae but not published their observations of them.

Our taxon sampling was aimed at complete representation of Pleurodelinae as far as missing data allow it. All extant pleurodeline genera, as currently classified [2,84,85], are coded, except for *Laotriton* (sister-group of *Pachytriton*), the morphology of which is practically unknown to science. Of the non-pleurodeline salamandrids, we have retained *Salamandrina* and the salamandrines *Chioglossa* and *Salamandra*; we have excluded the salamandrines *Mertensiella* (sister-group to *Chioglossa* [1,2]), which is poorly known, and *Lyciasalamandra*, which is very similar to but generally somewhat more paedomorphic than its sister-group *Salamandra*. (Most mentions of *Mertensiella* in the literature refer to the only recently recognized *Lyciasalamandra*; see Table 2.)

To the taxon sample achieved by combining the source matrices, we added MB.Am.45, the Enspel and Randeck specimens of *Chelotriton* (as a single OTU, after [29,30]), *Brachycormus noachicus* (after [4,14,15]), and *“Triturus” roehrsi*, all of which have never before (to the best of our knowledge) been included in a phylogenetic analysis. Unfortunately, *C*. *robustus*, *Palaeopleurodeles* and “*Tylototriton*” *weigelti* are so poorly known that they would have scored identical to several other taxa in our matrix; this is not, however, the case for *“Triturus” roehrsi* (after [33,86]), which is known from isolated bones and has been variously referred to, in current terms (see Table 2), *Lissotriton* or *Ommatotriton* [4,33,86,87].

Thanks to the vertebral characters of [9], we were able to score? *Taricha miocenica*,? *Notophthalmus crassus*,? *N*. *robustus* and *Koalliella*, all known exclusively from isolated vertebrae of Paleocene to Miocene age (after [4]). Contrary to expectations, all of them score differently from all other OTUs in this matrix.

We originally included? *Taricha oligocenica* based on DM’s observations of casts; we have removed it from our matrix because it is being redescribed [88].

Given that [3,60] used individual species of *Lissotriton* as OTUs, we further added the extant *Lissotriton boscai* (after [40,57,89]) as an OTU to our matrix. We have not been able to find useful information on the remaining two extant species that are recognized by [84,85], but were traditionally considered subspecies of *L*. *vulgaris* (*L*. *kosswigi*, *L*. *lantzi*).

[60] included *Triturus marmoratus* and *T*. *cristatus* as OTUs, [3] used an undifferentiated *Triturus* OTU, [52] sampled only *T*. *karelinii* (their “*T*. *alpestris*” is *Ichthyosaura alpestris*), and [51] used a “*Triturus*” OTU that must have included *Ichthyosaura*, *Ommatotriton* and *Lissotriton* as was usual at the time. We have kept *T*. *marmoratus* as an OTU, but use the whole subgenus *T*. *(Triturus)* as another: *T*. *cristatus*, *T*. *karelinii* + *T*. *ivanbureschi*, *T*. *carnifex* and *T*. *dobrogicus* are so closely related that [2] (supplementary file amph_shl.tre) and [90] (S1 Fig) had some trouble resolving their mutual relationships and came to contradictory results; [57] found the skulls of *T*. *carnifex*, *T*. *ivanbureschi* and *T*. *karelinii*, as well as those of *T*. *cristatus* and *T*. *dobrogicus*, to be identical, whereas [90] found only *T*. *cristatus* and *T*. *dobrogicus* to be sister-groups and [2], who did not sample *T*. *ivanbureschi*, found only *T*. *carnifex* and *T*. *karelinii* to be sister-groups.–Our use of the name *T*. *(T*.*)* follows [84]. We avoid the term “*T*. *cristatus* group”, which historically referred to a much larger assemblage that contained all of *Triturus* and *Ommatotriton* (e.g. [40]: 58). Conversely, [91] used the term “*T*. *karelinii* group” for *T*. *karelinii* and *T*. *ivanbureschi*, a small part of *T*. *(T*.*)*.–*T*. *marmoratus* forms the subgenus *T*. *(Pyronicia)* with *T*. *pygmaeus*, about which we have not been able to find anatomical information; we have therefore kept *T*. *marmoratus* alone as an OTU.

For several reasons, [14] (page 486) considered it “possible that the known specimens of *Brachycormus noachicus* represent more than one species.” Most of the reasons were not actually stated, but our *Brachycormus* OTU is indeed polymorphic for the width/length ratio of the skull (character 7). We have nonetheless included it in our analysis in order to test the hypothesis of a close relationship of *Brachycormus* to *Chelotriton*, *Tylototriton* and *Echinotriton*, proposed in [4] and supported in [15] after rejection in favor of molgin affinities in [14].

As outgroups, we used *Ambystoma* and *Dicamptodon*, following [52]; more recent phylogenetic analyses of Urodela ([2] and references therein) confirm that a clade formed by *Ambystoma* and *Dicamptodon* is the sister-group of Salamandridae. [51] did not use an outgroup at all, but instead polarized their characters a priori; [3] similarly (see [92]) used a hypothetical all-zero ancestor. [58] used *Salamandra* as the outgroup for an analysis where Pleurodelinae alone was the ingroup; they excluded *Salamandrina* which [3,50] had found to be the sister-group of Pleurodelinae, while molecular analyses [1,2] (Figs 1–3) have found *Salamandrina* to be the sister-group of a Salamandrinae + Pleurodelinae clade with moderate support. This molecular result was accepted a priori in [9], where *Salamandrina* was used as the outgroup for an analysis which only included salamandrids. [61] only analysed a part of Pleurodelinae and therefore used other pleurodelines as the outgroup.

Unfortunately, we are unable to add the only Asian fossil salamandrid, the Miocene *Procynops*, to our matrix; it is so poorly known [93] that it would score indistinguishably from several other OTUs.

Similarly, the preservation and skeletally immature (neotenic) status of the Paleocene or Eocene *Seminobatrachus* have prevented us from including this possible sister-group of *Ambystoma* [94] in our analysis.

MB.Am.45 and the *Chelotriton* specimens from Enspel and the Randeck Maar score identically in our matrix except for the distribution of missing data. We have nonetheless kept them as two separate OTUs in order to test whether they have synapomorphies. If this is the case, they should be sister-groups in the resulting trees; if not, they should form a trichotomy with their closest relative (irrespective of whether that is an OTU or a larger clade).

## Results and Discussion

### Heterochrony in MB.Am.45

MB.Am.45 shows a mixture of peramorphic and paedomorphic characters in its skeleton. A peramorphic feature is the generally high degree of ossification in the skeleton. This applies especially to the massive, hyperossified skull in which the maxilla is sutured posteriorly with the quadratojugal-quadrate, and the supratemporal opening is much reduced in size. The frontosquamosal bar is proportionally conspicuously broadened compared to other salamandrids, including most or all specimens currently referred to *Chelotriton*. Associated with the heavily ossified skull is the pronounced dermal sculpture that can be found on each of the bones of the dermal skull roof. The dermal sculpture of the maxilla closely resembles that of early limbed vertebrates like temnospondyls or lepospondyls [42], which in turn show a generally much higher degree of ossification in the dermal skeleton than lissamphibians do. Lissamphibians are widely thought to be paedomorphic (in particular progenetic) with respect to their Paleozoic ancestors ([95–97]; more cautiously [98]: 238; salientians: [13,99,100] and references therein). During caudate ontogeny, the position of the jaw articulation moves posteriorly [101–103], but its adult position is normally not posterior to the occiput; it remains well anterior to it in most cases, reaches about the same level in a few others, and comes to lie just barely posterior to the occiput only in *Dicamptodon* [104] and maybe some specimens of *Chelotriton* [24]. The position of the jaw articulation far behind the occiput in MB.Am.45 is thus an extension of “normal” caudate ontogeny and can be regarded as a peramorphic trait. It resembles the situation in many temnospondyls, in which the jaw joint moves far posteriorly during ontogeny (e.g. [105,106], as well as other Paleozoic tetrapods like seymouriamorphs, where a similar allometry of lesser extent is found (e.g. [107]: figs 7, 8).

The possible presence of a septomaxilla in a salamandrid is likewise of interest with respect to heterochrony. This bone–when present–is one of the last bones of the dermal skull roof to ossify in caudates ([41] and references therein). Its general absence in salamandrids might therefore represent a paedomorphic trait, and its reappearance in MB.Am.45, if correctly identified, could then be interpreted as peramorphic.

The short, blunt, triangular, widely separated alary processes of the premaxillae might be a further peramorphic feature: they are found in the metamorphosed *Aviturus* but not in the other (incompletely metamorphosing) cryptobranchids, which have more medially positioned, longer and more pointed dorsomedial processes as usual in salamanders [108]. MB.Am.45 and *Aviturus* share this feature with almost all temnospondyls.

The resemblance of MB.Am.45 with early limbed vertebrates is further increased by the presence of long, curved ribs with tubercular processes, which resemble the so-called uncinate processes of many temnospondyls–although there are at most two such processes on temnospondyl ribs, and they point caudally, not dorsally. In most caudates, the ribs are very short and (nearly) straight, and even the elongate ribs of many pleurodelins are proportionally shorter than those of MB.Am.45. Finally, MB.Am.45 has an ossified olecranon process on the ulna, unlike even the Enspel and Randeck specimens of *Chelotriton* [30].

In contrast to these features which we interpret as peramorphic, the presence of a larval hyobranchial apparatus with ossified ceratobranchials (epibranchials of some authors) is clearly a paedomorphic feature. Furthermore, this character suggests a primarily aquatic mode of life, in spite of the highly ossified skeleton. In the combination of a paedomorphic hyobranchium with an otherwise highly ossified skeleton, MB.Am.45 resembles *Brachycormus noachicus*, which was interpreted as a “neotenous larva” in [13–15]. However, the proportions of the skull of *Brachycormus*, especially the anteriorly located jaw articulation, appear more “larval” than those of MB.Am.45. In contrast to *Brachycormus* and MB.Am.45, other specimens traditionally referred to *Chelotriton paradoxus* have been described as having a completely metamorphosed hyobranchial skeleton that was not affected by paedomorphosis [13–15]; however, it has more recently been shown [29,30] that the Enspel and Randeck specimens of *Chelotriton* had an unmetamorphosed hyobranchium containing an ossified hypobranchial and two ossified ceratobranchials on each side. Paedomorphosis and an aquatic lifestyle are seen in many late Oligocene caudates; this has been connected [13,15,29] to the fact that the climate cooled markedly during that time.

Interestingly, the combination of a generally peramorphic skeleton (jaw joints and occiput at the same mediolateral level) with a larval hyobranchial apparatus has a precedent in the Jurassic cryptobranchoid [44] *Pangerpeton* [38].

To summarize, although it possesses a paedomorphic hyobranchium, MB.Am.45 is peramorphic in many aspects of its skeleton and superficially resembles an amphibamid temnospondyl or (to a lesser extent) a diplocaulid “lepospondyl”. However, this resemblance is superficial. The long ribs with processes certainly served different functional roles in MB.Am.45 and temnospondyls; whereas the processes and flanges on the ribs of early limbed vertebrates are directed more or less caudally and probably served for postural support [109], the dorsally directed processes of ribs in extant pleurodelins support (and sometimes penetrate) overlying warts in the skin, as do the sharp tips of these ribs [8]. Despite the heavy ossification and the peramorphic proportions of the skull, there is no reappearance of “ancient” bones in the skull of MB.Am.45, in contrast to the additional bones in pelobatid anurans investigated in [99] and interpreted there–on little evidence–as e.g. postparietals and supratemporals.

### Affinities of MB.Am.45 and the diversity of *Chelotriton*

Although MB.Am.45 is clearly referrable to *Chelotriton* (see Material and Methods – Systematic Paleontology), we cannot assign it to a species as long as *Chelotriton* has not been revised. Its referral to *C*. *paradoxus* in [29], for which no reasons were given, seems to have been based only on the pustular sculpture on most bone surfaces that were in contact with the skin, a character state shared by *C*. *paradoxus*, *C*. *pliocenicus* and all other possible species of *Chelotriton* except *C*. *robustus* (where all sculpture is more or less honeycombed [17]), and on the fact that other specimens from the same and similar ages and localities had already been referred to *C*. *paradoxus*.

MB.Am.45 differs from *Chelotriton paradoxus* from the neotype locality [39] in having frontals that are broader than long. This is shared with some specimens from Enspel ([29]: fig 2a_5_) but not others ([29]: fig 2b_1_; [30]: figs 2, 3c). In *C*. *paradoxus* from the neotype locality, the angle between the squamosal process of the frontal and the sagittal plane is 30°–35° [39]; in MB.Am.45, the process is so wide that it cannot easily be said to lie at a particular angle to the sagittal plane, but if the midpoint of a straight line from the medial corner of the prefrontal to the rostromedial corner of the supratemporal fenestra is connected to the midpoint of the frontal/squamosal suture, an angle of at least 45°, probably closer to 50°, results. Most likely, then, MB.Am.45 does not belong to *C*. *paradoxus*.

The supratemporal fenestrae are completely surrounded by sculptured bone, or nearly so: an unsculptured trough for jaw-closing muscles between the medial and the lateral sculptured surfaces of the right parietal could only have been present in the crack caudal to the medial margin of the postorbital opening, along which the lateral sculptured surface of the parietal has been raised about 1 mm above the medial part by diagenetic compression and the lateral margin of the medial sculptured surface of the right parietal has been bent upwards (Fig 11). This extreme narrowness or absence of the unsculptured trough constitutes a further difference to *C*. *paradoxus* from the neotype locality as well as to all other comparable *Chelotriton* material except possibly “*Tylototriton primigenius*” Noble, 1928, which is currently referred to *C*. *paradoxus*.

The vertebrae of MB.Am.45 –about the first ten are preserved–are not well enough visible to determine the presence (as in *C*. *pliocenicus*) or absence (retained in *C*. *paradoxus*) of zygosphenes and zygantra. Isolated ribs from the early Miocene site of Petersbuch (Germany) referred to “*C*. aff. *pliocenicus*” [19] have up to ten dorsal spines (epipleural processes); the preservation of MB.Am.45 does not allow a clear statement on whether more than five spines (Figs 5 and 13) were present on some ribs.

MB.Am.45 is distinctly smaller but more peramorphic than late Oligocene *Chelotriton* specimens from the fairly close site of Enspel ([29]: fig 2; [30]: figs 2, 3c), called “*Chelotriton*-like forms” on page 12 of [19]; in particular, the quadratojugal-quadrate bones project far caudally beyond the squamosals, the postorbital openings are completely surrounded by sculptured bone (or nearly so), and there might even be a septomaxilla. Unless MB.Am.45 is–we might speculate–a hormonally abnormal individual with greatly accelerated development, it therefore likely represents a different species under most or all applicable species concepts, contrary to its referral to the same species–*C*. *paradoxus*–in [29]. The Enspel and Randeck specimens also have unusually short snouts in which the suture between the nasals is only about half as long as that between the frontals ([30]: figs 1, 2, 3c, 3d); in MB.Am.45 the snout is less extremely blunt, and the frontals are only a little longer than the nasals. In principle, this could be an ontogenetic difference; but the extant *Echinotriton* and *Tylototriton*, generally less peramorphic than the Enspel and Randeck specimens, have intermediate proportions ([30]: figs 3a, 3b).

MB.Am.45 differs from both “*Chelotriton* sp., type I” and “*Chelotriton* sp., type II” [13,32] in that the dermal sculpture, where it is tuberculate at all, consists of lower tubercles that are more individualized; between them we cannot find any foramina (as in both “type I” and “type II”), but instead a flat, smooth surface is often visible. As in both “types”, the tubercles on the frontosquamosal arch are more or less connected to form parallel ridges; however, MB.Am.45 differs from “type I” in that the sculpture on the maxilla is honeycombed as in *C*. *robustus* (the maxilla of “type II” is unknown). This honeycombed sculpture is approximated by the isolated caudal ramus of a maxilla from Götzendorf (late Miocene, Austria) that was referred to *C*. *paradoxus* in plate 2I of [27].

MB.Am.45 further differs from “aff. *Chelotriton* sp.” from Oberleichtersbach (latest Oligocene, Germany) [39] in having pustular rather than vermicular or pit-and-ridge sculpture on the frontals and in having large, well-developed spine tables on the vertebrae. This character state is shared with most of the other material currently referred to *Chelotriton*.

Ribs from Sandelzhausen (latest early Miocene, Germany) referred to “*Chelotriton* sp.” [19] have only one spine, while MB.Am.45 shows at least five on some (Figs 5 and 13). *C*. *paradoxus* from the neotype locality has two to four spines per rib [19,39].

MB.Am.45 differs from all comparable *Chelotriton* material by its wide skull (ratio of width to midline length 1.94, compared to a maximum of 1.55 elsewhere in *Chelotriton*). It differs from all other caudates except *Dicamptodon*, *Salamandra “laticeps”* and maybe a few *Chelotriton* specimens by the fact that its jaw joints lie caudal to the occiput, and from all other caudates (and most other lissamphibians) in the extreme extent of this feature, particularly the caudal projection of the quadratojugal-quadrate bones beyond the squamosals. Correspondingly, the squamosals are inclined rostromedially to caudolaterally. The frontosquamosal arch (a pleurodeline autapomorphy) is proportionally much wider in MB.Am.45 than in any other pleurodeline, and so is the reconstructed interorbital width; correspondingly, the squarish postorbital opening between this arch and the skull table is proportionally smaller than in any other pleurodeline. Unlike in all other salamandrids, including the *Chelotriton* specimens from Enspel and the Randeck Maar [30], a possible septomaxilla is present. All these features are indicative of peramorphosis; although such peramorphosis may well be an autapomorphy of a hitherto unknown species, MB.Am.45 is difficult to compare with some of the material referred to *Chelotriton*, and the ontogeny of *Chelotriton* is hardly known.

Like other skeletons referred to *Chelotriton* [29,30], MB.Am.45 preserves a hyobranchial apparatus of aquatic type. The zygosphene-zygantrum articulations of *C*. *pliocenicus* have been interpreted as a terrestrial feature, because they must have stiffened the vertebral column against torsion [18]; if MB.Am.45 was aquatic (as indicated by its hyobranchium) and *C*. *pliocenicus* was terrestrial (as suggested by its zygosphenes and zygantra), they were most likely not conspecific, but unfortunately the material is not comparable in any other way to the best of our knowledge.

*C*. *robustus* has honeycombed sculpture on all bones that contacted the skin; in all other material hitherto referred to *Chelotriton*, except the ridged, almost honeycombed incomplete maxilla from Götzendorf ([27]: plate 2I), all sculpture is pustular. MB.Am.45 has pustular sculpture on most bones that were exposed to the dermis, but the maxillae and possibly the premaxillae have a honeycombed surface. It is uncertain whether this feature has ontogenetic significance; the honeycombed vertebrae of *C*. *robustus* are much smaller than pustular vertebrae referred to *C*. *paradoxus* ([17]: 484).

### Salamandrid phylogeny

MB.Am.45 and the *Chelotriton* specimens from Enspel and the Randeck Maar are found to be sister-groups in all analyses. They share the following synapomorphies that are absent in *Tylototriton*, *Echinotriton* and *Pleurodeles* (numbers of characters and states from the Appendix):

20(3): Ratio of interorbital width to skull length 0.5 or higher. Also found in *Brachycormus*.

37(0): Jaw articulation near posterior margin of parasphenoid or caudal to it. Also found in *Dicamptodon*, *Salamandra* and *Euproctus*. (The plesiomorphic state of this character is 1.)

69(2): Diapophyses or parapophyses extend laterally beyond a line along the lateral edges of the zygapophyses by > 22.5% of the maximum width between any zygapophyses. Also found in some *Ambystoma* species, *Paramesotriton*, *Cynops*, *“Triturus” roehrsi* and *Carpathotriton*.

#### Unconstrained analysis without geographic distribution as a character

**Fig 15:**
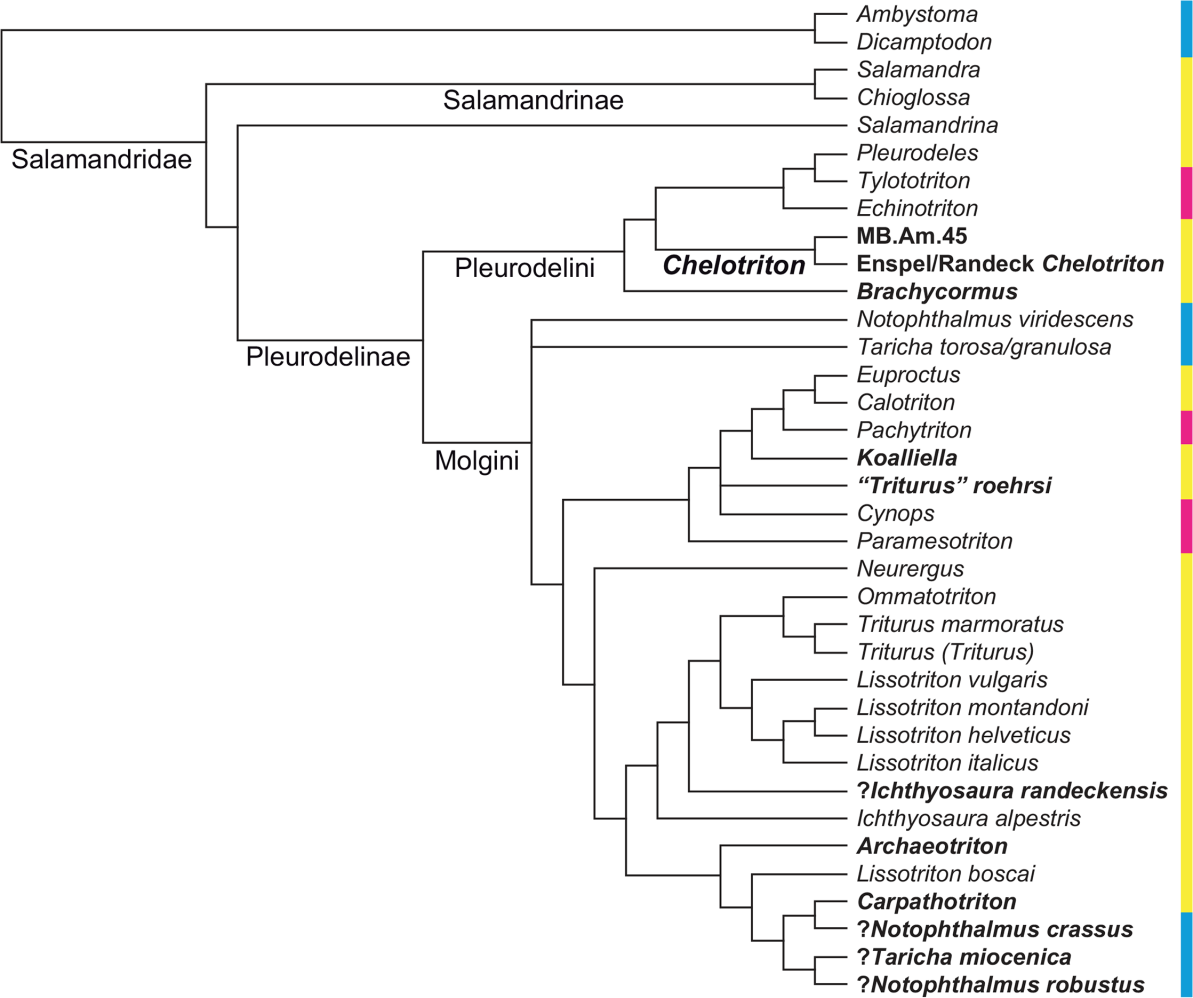
Identical strict and Adams consensus of the most parsimonious trees from our unconstrained analysis without geographic distribution as an added character (length of individual trees = 595 steps). Names of extinct taxa are in boldface. Colors as in Fig 1.

Despite the large amounts of missing data, our simplest analysis resulted in only six most parsimonious trees (length = 595 steps, including steps within polymorphic OTUs; consistency index = 0.4992; homoplasy index = 0.7580; retention index = 0.6042; rescaled consistency index = 0.3016). Their strict consensus (Fig 15) is correspondingly well resolved: polytomies are absent except that in individual trees either *Notophthalmus* or *Taricha* (see below) is the sister-group to all other molgins, and *“Triturus” roehrsi* occurs in all three positions implied by the trichotomy it is shown in.

The identical strict and Adams consensus (Fig 15) mixes familiar and unfamiliar results. The outgroup (*Ambystoma* + *Dicamptodon*) and Salamandridae are mutually monophyletic; within the latter, Salamandrinae (*Salamandra* + *Chioglossa*) and Pleurodelinae as currently understood are monophyletic to the exclusion of each other and of *Salamandrina*. *Salamandrina* emerges as the sister-group of Pleurodelinae; this is a long-standing morphology-based hypothesis, but contradicts recent molecular analyses, where *Salamandrina* was found to be the sister-group to all other salamandrids together with rather high [1] to low support ([2]: supplementary file amph_shl.tre). Given the low support in the latter analysis (Fig 2), the imperfect support in the former (Fig 1), and such morphological characters as the presence of the frontosquamosal arch in *Salamandrina* and Pleurodelinae but absence without a trace in even the largest, most peramorphic salamandrines [4,21], we would like to suggest the phylogenetic position of *Salamandrina* as a topic for future research.

The outgroup was not constrained to be monophyletic. The PAUP* setting “outroot = monophyl”, which we used (S1 Data), means that the unrooted tree resulting from the analysis will be rooted so that the outgroup forms a clade if that is possible given the topology of the unrooted tree; in a few early runs of our analysis (before further corrections to the matrix), this was not possible because *Dicamptodon* came out as the sister-group of Pleurodelinae–PAUP* only issued the warning that the “tree cannot be rooted so that outgroup is monophyletic” and rooted the tree on *Ambystoma* alone (*Ambystoma* being OTU number one). Thus, the fact that *Ambystoma* and *Dicamptodon* appear as sister-groups in Fig 15 is a result of our analysis, independently confirmed by analyses of molecular data (most recently [2]; see Fig 2), not an assumption we made a priori.

Pleurodelinae consists of the long-recognized clades Pleurodelini and Molgini. Strangely, within Pleurodelini, *Tylototriton* and *Pleurodeles* are sister-groups, together forming the sister-group of *Echinotriton*; outside this pleurodelin crown-group, MB.Am.45 is the sister-group of the Enspel and Randeck specimens referred to *Chelotriton*, and *Brachycormus* is the sister-group to all other total-group pleurodelins.

Within Molgini, *Taricha* and *Notophthalmus* are generally thought to be sister-groups [57], forming a North American clade that is the sister-group to the Eurasian molgins [1,2]. Their skeletons and courtship behaviors are sufficiently different from each other’s that none of our six shortest trees contains this topology; instead, in some of them the extant *Notophthalmus* is closer to the Eurasian molgins than to the extant *Taricha* species, while in the others they switch places. Given the strong support for the North American clade in molecular analyses, we hypothesize that our result is due to a lack of characters; *Taricha* and *Notophthalmus* likely split from each other a long time ago and have evolved numerous autapomorphies, some of which are reversals while others are convergent to the Eurasian clade. The fossil record, first and foremost the upcoming redescription of? *Taricha oligocenica* (see [88]), might clarify this in the future; however, we find the extinct? *Taricha miocenica*,? *Notophthalmus crassus* and? *N*. *robustus*, known from isolated vertebrae only, to lie far away from the extant *Notophthalmus* and *Taricha* species, within the Old World molgin clade, forming an unexpected clade with the extinct *Carpathotriton*, the extant *Lissotriton boscai* and the extinct *Archaeotriton*.

*Archaeotriton*, like *Carpathotriton*, has only once before been included in a phylogenetic analysis, where it was found to be the sister-group of *Salamandrina* [3]; our matrix contains all taxa and all characters of [3], so we consider our analysis to supersede the one in [3]. One factor in the discrepancy may be that [3] accepted the misidentification in [16] of the otic capsule as the squamosal (see character 27 in the Appendix), but did not extend this untenable hypothesis of homology to the other taxa in the matrix. Our result instead confirms the earlier [16] hypothesis that *Archaeotriton* is a pleurodeline and specifically a molgin.

Within the Old World clade of molgins, an Asian clade is generally thought to exist, comprising *Cynops*, *Paramesotriton*, *Pachytriton* and *Laotriton*, the last of which we were unable to include in our analysis due to the almost complete lack of nonmolecular data. Strong support for this clade was found in [1,2]. We find such a clade, but nested within it are European forms: the extant mountain-stream newts *Euproctus* and *Calotriton*, the poorly known Miocene *“Triturus” roehrsi*, and the Paleocene *Koalliella* which is only known from (drawings and descriptions of) isolated vertebrae.

*Euproctus* and *Calotriton* are so similar to each other and so distinct from other newts morphologically, as well as in courtship behavior, that they were considered the same genus (*Euproctus*) for well over a century (e.g. [52,57]) and were only separated again by molecular data [1,2,66]. [66] ascribed this strong morphological and behavioral similarity to convergent adaptations for a life in mountain streams. Given the compelling molecular support for a sister-group relationship of *Calotriton* and *Triturus* far removed from *Euproctus* [1,2,66], which further agrees with biogeographic considerations–*Calotriton* and *Triturus* occur next to each other near the likely center of origin of *Triturus* [90]; *Euproctus* is restricted to Corsica and Sardinia, which have been isolated from the mainland for a long time and where other pleurodelines are absent–, we are well inclined to agree, even though we have tried to identify correlated characters and to avoid scoring them separately (see Appendix).

The extant *Pachytriton* is not found as the sister-group to the extant *Paramesotriton*, but to (*Euproctus* + *Calotriton*). Although less extremely adapted to a life in mountain streams, *Pachytriton* shows adaptations to a fully aquatic existence in rather small bodies of mostly running water beyond those generally seen in *Cynops* and *Paramesotriton*; this may explain its unexpected position in our phylogeny. The intriguing fact that *Koalliella* forms the sister-group to this clade in our trees is unlikely to be correct as a phylogenetic result, given its Paleocene age and European location; however, it could indicate a similar lifestyle. We are not aware of any previous hypotheses on the ecology of *Koalliella* or any previous comparisons to *Pachytriton*.–To a lesser degree, such a hypothesis may also hold for *“Triturus” roehrsi*.

The sister-group to this “Asian” clade is a “European” clade which also contains the isolated North American vertebrae as mentioned. The sister-group to the rest is *Neurergus*, conforming to its traditional position outside a large “*Triturus*” grouping but strongly contradicting molecular analyses [1,2], which found an *Ommatotriton*-*Neurergus* clade to be sister to a *Calotriton*-*Triturus* clade with strong to very strong support, all of them together forming either the sister-group to a *Lissotriton*-*Ichthyosaura* clade (strong support: [1]; Fig 1) or to *Lissotriton* alone (less strong support: [2]; Fig 2). Within the rest of the “European” clade, a clade similar to traditional “*Triturus*”, there is a dichotomy between the abovementioned clade of North American and European fossils and *Lissotriton boscai* on the one hand and all remaining newts on the other hand. The basal dichotomy in the latter clade separates the remaining species of *Lissotriton* from the other extant genera (*Ichthyosaura*, *Ommatotriton*, *Triturus*).

The description of the Miocene? *Ichthyosaura randeckensis* [60] identified two character states–spike-like tips of the dorsomedial processes of the premaxillae, and a frontal/nasal suture that is not straight but accommodates a rostrolateral process of the frontal which wedges between the nasal and the prefrontal–as uniquely shared by? *I*. *randeckensis* and the extant *I*. *alpestris*, and used them as their emended diagnosis of *Ichthyosaura*. While both of these characters remain parsimony-informative in our matrix, pointed (as opposed to blunt) dorsomedial processes of the premaxillae also occur in *Ommatotriton*, *Lissotriton vulgaris*, *Cynops*, *Notophthalmus*, and parts of *L*. *helveticus* and *Taricha* (see character 13 in the Appendix); a nasal/frontal suture of the described shape is also found in *Ommatotriton*, throughout *Triturus*, in *L*. *italicus*, *Euproctus*, *Cynops*, *Notophthalmus* and *Pleurodeles* as well as parts of *Pachytriton* (see character 17 in the Appendix). We find? *I*. *randeckensis* to be the sister-group of a clade composed of the remaining *Lissotriton* species, *Ommatotriton* and *Triturus*, with *I*. *alpestris* forming the sister-group to all of the above. However, nomenclatural consequences would likely be premature, because the position of *Neurergus* does not agree with the well-supported one as the sister-group of *Ommatotriton* found in [1,2]. Here, too, we would like to encourage further research.

The remaining species of *Lissotriton* form a clade with an unusual topology where *L*. *vulgaris* is the sister-group to the others. *Ommatotriton* comes out as the sister-group of a monophyletic *Triturus*.

Comparison to the molecular consensus (Fig 3) strongly suggests that it may not so much be ontogeny that “discombobulates phylogeny” ([110]: title) in salamandrids–adaptations to a life in mountain streams appear to have this effect most strongly.

#### Unconstrained analysis with geographic distribution included as a character

**Fig 16:**
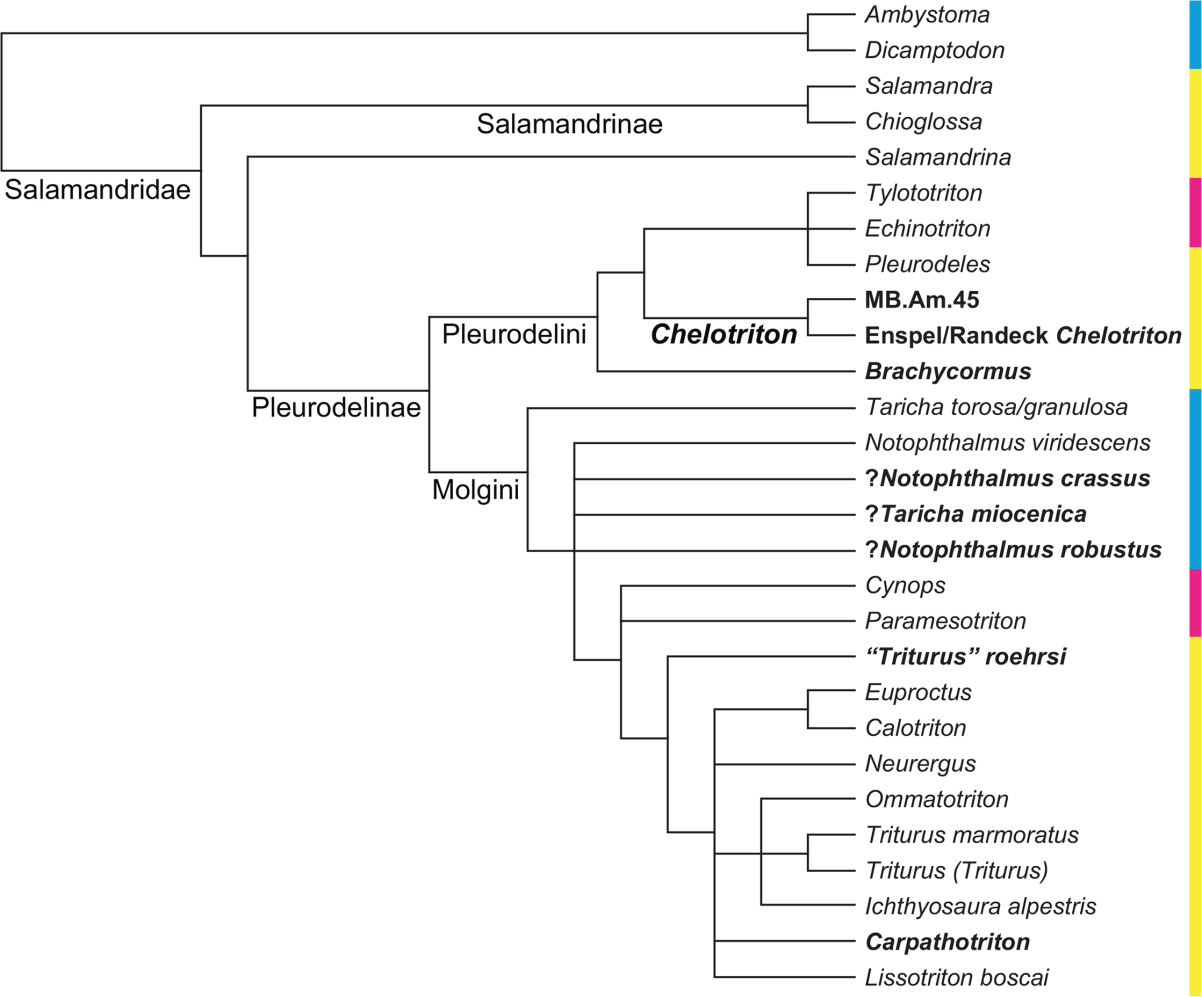
Strict consensus of the pruned most parsimonious trees from our unconstrained analysis that includes geographic distribution as character 98 (length of individual trees before pruning = 603 steps). *Archaeotriton*, *Koalliella*,? *Ichthyosaura randeckensis*, *Pachytriton*, *Lissotriton vulgaris*, *L*. *montandoni*, *L*. *italicus* and *L*. *helveticus* were pruned from all trees before calculating their strict consensus, but were included in the phylogenetic analysis that generated the trees. Names of extinct taxa are in boldface. Colors as in Fig 1.

Adding geographic distribution–the unordered character 98: North America (0); Europe, Mediterranean, Iran (1); eastern Asia (2); see Appendix –to the matrix increased the number of most parsimonious trees to 1067 (length = 603 steps, including steps within polymorphic OTUs; consistency index = 0.4959; homoplasy index = 0.7579; retention index = 0.6016; rescaled consistency index = 0.2983). The strict consensus of so many trees is, naturally, very poorly resolved, with the exception of the basalmost nodes and Pleurodelini, within which MB.Am.45 remains the sister-group to the OTU formed from the Enspel and Randeck specimens of *Chelotriton* while *Brachycormus* forms the sister-group to the rest of Pleurodelini (S4 Fig). The better resolved Adams consensus (S5 Fig) and inspections of individual trees show that the extinct *Archaeotriton*, *Koalliella* and? *Ichthyosaura randeckensis*, but also the extant *Pachytriton* and all *Lissotriton* species except *L*. *boscai* each have several very different optimal positions. Pruning them all from the most parsimonious trees results in a strict consensus that shows much more structure (Fig 16). This consensus tree has the extant *Taricha* species as the sister-group to all other molgins; that clade consists of a polytomy formed by the other American species (extant and extinct) and a Eurasian clade, which in turn has the Asian *Cynops* and *Paramesotriton* in a trichotomy with a European clade.

Outside the crown-group of this European molgin clade we find *“Triturus” roehrsi*; it belongs neither to *Lissotriton* ([4] and reference therein; [27,86]) nor to *Ommatotriton* [33,87]. This result highlights the fact that large amounts of missing data (*“T*.*” roehrsi* is only known from isolated bones) do not necessarily cause poor resolution, and it underscores the importance of phylogenetic analysis in determining whether supposedly diagnostic characters are synapomorphies.–If this result is correct, nomenclatural consequences will be required if genera are to be monophyletic. We refrain from drawing those (the most obvious possibility would be a new genus name for *“T*.*” roehrsi*) because we have not seen relevant material, because our analysis does not find the Asian clade that is so strongly supported by molecular analyses, because the position of *Ichthyosaura* remains an open question on which [1] and [2] disagreed with fairly strong support (see below and Fig 3), and because the sources cited above hint at the existence of vertebral characters which we have not been able to sample; however, we strongly encourage further research on this taxon that might occupy an important position in newt evolution.

The crown-group of the European molgin clade is poorly resolved; it contains the extinct *Carpathotriton* as well as the extant *Neurergus*, a clade formed by the extant *Euproctus* and *Calotriton*, and another formed by *Ichthyosaura*, *Triturus* and *Ommatotriton*.

Our addition of character 98 appears to have counteracted the ecological signal that produced a most likely spurious clade of aquatic Eurasian molgins; unfortunately, this only led to a balance with other signals and therefore to a drastic loss of resolution.

#### Analysis constrained to fit the molecular consensus, lacking geographic distribution as a character

**Fig 17:**
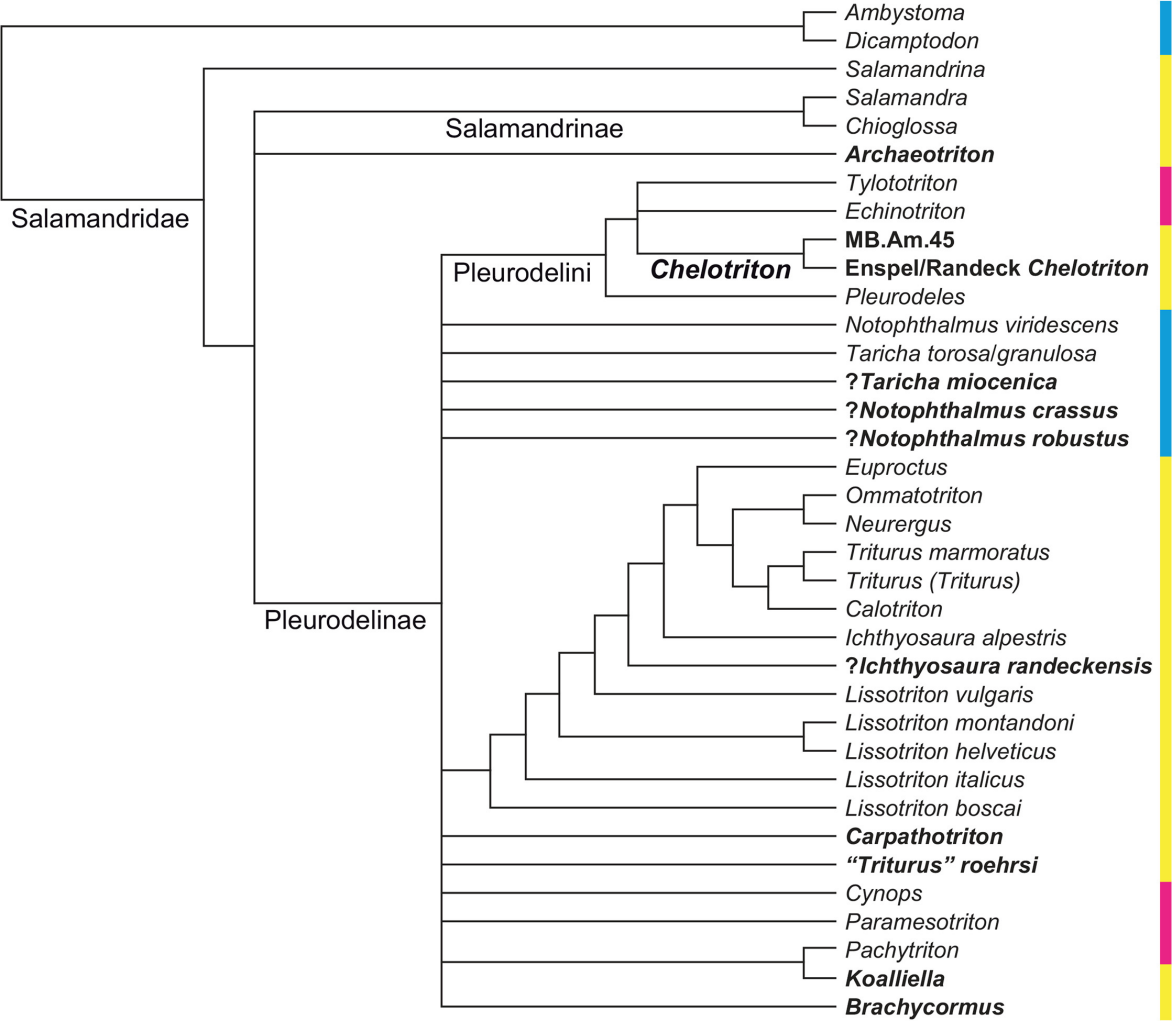
Strict consensus of the most parsimonious trees from our constrained analysis without character 98 (length of individual trees = 627 steps). Names of extinct taxa are in boldface. Colors as in Fig 1.

In this and the following analysis, we used the strict consensus tree of [1] and [2], reduced to the taxa they share with each other and with our matrix (Fig 3), as a backbone constraint. When character 98 is excluded from the analysis, 89 trees result, which require 32 more steps than the unconstrained analysis (length = 627 steps, including steps within polymorphic OTUs; consistency index = 0.4737; homoplasy index = 0.7703; retention index = 0.5618; rescaled consistency index = 0.2661); their strict consensus (Fig 17) is much less well resolved than the consensus of the corresponding unconstrained trees (Fig 15). As in the unconstrained analyses, MB.Am.45 and the Enspel/Randeck specimens of *Chelotriton* are sister-groups and are nested within Pleurodelini, and *“Triturus” roehrsi* stays far away from *Ommatotriton* and at least out of *Lissotriton*. Perhaps surprisingly, *Lissotriton* forms a paraphyletic series of relatives to the other European molgins, within which? *Ichthyosaura randeckensis* forms the sister-group to the rest. (The monophyly of *Lissotriton* was not enforced because [1] only included *L*. *vulgaris* in their analysis; [2] found strong support for the monophyly of *Lissotriton*, see Fig 2.)

Intriguingly, *Koalliella* is found to be the sister-group of *Pachytriton* in all shortest trees. This is similar to the result from the unconstrained analysis without character 98, and may be attributable to the same ecological signal.

Far from being close to *Lissotriton boscai* as in the unconstrained analysis without character 98 (Fig 15), *Archaeotriton* forms a trichotomy with Pleurodelinae and Salamandrinae in the strict consensus of the shortest trees from the constrained analysis without character 98 (Fig 17). Inspection of all those trees shows that *Archaeotriton* either lies as rootward on the stem of Pleurodelinae as possible in some trees and even on the stem of Salamandrinae in the others; *Brachycormus* is on the stem of Molgini in some trees, on that of Pleurodelinae (as the sister-group to the crown, thus crownward of *Archaeotriton* in trees where both are pleurodelines) in the others; *Carpathotriton*, if we ignore the shifting positions of the fragmentary taxa mentioned below, is either the sister-group to the European molgin clade or on the pleurodeline stem (between *Archaeotriton* and *Brachycormus*)–a position we would rather have expected for the much older *Koalliella*.

**Fig 18:**
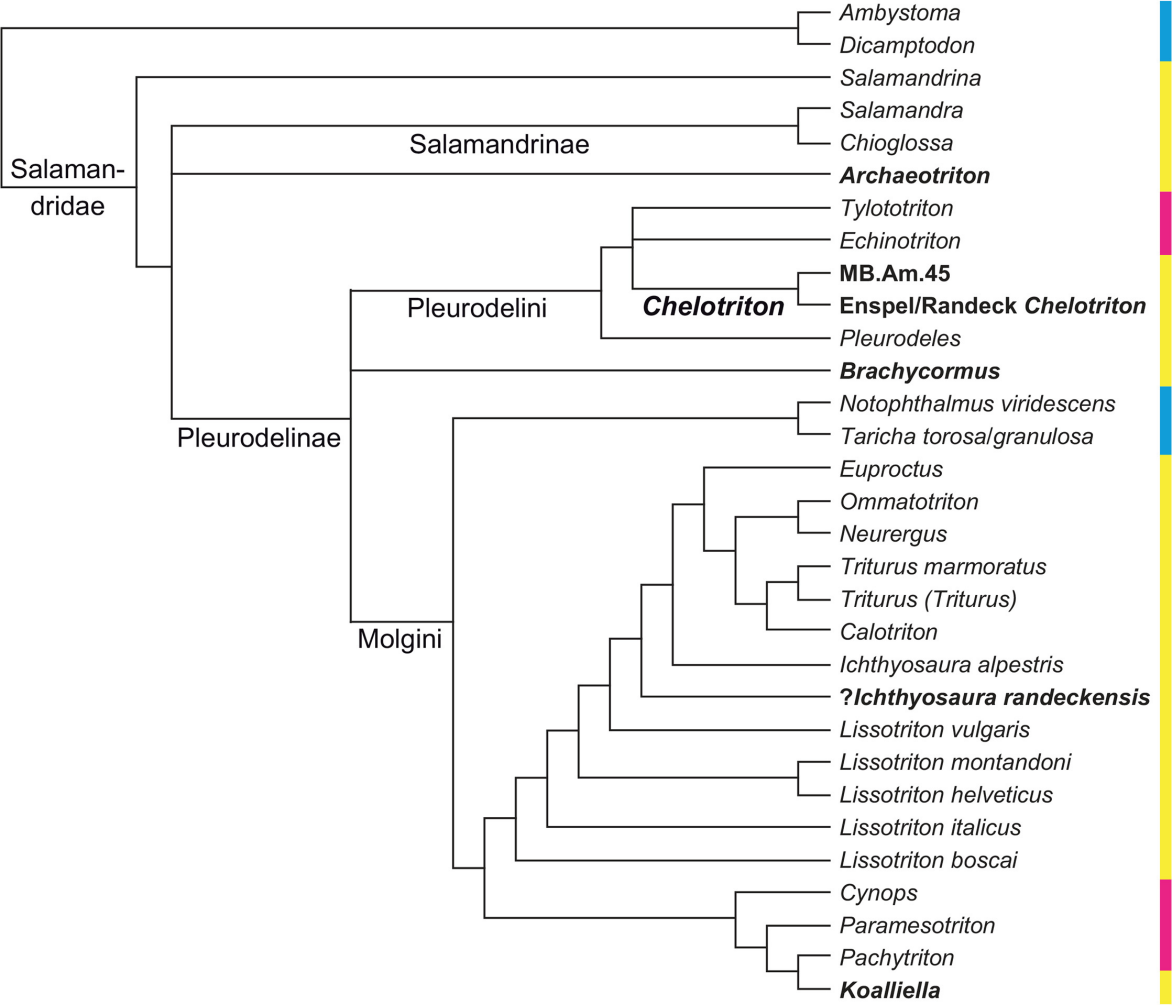
Identical strict and Adams consensus of the pruned most parsimonious trees from our constrained analysis without character 98. *“Triturus” roehrsi*,? *Notophthalmus crassus*,? *N*. *robustus*,? *Taricha miocenica* and *Carpathotriton* were pruned from all trees before calculating the consensus, but after the analysis; see Fig 17 for the strict consensus of the unpruned trees. Names of extinct taxa are in boldface. Colors as in Fig 1.

The Adams consensus of these (S6 Fig) and inspection of individual trees suggest that“*Triturus” roehrsi*,? *Notophthalmus crassus*,? *N*. *robustus*,? *Taricha miocenica* and *Carpathotriton* (all of them extinct and rather poorly known) prevent the backbone constraint (Fig 3) from being part of the strict consensus. Pruning them from the trees (after the analysis) results in a well resolved identical strict and Adams consensus tree (Fig 18), in which *Brachycormus* forms a trichotomy with Pleurodelini and Molgini.

#### Analysis constrained to fit the molecular consensus, with geographic distribution as an additional character

When character 98 is added to the constrained analysis, 588 shortest trees are found (length = 633 steps, including steps within polymorphic OTUs; consistency index = 0.4724; homoplasy index = 0.7694; retention index = 0.5623; rescaled consistency index = 0.2656). In their strict consensus (S7 Fig), all of Salamandridae except *Salamandrina* forms an almost completely unresolved polytomy; the only clades it contains are Salamandrinae, *Chelotriton*, the constrained clade ((*Neurergus*, *Ommatotriton*), (*Calotriton*, *Triturus*)), and a clade that unites all five OTUs of North American salamandrids, extant and extinct.

**Fig 19:**
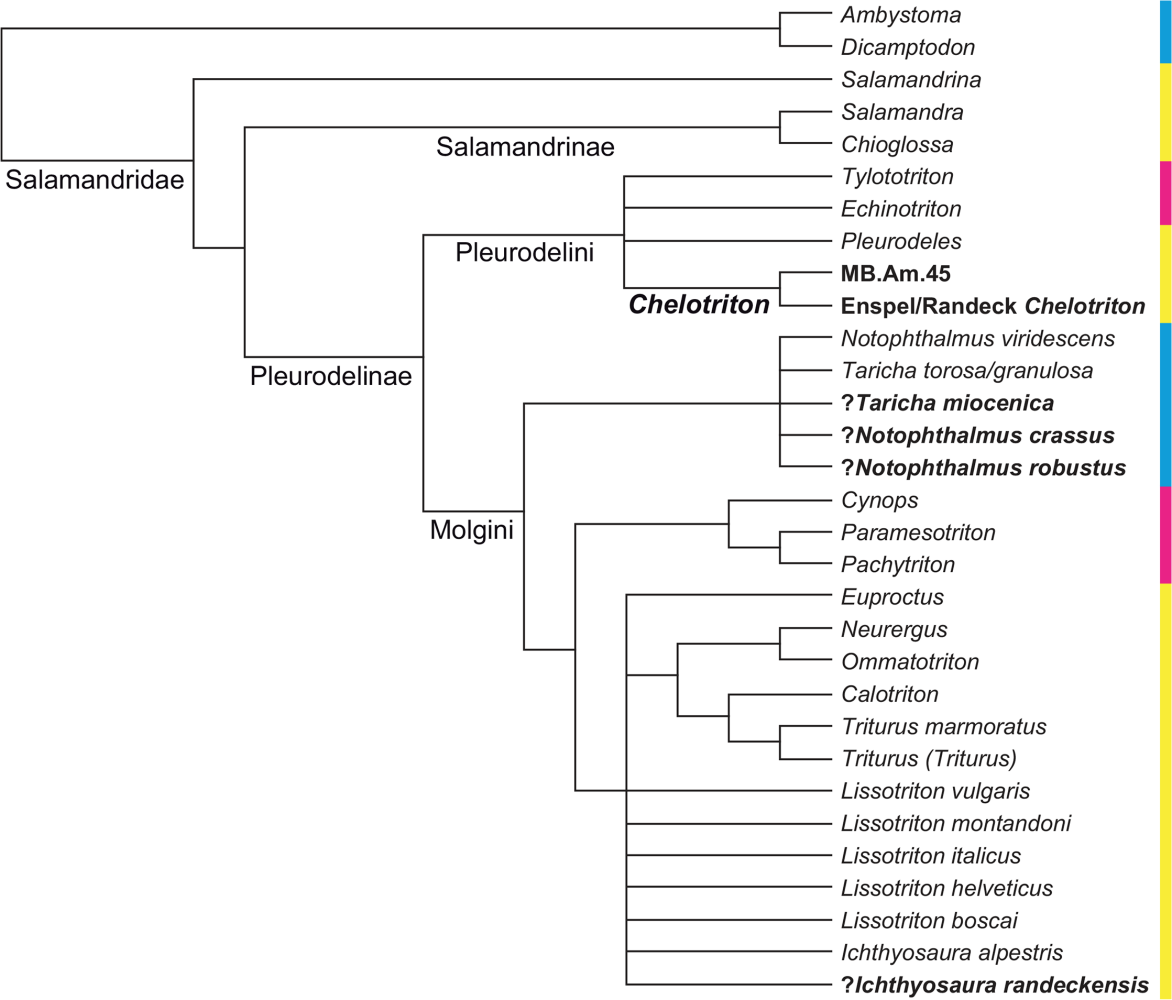
Strict consensus of the pruned most parsimonious trees from our constrained analysis with character 98 included (length of individual trees before pruning = 633 steps). *Koalliella*, *Brachycormus*, *“Triturus” roehrsi*, *Carpathotriton* and *Archaeotriton* were pruned from all trees before calculating the consensus, but after the analysis. Names of extinct taxa are in boldface. Colors as in Fig 1.

Pruning *Koalliella*, *Brachycormus*, *“Triturus” roehrsi*, *Carpathotriton* and *Archaeotriton* from the shortest trees reveals most of the constraint (Fig 19). The exception is that Pleurodelini remains a polytomy. The Eurasian molgin clade is divided into an Asian and a European one, the latter forming a polytomy that contains *Euproctus*, *Ichthyosaura alpestris*,? *I*. *randeckensis*, the ((*Neurergus*, *Ommatotriton*) (*Calotriton*, *Triturus*)) clade, and all five *Lissotriton* species without any further resolution; the Adams consensus of the same pruned trees (S8 Fig) does not clearly suggest further candidates for pruning.

## Conclusions

MB.Am.45 belongs to the generally peramorphic pleurodelin newt *Chelotriton*, as mentioned in the literature once without further comments [29], but is even more peramorphic than the previously referred specimens in several respects. Although some other specimens are considerably larger, MB.Am.45 has jaw joints far caudal to the occiput (unique among caudates, rare among lissamphibians generally), honeycombed sculpture on the maxilla and perhaps the premaxilla (unique among caudates other than *Chelotriton robustus*), and possibly even a septomaxilla (unique among salamandrids; in other urodeles the septomaxilla, where present, is among the last bones to appear in ontogeny). At the same time, the hyobranchial apparatus has not been affected by metamorphosis, indicating an aquatic lifestyle; this combination of peramorphosis and aquatic life is reminiscent of the Jurassic cryptobranchoid [44] *Pangerpeton* [38] and should be explored further.

The previous referral to *Chelotriton paradoxus* [29] is unlikely to be correct, but referral to any species will have to await a revision of *Chelotriton* as a whole.

Our referral of MB.Am.45 to *Chelotriton*, based on diagnostic characters previously suggested for the latter, is confirmed by phylogenetic analysis, which suggests new autapomorphies for *Chelotriton*. Our analyses of salamandrid phylogeny are based on the largest nonmolecular data matrix that has yet been applied to this problem, more than twice as large as any preceding ones; it uses previously published characters mainly from the skull, hyobranchial skeleton and musculature, trunk vertebrae, cloacal anatomy and courtship behavior and contains taxa that had never been included in a phylogenetic analysis before.

Our analyses further confirm that *Chelotriton* is a total-group pleurodelin. Unconstrained analyses (Figs 15 and 16) put it on the stem; an analysis constrained to fit the molecular consensus (Fig 3) but not including a character that codes for geographic distribution places it within the crown instead, in a trichotomy with the extant Asian *Tylototriton* and *Echinotriton* (Fig 17), while a constrained analysis that uses this additional character puts all three extant pleurodelins and *Chelotriton* in an unresolved polytomy (Fig 19).

*Brachycormus*, often found in the same sites as *Chelotriton*, is a stem-pleurodelin according to the unconstrained analyses (Figs 15 and 16); the constrained analyses fail to resolve whether it is a stem-pleurodelin or a stem-molgin (Figs 18 and 19, S7 Fig).

The Oligocene *Archaeotriton* is a member of the European molgin clade according to the unconstrained analyses (Fig 15, S5 Fig), and either a stem-pleurodeline or a stem-salamandrine in the constrained analysis without geography (Fig 17); it is never recovered as the sister-group to the non-pleurodeline, non-salamandrine salamandrid *Salamandrina* as found in [3].

The Miocene pleurodeline *Carpathotriton* [3] is found within the European molgin clade in the unconstrained analyses (Figs 15 and 16), unlike the position in an Asian molgin grade found in [3]; the constrained analyses have trouble resolving its position (Fig 17, S8 Fig).

The likewise Miocene *“Triturus” roehrsi* was referred to, in modern terms, *Lissotriton* in [4,27,86], but to *Ommatotriton* in [65,87]. Including *“T*.*” roehrsi* in a phylogenetic analysis for the first time, we instead find this poorly known taxon to lie outside the smallest clade that contains *Lissotriton*, *Ommatotriton* and all other extant European molgins (Figs 15–17). Further research is needed to determine which taxonomic consequences should be drawn, but in any case this result underscores the importance of phylogenetic analysis in determining the distribution and homology of supposed diagnostic characters. At the same time, *“T*.*” roehrsi* highlights the fact that large amounts of missing data do not by themselves cause poor resolution.–The unconstrained analysis that does not take geography into account (Fig 15) finds *“T*.*” roehrsi* in the Asian molgin clade, where it also nests the European *Koalliella* (see below), *Euproctus* and *Calotriton*; this assemblage, which disappears when a single character (geographic distribution; Fig 16) is added, may be held together by adaptations to an unusually aquatic lifestyle.

The Miocene? *I*. *randeckensis* may not belong to *Ichthyosaura* as originally described [60]; the diagnostic characters proposed for *Ichthyosaura* [60] are more widespread, and our phylogenetic analyses do not find these two taxa as sister-groups, although they often lie only one internode apart (Figs 15–19). We do not draw taxonomic consequences, but would like to encourage further research.

The oldest known salamandrid is *Koalliella* from the Paleocene of Germany and France. It is exclusively known from vertebrae that have apparently been lost [4]. In our analyses without the character that represents geographic distribution (Figs 15 and 17), it is fully resolved as the sister-group of the extant *Pachytriton* from China (constrained analysis: Fig 17) or as the sister-group to a clade composed of *Pachytriton* and the European mountain-brook newts *Euproctus* and *Calotriton* (unconstrained analysis: Fig 15). Given the gap in time and space, we consider this unlikely to be due to phylogenetic signal–but it may well reveal an ecological signal and indicate that *Koalliella* lived permanently in rather small bodies of running water. When geography is taken into account, resolution decreases, and very basal positions–which may be expected from the great age of *Koalliella*–become possible, although *Koalliella* remains within the salamandrid crown-group (S4 and S8 Figs).

The monophyly of *Lissotriton*, represented by five OTUs in our matrix, was not enforced in the constrained analyses because [1] only included one species (while [2] included the same five we did and found them to form a strongly supported clade: Fig 2). In none of our analyses do all *Lissotriton* OTUs form a clade, although they are usually close together. This is most likely caused by the lack of published anatomical data.

Perhaps similarly, our unconstrained analyses fail to find *Taricha* and *Notophthalmus* (one OTU each in our matrix) as sister-groups despite the strong molecular support [1,2] (Figs 1 and 2) and the fact that both live in North America (Figs 15 and 16). Instead, they place them one internode apart. Anatomical data for *Taricha torosa*, *T*. *granulosa* and *Notophthalmus viridescens* have been easier to find than for some *Lissotriton* species; however, *Taricha* and *Notophthalmus* have morphologically diverged to the point that our still small matrix may not be able to overcome long-branch repulsion. The upcoming redescription of? *Taricha oligocenica* (see [88]) will likely provide helpful information.

The most striking discrepancy between the molecular consensus and our unconstrained trees concerns *Euproctus*, *Calotriton* and *Pachytriton*, which form a clade in the latter (Fig 15; *Pachytriton* unresolved in Fig 16, S4 Fig, S5 Fig), while in the former (Fig 3) *Calotriton* is the sister-group of *Triturus*, *Euproctus* occupies an uncertain position in Molgini but is not close to *Calotriton*, and *Pachytriton* is nested among its fellow east Asian molgins. *Euproctus* and *Calotriton* are so similar in morphology and in courtship behavior that they were considered the same genus (*Euproctus*) for a long time before they were separated on molecular evidence alone ([66]; confirmed by [1,2]–Figs 1 and 2). It seems that this similarity consists of correlated adaptations to a life in mountain streams [66]; *Pachytriton* lives in less extreme habitats, but shows aquatic adaptations not otherwise found in pleurodelines (e.g. [50]). It appears that it is not character correlation from heterochrony that distorts the unconstrained trees (“ontogeny discombobulates phylogeny” in the title of [110]), but character correlation from living in mountain streams. This factor and the position of *Salamandrina* may be the main causes for why the constrained analyses result in trees that are 32 (without geography) or 30 steps longer (with geography) than the unconstrained analyses.

Information on the anatomy of several salamandrids that have long been known to science was hard, sometimes impossible to find. The same holds for *Dicamptodon* except its skull. We often had to rely on very old literature, and in a few cases found information on extant taxa only in paleontological publications. Furthermore, when the same species is described in different sources, it often emerges that considerable ontogenetic changes continue to happen after metamorphosis and most likely after sexual maturity. We would like to urge neontologists who work on salamandrids to describe and illustrate the skeleton, not limited to line drawings of the skull as has often been the case, as a source of diagnostic and phylogenetically informative characters.

## Appendix: Characters for the analysis of salamandrid phylogeny, and changes to the original coding

All multistate characters state in their names whether they are ordered or unordered.

Symbols: AS = [61]; B = [16]; GB = [63]; HA = [62]; ÖW = [50]; SR = [60]; TL = [52]; V = [3], WÖ = [51]; YZ = [9]. Numbers behind these symbols refer to the numbers of characters in these matrices (except for ÖW, who did not present a matrix or a list, so we have assigned numbers arbitrarily).

### Head

#### Ornamentation of bone surfaces

**1.** SR1: **Dermal skull roof smooth (0); with pitted ornament on nasals and anterior part of frontals (1); ornamented throughout (2) (ordered).** We have ordered this potentially continuous character.
  Sometimes, only the orbit margins of the frontals are ornamented, not necessarily with pits; this is coded by character 3 and scored as state 0 of the present character.
*Taricha* has state 2 ([67]; contra [49]: plate XXVII).
  While *Euproctus montanus* has state 0 as scored ([57]: 271), *E*. *platycephalus* has state 1 ([57]: 268; not shown in fig 5). We have scored *Euproctus* as polymorphic.
*Cynops pyrrhogaster* has state 2 [57]. In *C*. *wolterstorffi*, all dermal bones of the skull except the parietals are ornamented throughout, and the parietals have almost no surface exposed to the skin, because the attachment surfaces of the jaw muscles meet in a sagittal crest [57]. We have therefore scored state 2 for *Cynops*.
*Triturus marmoratus* has state 1; *T*. *(T*.*)* shows states 1 and 2 ([40]: fig 42; [49]: plate XXV; [57]: 303, 306, figs 29, 31, 32).
*Ichthyosaura alpestris* has state 1 ([57]: 301, fig 28) at least sometimes.
  “[M]ost” bones on the skull roof of *Lissotriton montandoni* “are weakly sculptured” ([57]: 295; our translation). We interpret this as state 2.
**2.** SR2: **Nasals, frontals, and parietals smooth or pitted (0); covered with numerous ridges (1).** State 0 is automatically present in taxa with state 1(0) and therefore scored “?” in them. Often the parietals are smooth while the nasals and (parts of) the frontals are pitted; this is coded by character 4.
  V (page 45) wrote about the frontal of *Carpathotriton*: “Several fragmentary specimens belonging to large individuals bear a fine dorsal sculpture, produced by extremely shallow pits and ridges.” Because this sculpture is “fine” and “extremely shallow” and because pits are present, and in particular because the parietal is smooth (scored 3(0)), we have scored *Carpathotriton* as possessing state 0. Nasals are unknown.
*Triturus marmoratus* has state 1 ([57]: fig 29), *T*. *(T*.*)* has state 0 ([40]: fig 42; [49]: plate XXV; [57]: figs 31, 32).
**3.** V5: **Frontal sculpture absent (0); weak (1); strong (2) (ordered).** A potentially continuous character.
*Euproctus* has state 0 ([49]: plate XXVII; [57]).
  State 2 is present in *Cynops* and in *Triturus marmoratus* [57].
*Triturus (T*.*)* shows states 1 and 2 [57].
**4.** V7: **Parietal sculpture absent (0); present (1).** This character is inapplicable when the entire skull (or all except the lateral edges of the frontals: character 3) is unornamented (SR1(0)) and also when the parietals form a sagittal crest over their entire length and are otherwise covered by muscles–in other words, all cases of 25(1) except *Ambystoma*.
  State 0 is found in *Ambystoma* ([49]: plate XXIII; [68,69]).
*Pleurodeles* is polymorphic [57].
*Cynops* has state 1 where applicable (*C*. *pyrrhogaster*, the species scored by V: [57]: fig 18).
**5.** SR3: **Squamosal smooth (0) or ornamented (1).** In taxa with 1(0), state 0 is always present and therefore scored “?”.
*Triturus (T*.*)* has state 0 ([49]: plate XXV; [57]: figs 31, 32), as does *Calotriton* ([57]: figs 1, 2, 4).
*Pachytriton* is polymorphic [59].

#### Skull shape

**6.** SR4: **Outline of preorbital region rounded (0) or markedly stepped (1).**
  State 1 is considerably more widespread than originally scored: it occurs in *Dicamptodon* [104], *Notophthalmus* (at least in dorsal view: [57]: fig 16A), *Taricha* [67], *Euproctus* ([57]: figs 5, 6), *Cynops* (at least in dorsal view: [57]: figs 17, 18) and arguably both species of *Salamandrina* [40,57]. Additionally, it is found in *Paramesotriton* (at least *P*. *maolanensis* [70]).
*Lissotriton boscai* is polymorphic [40,57].
**7.** B9: **Skull at least as wide as long (0); longer than wide (1).** B called state 0 “broader than narrow” and state 1 “long”, but her intent is clear, and the character carries phylogenetic signal if interpreted this way. We have measured skull width at the widest place of the skull (omitting the quadratojugal/quadrate spines of *Echinotriton* does not change its score), and length along the midline so that the occipital condyles do not contribute to the measured length.
*Ambystoma gracile* has state 0 [68], while *A*. *tigrinum* reaches state 1 [69]. We have accordingly scored *Ambystoma* as polymorphic.
  Polymorphism is also documented in *Brachycormus* [14]; for other reasons, [14] (page 486) considered it “possible that the known specimens of *Brachycormus noachicus* represent more than one species.”
*Neurergus* shows state 0 in *N*. *strauchi* and state 1 in *N*. *kaiseri* [40].
*Pleurodeles waltl* appears to pass from state 0 [40,71] to state 1 [57] in ontogeny; because state 1 is also found in *P*. *poireti* [57], we have scored *Pleurodeles* as having state 1.
*Lissotriton boscai* is said to have state 1 on page 62 of [89], fitting fig 4-20(a) of [89] as well as fig 33 of [40] and fig 20 of [57], but flatly contradicting fig 4-20(b) of [89]. This discrepancy is not mentioned in the text of [89]. We assume that fig 4-20(b), a schematic line drawing of a skull in ventral view, was done from a poorly articulated specimen (in particular after the lower jaw was removed), unlike fig 4-20(a), a schematic line drawing of a skull in dorsal view; consequently, we have scored *L*. *boscai* as having state 1.
*Pachytriton* is polymorphic: *P*. *inexpectatus* has state 1, *P*. *archospotus* state 0, and *P*. *brevipes* is borderline [59].
**8.** B5: **“Paroccipital corners” absent (0); present (1).** B considered “often present” an autapomorphy of Molgini including *Archaeotriton*. The homology of these features is unclear to us; we have scored them exclusively from statements in the text of [40,57].
  These two sources disagree on *Lissotriton helveticus*: [57] (page 295) considered them well developed, [40] (page 54) declared them absent. Given the fact that [57] had both a larger number of specimens and larger specimens available, we have scored state 1, although it may be possible that the difference is geographic–the specimens used in [57] came from central Germany, those used in [40] from northern Spain. (In their table V, GB scored state 1 as well without commenting on this; however, GB used [57] as a source in general, so they may simply have copied the statement found there.)

#### Premaxilla

**9.** SR5-V1-TL1-WÖ1: **Premaxillae paired (0); fused (1).**
  SR scored all OTUs except *Salamandra* as having state 1. This is incorrect for *Tylototriton* [8,72] and *Pleurodeles* [38,71], both of which V, TL and WÖ correctly scored as showing state 0.
**10.** SR6-SR7-SR14-V3-V4-TL5-WÖ4: **Dorsomedial processes of premaxillae contact frontals (0); reach around part or all of premaxillary fontanelle but do not contact frontals (1); are entirely anterior to fontanelle (2); are not visible in dorsal view (3) (ordered).** We have arbitrarily chosen the order of SR over that of V, and ordered the character because it is continuous. These processes are usually called “alary”, but we are not certain whether they are homologous to the widely separated triangular processes of MB.Am.45, which are primary homologues of the alary processes of temnospondyls (see Results and Discussion –Heterochrony in MB.Am.45).
  SR7 described whether the premaxillae meet along their entire lengths (0) or are partially separated by the fontanelle (1). State 0 is automatically present when the present character has states 2 or 3, and state 1 is impossible when the fontanelle is absent (14(0)); the character is thus entirely predictable from the present one and character 14. The same holds for SR14 and the identical V4, TL5 and WÖ4: the nasals contact each other if and only if the fontanelle is short or absent and the premaxillae do not contact the frontals.
  For *Salamandra*, V explicitly scored only *S*. *salamandra*, and correctly [40] scored it as having V3(1), our state 0. *S*. *atra*, however, appears to be polymorphic: [57] showed state 0 in Fig 9, while [40] (fig 21) showed an unusual version of state 1 of the present character in which the frontals and the premaxillae are widely separated. We have scored *Salamandra* as having both state 0 and state 1.
  SR scored *Tylototriton* as possessing state SR14(0), our states 0 through 2, but at the same time as SR6(1), our state 3; the latter is correct [8].
*Pleurodeles* passes from state 2 to state 3 in ontogeny [40,57,71]; we have scored state 3.
  For *Notophthalmus*, [57] described and illustrated state 3 combined with the presence of a fontanelle, while [101] illustrated and vaguely described state 0 combined with the complete absence of a fontanelle. Both illustrated the same species; while [57] attributed his specimens to the subspecies *N*. *viridescens viridescens* and [101] referred his adults to *N*. *v*. *louisianensis*, the latter is at minimum paraphyletic with respect to the former (and to *N*. *v*. *piaropicola*), and the whole complex only began to diversify about 3 Ma ago [73]. Such an amount of morphological diversity is therefore surprising. Lacking the means to research this issue, we have taken both publications at face value and scored *Notophthalmus* as having states 0 and 3.
*Euproctus platycephalus* has states 1 and 2 ([49]: plate XXVII; [57]: 268), while *E*. *montanus* has state 0 [57]. We have scored *Euproctus* as having all three states.
*Ommatotriton* is polymorphic, with *O*. *vittatus* just barely reaching our state 1 ([57]: fig 19) but *O*. *ophryticus* having state 2 ([40]: 62, fig 48).
  V scored *Triturus* as possessing state 0; states 0 and 1 are found in *T*. *marmoratus* (SR, [40], [57]: 303, fig 29), while *T*. *(T*.*) cristatus* and *T*. *(T*.*) carnifex* show state 2 ([40], [57]: fig 31) and *T*. *(T*.*) dobrogicus* has state 1 ([57]: fig 32).
  V scored *Salamandrina* as having our state 3; it shows state 1 ([40,57]; borderline to state 2).
  [57] stated on page 293 that *Lissotriton italicus* has state 0, but showed state 1 (borderline to state 2) in fig 21. Most likely, this happens along the vertical slopes of the fontanelle like in *Taricha* and is therefore invisible in dorsal view; we have scored state 0.
  V scored *Ichthyosaura alpestris* as showing our state 0; while the illustrations in SR and [40] are not identical, both show state 1, as do figs 27 and 28 of [57] (fig 28 is close to state 2). We have scored state 1.
  For *Neurergus*, [40] (figs 12, 15) documented state 2 in *N*. *kaiseri* and state 1 in *N*. *strauchi*. [57] (fig 26) showed state 2 in a possibly less mature specimen of *N*. *strauchi*. [74] showed state 0 in their drawing of *N*. *derjugini* (Fig 1C), but their very blurry photo of ostensibly the same skull (Fig 1A) seems to show a completely different suture pattern in the snout and most likely state 2 of the present character, as far as we can tell. Even though the text agrees with this drawing, which also shows lacrimals (which have never been reported in salamandrids before or since), gigantic nostrils that are not discernible in the photo and a premaxillary fontanelle that seems much larger than in the photo, we have ignored it and scored *Neurergus* as having states 1 and 2.
**11.** SR8-V2: **Dorsomedial processes of premaxillae free (0); sutured or fused for at least 50% of their length (1).** The possibility of a suture was not mentioned in either source.
  This character is not applicable when character 10 has state 3, unless the ontogeny is known.
  When *Notophthalmus* has visible dorsomedial processes at all ([101]: fig 6A –as opposed to [57]; see above), it has a clear case of state 1. We have scored that state.
*Euproctus platycephalus* has state 1, while *E*. *montanus* has state 0 [57]; we have scored *Euproctus* as possessing both.
*Cynops* was scored as having V2(0). It has state 1 ([49]: plate XXVII; [57]: figs 17, 18).
  Both states are apparently found in *Triturus marmoratus* [40,57].
*Lissotriton montandoni* has state 0 ([57]: fig 23).
*Lissotriton helveticus* is polymorphic [40,57].
  For *Lissotriton italicus* and *Neurergus*, similar comments apply as for character 10; we have scored state 0 for the former and 1 for the latter.
**12.** SR9: **Dorsomedial processes of premaxillae parallel (0); diverging to frame more or less deltoid fontanelle (1).** This character is not applicable when character 10. It is also inapplicable in *Ommatotriton vittatus* (therefore *Ommatotriton* as a whole) and *Salamandrina*, where the processes are too short (state 2 is almost reached, see above).
  In the cases where *Notophthalmus* has visible dorsomedial processes at all ([101]: fig 6A –as opposed to [57]; see above), it has a rather extreme case of state 1, although the fontanelle seems to be absent and replaced by paramedian processes of the frontals. We have scored state 1.
  State 0 occurs in *Triturus (T*.*) dobrogicus* ([57]: fig 32).
*Lissotriton helveticus* and *L*. *boscai* are polymorphic [40,57,89].
**13.** SR10: **Dorsomedial processes of premaxillae variable but always broad (0); pointed at their dorsomedial ends (1).** This seems to be what was meant by “spike-like” in the original, although *Cynops* has (if anything) state 1 ([49]: plate XXVII; [57]: figs 17, 18), while *Taricha* appears to be polymorphic, with *T*. *granulosa* having state 0 ([41]: fig 13-3E) but *T*. *torosa* showing state 1 [67].–This character is not applicable when character 10 has state 3.
  When *Notophthalmus* has visible dorsomedial processes at all ([101]: fig 6A)–as opposed to [57]; see above), it has a rather extreme case of state 1. We have scored that state.
*Lissotriton vulgaris* has (borderline) state 1 [40,57].
*Lissotriton helveticus* is polymorphic [40,57].
*Ommatotriton* has state 1 [40,57].
**14.** SR11: **Premaxillary fontanelle absent (0); short (1); enlarged to long slit (2) (ordered).** This is a potentially continuous character, so we have ordered it. Indeed, it is continuous enough that we are not quite sure where the boundary between states 1 and 2 is meant to lie.
*Salamandra* and *Cynops* were scored as having state 0. The former has state 1, the latter states 0 and 1 in different species ([40,57]; [49]: plate XXVII). For *Pleurodeles*, however, the scored state 0 is correct [57]; the specimens depicted in [71] and fig 18 of [40] had not reached full size or skeletal maturity.
  [101] figured *Notophthalmus* as passing from state 2 to state 1 between larval stages II and III and from state 1 to state 0 between the eft stage and the adult stage. [57] (fig 16), however, figured an apparently even more mature individual as having state 2. We have accordingly scored *Notophthalmus* as having states 0 and 2; see above for discussion.
  States 1 and 2 are found in *Ommatotriton* and in *Triturus marmoratus* [40,57].
*Triturus (T*.*)* shows states 1 and sometimes 0 ([40]: fig 42; [57]: 307, figs 31, 32).
*Calotriton* passes from state 2 in small adults ([57]: fig 1) over state 1 in larger ones ([57]: fig 4) to “occasionally” state 0 in “very old” ones of both *Calotriton* and *Euproctus platycephalus* ([57]: 264; our translation; repeated for “old males” in *E*. *platycephalus* on page 268). We have therefore scored both *Calotriton* and *Euproctus* as showing state 0.
*Taricha torosa* has state 2 ([49]: plate XXVII; [67]). In the line drawing of *T*. *granulosa* in fig 13-3E of [41], the slit is interrupted by a bony floor in the middle, but we count this as state 2 as well–probably the drawing merely shows the floor formed by the vomers, which is present in *T*. *torosa* [67].
*Lissotriton boscai* clearly has states 1 and 2 ([40]: fig 33b; [57]: fig 20; [89]: figs 4–20, 4–21).

#### Maxilla

**15.** SR12-V10-TL3-WÖ3: **Caudal end of maxilla contacts quadrate or nearly so (0); lies between quadrate and caudal margin of eye (1); lies rostral to caudal margin of eye (2) (ordered).** V did not distinguish states 1 and 2, SR and TL specified the toothed portion of the maxilla (the teeth are not visible when fossils are preserved in dorsal view). We accept the entire quadratojugal-quadrate bone as the quadrate for the purpose of scoring this character.
*Salamandra* was scored as showing state 0 by all four sources. *S*. *salamandra* and arguably *S*. *atra* (separate OTUs in TL, both scored 0) have state 1 [40,57]. We have changed the score to polymorphism (states 0 and 1) because the drawing of *S*. *“laticeps”* does seem to show state 0 ([21]: plate VIII fig 2 = [4]: fig 16A).
  All four sources scored *Pleurodeles* as possessing state 0. [40,57,71] document state 1 instead, although *P*. *poireti* arguably comes close to 0 ([57]: fig 12).
*Notophthalmus* was scored 0 by SR but 1 by TL and WÖ. None of these sources provided any discussion. [101] (fig 6) depicted a borderline condition between states 1 and 2 in an adult skull (the eye is not shown), [57] (fig 16) showed a condition that seems safer to regard as state 1; we have settled for state 1.
  Similarly, *Taricha*, scored 0 by TL and WÖ, was illustrated as having state 1 by [41] (fig 13-3E; *T*. *granulosa*), [49] (plate XXVII; *T*. *torosa*), [57] (fig 15; likely *T*. *granulosa*) and [67] (*T*. *torosa*).
*Cynops* has state 2 ([57]: figs 17, 18]–perhaps borderline to state 1 in *C*. *pyrrhogaster* (fig 18) but not in *C*. *wolterstorffi* (fig 17).
  States 1 and 2 appear to occur in *Ommatotriton* [40,57].
*Lissotriton montandoni* appears to have state 1 [57].
*Salamandrina terdigitata* seems to just barely have state 1, *Lissotriton helveticus* a little more clearly so, while *Calotriton* shows state 2 [40]. Because *Salamandrina perspicillata* has state 0, however [57], we have scored *Salamandrina* as polymorphic.
*Paramesotriton maolanensis* has state 1 [70]; as we have not seen illustrations of any other *P*. skulls and TL scored *P*. *deloustali*, we have kept the original score of 0 and have assigned both that state and state 1 to *Paramesotriton*.
*Archaeotriton* has state 2 (B).
**16.** SR13-V11-TL4-WÖ40: **Maxilla and pterygoid in contact (0); nearly in contact (1); widely separated (2) (ordered).** SR did not distinguish states 0 and 1, calling them both 0 without quantifying the difference between the states, while V followed TL and WÖ in lumping states 1 and 2, calling them both 0 (1 was “Maxillary–pterygoid joint: present”). We have arbitrarily chosen the order of SR over that of V, TL and WÖ.
  States 0 and 1 occur in *Tylototriton* ([8]; page 9 of the supplementary information of [44]).
  SR scored *Salamandra* and *Pleurodeles* as having state 0. They normally show state 2 and 1, respectively [40,57], at least under our definitions; *Salamandra salamandra* (or perhaps *S*. *infraimmaculata*) may, however, be argued to reach state 1 ([53]: fig 2), so we have scored *Salamandra* as having states 1 and 2.
*Taricha* has state 1 [57,67].
*Euproctus* shows a condition borderline between states 1 and 2 ([49]: plate XXVII); we have scored this as partial uncertainty.
*Cynops* has states 1 and 2 [57]. So does *Salamandrina* [40,57].
  States 1 and 2 occur in *Ommatotriton* [40,57].
  Arguably, *Triturus marmoratus* has state 1 [40,57].
  The same holds for at least some individuals of *T*. *carnifex*, while *T*. *ivanbureschi*, *T*. *macedonicus*, *T*. *cristatus* and *T*. *dobrogicus* have state 2, making *T*. *(T*.*)* polymorphic [40,57,91].
*Lissotriton vulgaris vulgaris* was shown as having a rather extreme case of state 2 (as originally scored) by [40] (fig 39), but as possessing state 1 by [57] (fig 25); [57] (fig 24) additionally shows state 1 (a somewhat borderline case) in *L*. *v*. *graecus* (which is sometimes considered a separate species). Given that the drawings in [57] show longer maxillae, longer pterygoids and longer squamosal processes on the frontals, and that [57] gave measurements for considerably larger specimens than the one illustrated in [40], we think that the latter is skeletally immature and have scored state 1 for *Lissotriton vulgaris*. We have to caution, however, that the specimens available for [40] were from southern Germany, while the specimens of the same subspecies used in [57] came from Bosnia and Slovakia.
*Lissotriton montandoni* and *L*. *italicus* have state 1 [57].
*Calotriton* changes from state 2 to state 0 in ontogeny ([57]: figs 1, 4).
  V scored *Paramesotriton* as having our state 1 or 2, likely following TL who had scored the rather deeply nested *P*. *deloustali* this way. [70] (figs 4, 8), however, showed state 0 in the similarly deeply nested *P*. *maolanensis* and state 1 in the less deeply nested *P*. *caudopunctatus*; [75] reported states 0 and 1 in *P*. *longliensis*, the sister-group of *P*. *maolanensis* [70]. We have cautiously scored states 0 and 1 for *Paramesotriton*.
*Lissotriton boscai* is polymorphic: [40] (fig 33) shows a clear case of state 2, [57] shows a less clear one, and [89] (fig 4–20) shows a fairly clear case of state 1.

#### Skull table

**17.** SR15: **Nasal/frontal suture transversely straight (0); stepped with anterolateral projection of the frontal (1).**
*Taricha granulosa* ([41]: fig 13-3E]) and *Archaeotriton* (B) have intermediate conditions that we count as state 1. In *Taricha torosa* ([49]: plate XXVII; [67]), the process that wedges between nasal and prefrontal may also count as state 1, although it projects from a rostromedial process that wedges between the nasal and the premaxilla.
  According to [57] (fig 16), *Notophthalmus* has state 1; according to [101], the suture is rather too short for state 1 to be possible. We have scored state 1.
*Pleurodeles* has state 1, even though the frontals additionally have a shared median projection ([57]: figs 11, 12). About the same holds for *Triturus marmoratus* [40,57], *T*. *(T*.*)* [40,57] and *Lissotriton italicus* [57].
  Both species of *Euproctus* have state 1 [57]; so do both of the *Cynops* species illustrated in [57]. State 1 is further found in *Ommatotriton vittatus* [57] and to a lesser degree *O*. *ophryticus* [40], leading us to score *Ommatotriton* as possessing state 1 as well.
  Polymorphic and mostly borderline in *Pachytriton* [59].
**18.** SR18: **Frontal flat (0); with anterior depression framing enlarged fontanelle (1).** This is only possible when the fontanelle reaches the level of the frontals and has to be scored as unknown in all other cases (including both outgroups and all OTUs where SR11 has state 0).
  The exception to this is *Pleurodeles*: it has state 1 when the fontanelle is still present (and reaches the frontals [71]), and later in ontogeny the depression remains while the fontanelle closes ([57]: figs 11, 12), so we have kept the score of 1.
  State 1 is also present in *Taricha* ([49]: plate XXVII; [67]) and in *Triturus (T*.*)* where applicable (*T*. *carnifex*: [57]).
**19.** SR20-SR21: **Lateral ridge on frontal and prefrontal absent (0); dorsally rounded (1); with longitudinal groove (2) (ordered).** We see no reason to keep these characters separate.
  Contrary to all other sources about *Taricha*, [58] (page 8 and Fig 1) called the prefrontals “lacrimals” and identified additional bones he called “prefrontals” in the snout of *T*. *torosa*. Not only would these “prefrontals” have a unique size and position (far from the orbit margin!), but the supposed sutures between them and the frontals do not exist in at least one other specimen [67]. We strongly suspect damage to the specimen used in [58], or even a misinterpretation of the ridged dermal sculpture. Lacrimals remain unattested in salamandrids–or indeed in any extant salamanders except Hynobiidae, *Rhyacotriton* and *Dicamptodon* [41,43,44].
  We have assigned state 1 to *Archaeotriton* and *Neurergus*, although their ridges end rostrally before reaching the prefrontal (B, [57]).
**20.** SR16: **Ratio of interorbital width to skull length 0.3 or less (0); 0.31–0.35 (1); 0.36–0.49 (2); 0.5 or higher (3) (ordered).** This is a potentially continuous character, so we have ordered it.
  State 0 was originally called “0.25–0.3”, but in *Dicamptodon* the ratio is well below 0.25 [104]. Likewise, states 1 and 2 were defined as “0.32–0.35” and “0.37–0.49”, but the values 0.31 and 0.36 occur in our matrix. State 3 is new; the most mature specimen of *Brachycormus* has a ratio of 0.51 ([4]: fig 20A), the largest skull referred to *Chelotriton paradoxus* figured in fig 2a_5_ of [29] has 0.54, and MB.Am.45 has 0.78.
*Ambystoma* shows states 0 and 1 in different species [68,69].
*Pleurodeles* and *Taricha* are polymorphic, with *P*. *waltl* and *T*. *torosa* having state 1 ([57]: fig 11; [67]) but *P*. *poireti* and *T*. *granulosa* possessing state 2 ([41]: fig 13-3E; [57]: fig 12). The specimen depicted in fig 15 of [57] likewise has state 2; it is labelled “*Diemyctylus torosus*”, but Bolkay appears to have recognized only this single species for all of what is now called *Taricha*.
  States 1 and 2 are found in *Ommatotriton* [40,57].
*Triturus marmoratus* has states 0 and 1 [40,57].
  With increasing size, *Ichthyosaura alpestris* changes from state 2 ([40]: fig 30) over state 1 (borderline between 0 and 1; [57]: fig 27) to state 0 ([57]: fig 28). We have thus scored state 0.
  [40,89] show *Lissotriton boscai* to have opposite ends of the range covered by state 1, while [57] drew it as having a clear case of state 2. We have accepted both and scored this species as polymorphic.
**21.** SR19-V6-TL2-WÖ2: **Frontosquamosal arch absent (wide gap between bones) (0); narrow gap (1); closed (2) (ordered).** V did not distinguish states 1 and 2.
  One might think that this character duplicates character 22. Indeed, state 2 does not occur together with state 22(0); all taxa with 22(0) have therefore been scored as unknown (inapplicable) for this character. However, the correlation is not perfect: state 2 occurs together with 22(1) in some individuals of *Neurergus strauchi* ([40]; but see below), and state 0 is found together with 22(2) in? *Ichthyosaura randeckensis* (SR). We have therefore not merged the characters.
*Pleurodeles waltl* appears to change from state 1 in the small specimen scanned in [71] to state 2 in the larger ones drawn in fig 18 of [40] and especially fig 11 of [57]; we have kept the latter score, which is also correct for *P*. *poireti* ([57]: fig 12).
  All three states occur in *Euproctus* [57,66].
*Ommatotriton* has states 1 and 2, the latter exclusively in males of *O*. *vittatus* ([40]: 63; [57]).
  Both of these states also occur in *Lissotriton montandoni*, although state 2 is rare ([57]: 295).
*Archaeotriton* has state 2 (B).
*Calotriton* often has states 1 and 2 on different sides of the same individual [57].
*Neurergus derjugini* [74] and *N*. *kaiseri* ([40]: fig 12) have state 0; in *N*. *strauchi*, both state 0 ([57]: fig 26; probably an immature condition) and state 2 ([40]: fig 15) are documented. We have scored *Neurergus* as polymorphic.
*Lissotriton boscai* has states 2, 1 and (rarely) 0 [40,57,89].
  We have scored states 0 and 1 for *Pachytriton* [59]; state 2 is limited to some individuals of a rather deeply nested species ([59]; [76]: fig S1).
**22.** SR17: **Posterolateral process of frontal absent (0); more or less triangular (1); robust (2) (ordered).** We have reworded state 1 from “faint and thin” to delimit it more clearly, leading us to score *Ommatotriton*, *Lissotriton montandoni* and *L*. *italicus* (thin but long and not faint) as having state 2. The distinction between states 0 and 1 was uninformative in SR.
*Euproctus platycephalus* has state 2, *E*. *montanus* state 1 [57]. We have scored *Euproctus* as possessing both.
  “The posterolateral process of frontal [sic], which forms the frontal part of the fronto-squamosal arch, is variably present in recent [= extant] *T*. *marmoratus*” [86]. We have therefore added state 0 to the existing score (1) of *Triturus marmoratus*, even though [57] (page 303; our translation) insisted that the processes are “always well developed”.
*Triturus cristatus* and *T*. *carnifex* have state 1, while *T*. *dobrogicus* usually has state 0 instead [40,57]; we have scored both states for *T*. *(T*.*)*.
  [40] showed state 1 in *Lissotriton vulgaris*, as originally scored, but figs 24 and 25 of [57] showed state 2. For the reasons explained above, we have gone with the latter.
*Lissotriton boscai* has states 1 and 2 [40,57,89].
**23.** SR22: **Combined lateral margin of frontal and parietal concave to gently convex (0) or markedly convex (1).** We have not counted the squamosal process of the frontal and hope to have decided the borderline cases in a reproducible way–the distinction was not quantified by SR. A concave shape occurs in *Chioglossa* ([57]: fig 7).
*Pleurodeles* is borderline; we have opted for state 1, to which the largest skull depicted in the literature ([57]: fig 11) comes closest.
*Euproctus* is polymorphic, with *E*. *montanus* having an extreme case of state 0 and *E*. *platycephalus* a rather extreme one of state 1 ([57]: figs 5, 6). Similarly, both states occur in different species of *Cynops* [57].
*Lissotriton montandoni* and *L*. *vulgaris* have state 0 ([40]; [57]: figs 23–25).
  Across its size range, *Ichthyosaura alpestris* has state 0 [40,57].
*Calotriton* has state 1 ([57]: figs 1, 4).
  We have scored *Carpathotriton* as having state 1 ([28]: fig 3A).
**24.** SR23: **Median suture between frontals and parietals straight (0); sigmoidally curved (1).**
*Notophthalmus* has state 1 [57], as do both species of *Euproctus* [57], both *Salamandra salamandra* and *S*. *atra* [40,57], *Ommatotriton* [40], *Lissotriton italicus* [57], and both *Triturus marmoratus* and *T*. *(T*.*)* ([40,57]; SR: fig 3).
**25.** SR24: **Attachment of jaw adductors to frontals and parietals only marginal (0); extending medially, leaving large parts of parietals unornamented (1).** In figures it is often not clear how the states are supposed to differ, especially when the parietals are unornamented to begin with (state 4(0)) and no sharp ridges delineate the dorsal surface from the fossa to which the jaw adductors attach; we have generally not modified the scores. However, such ridges in the form of a crista sagittalis–quite similar to the situation in *Euproctus*, which was already scored 1, and *Cynops wolterstorffi*, where *Cynops* was already scored polymorphic–are present in *Ambystoma* [68,69], *Dicamptodon* [104] and *Calotriton* ([57]: fig 4); we have scored them as having state 1.
*Notophthalmus* ([57]: fig 16) and *Taricha* ([57]: fig 8; [67]) have state 0.
*Paramesotriton maolanensis* has state 1, displaying a large sagittal crest ([70]: fig 4); yet, V scored *P*. as having ornamented parietals (state 4(1)), implying that no such crest is present. Given the large diversity of *P*., we have cautiously scored polymorphism.
**26.** V8: **Parietal at least as long as frontal (0); shorter (1).**
*Pleurodeles* has state 1 [40,57,71].
  V scored *Triturus* as having state 0. Both *T*. *marmoratus* and *T*. *(T*.*)* have state 1 [40,57].
**27.** V9: **Parietal/squamosal contact absent (0); present (1).**
*Ambystoma gracile* shows state 0 [68], while *A*. *tigrinum* has state 1 [69]; we have scored *Ambystoma* as polymorphic.
*Tylototriton verrucosus* was illustrated as having state 0 in fig 22 of [34] and fig 13 of [57]), but as having state 1 in fig 1b of [8]; we go with the latter, because it appears to show a more mature skull (with more caudally located quadratojugal-quadrate bones and better developed–or at all present–contacts between the latter, the maxillae and the pterygoids).
*Pleurodeles* is polymorphic, with *P*. *waltl* having state 1 [40,57,71], but *P*. *poireti* showing state 0 [57].
*Taricha* is polymorphic, sometimes between the left and the right side of the same specimen of *T*. *torosa* ([58]: 10, fig 1).
  State 1 is present in *Echinotriton* [8].
  The specimen of *Euproctus platycephalus* drawn in plate XXVII of [49] shows state 0 on the left and 1 on the right side; the one drawn in fig 5 of [57] shows state 1 on both sides, while the one of *E*. *montanus* shown in fig 6 of [57] has state 0 on both sides. We have scored *Euproctus* as polymorphic.
*Cynops* has state 1 ([57]: figs 17, 18).
*Ichthyosaura alpestris* was scored 1, but has state 0 according to the drawing in fig 3 of SR.
*Triturus marmoratus* shows both states in the individual figured in fig 45a of [40].
*Calotriton* passes from state 0 to an extreme version of state 1 (a very long suture) in ontogeny ([57]: figs 1, 4).
*Lissotriton helveticus* has state 0 ([40]: fig 36; [57]: fig 22).
*Archaeotriton* has state 0. Note that B used the term “Tympanicum” for the squamosal–following a parochial tradition of salamander research, e.g. [40,49,57]–and, to our knowledge uniquely, the term “Squamosum” for the exoccipital/opisthotic, even though the squamosal is a dermal bone while the exoccipital and the opisthotic are endochondral.
  Both states occur in *Lissotriton boscai* [40,57,89] and in *Pachytriton* [59].

#### Squamosal, quadrate

**28.** V13: **Frontal process of squamosal absent or reduced (0); long (1).**
*Euproctus* is polymorphic ([49]: plate XXVII; [57]: figs 5, 6). So is *Lissotriton boscai* [40,57,89].
**29.** V14: **Posterior process of squamosal absent or reduced (0); long (1).**
*Ambystoma gracile* shows state 0 [68], while *A*. *tigrinum* has state 1 [69]; we have scored *Ambystoma* as polymorphic. *Triturus (T*.*)* is polymorphic as well [40,57].
*Ichthyosaura alpestris* has state 1 (SR: fig 3; [40]: fig 30; [57]: figs 27, 28).
  We have kept the score of 1 for the intermediate condition of *Calotriton* ([57]: fig 4).
**30.** V15: **Anterolateral process of quadrate: absent (0); present (1).**

#### Palate, braincase

**31.** SR 25: **Basal plate of parasphenoid offset (0); continuous with cultriform process (1).** This character was parsimony-uninformative in SR, but is informative here in that state 0 is shared by *Notophthalmus* [57], *Taricha* ([57]; less clearly [67]), *Euproctus* (weakly, but both species: [57]), *Triturus (T*.*)* ([40,57]; [91]: fig 6), *Lissotriton montandoni* [57], *L*. *helveticus* and *Salamandrina* [40] as well as *Dicamptodon* ([5]: fig 8B; [104]) and some species of *Ambystoma* (*A*. *gracile*: [68]; *A*. *tigrinum*: [69]; but not *A*. *maculatum*: [49]: plate XXIII, as “*Amblystoma punctatum*”).
  Arguably, *Pleurodeles* is polymorphic, showing state 0 in *P*. *waltl* but 1 in *P*. *poireti* [40,57,71].
*Cynops* is polymorphic, with *C*. *pyrrhogaster* having state 1 as already scored but *C*. *wolterstorffi* having an extreme case of state 0 ([57]: figs 17, 18).
*Lissotriton vulgaris* is polymorphic as well ([57]: figs 24, 25).
*Neurergus derjugini* [74] and *N*. *kaiseri* ([40]: fig 12) have state 0; in *N*. *strauchi*, both state 0 ([40]: fig 15) and state 1 ([57]: fig 26) are documented. We have scored *Neurergus* as polymorphic.
**32.** SR26-V16: **Posterior ventral crest of parasphenoid absent (0); present (1).** The states of this character are routinely impossible to distinguish in published line drawings; even [57], who did not e.g. omit sculpture as many others have done, mentioned “weak longitudinal and transverse crests” on the parasphenoid (“parabasal”) of *Calotriton* ([57]: 267; our translation), but only drew longitudinal ones in fig 4.
*Ambystoma* is polymorphic ([49]: plates XXI, XXIII; [68,69]).
*Tylototriton* was scored by SR as having state 1, but by V as having state 0. The lightly shaded drawing in fig 22 B of [34] appears to show state 0, so we have kept that score, but we caution that the specimen illustrated in [34] appears to be immature (see character 27).
**33.** SR27: **Pterygoid triradiate (0); with quadrate ramus reduced to a stub (1).** State 0 contains conditions where the basal process is reduced so that the pterygoid is L-shaped or even straight.
  Both states are found in *Ommatotriton* and in *Lissotriton boscai* [40,57,89] as well as in *Pachytriton* [59].
**34.** SR28: **Fontanelle between vomers large (0); tiny (1).** Unsurprisingly, this is a continuous character; we have considered fontanelles “tiny” when they replace less than about 1/3 the length of the suture between the vomers.
  [59,89] called the vomer “prevomer”. This is a remnant of the early 20^th^ century (e.g. [53]: 30 and references therein), when the fused, elongate and very narrow mammalian vomers were mistaken for the parasphenoid, leading to the erroneous belief that they were not homologous to the usually much wider and separate vomers seen in other osteichthyans.–On the homology of the palatines, see [44] and references therein: the palatines, insofar as they are present at all, are not homologous to any part of salamander vomers (including the caudal tooth-bearing processes, contra [29] or [14]: 484, 493), and the name “os vomero-palatinum” [49,57] is misleading.
  Although state 0 is retained, we would like to draw attention to the fact that the fontanelle shrinks strongly in the ontogeny of *Pleurodeles waltl* (compare fig 11 of [57] with fig 18 of [40]).
*Ommatotriton* and *Salamandrina* are (borderline) polymorphic [40,57].
*Triturus (T*.*)* is polymorphic as well, with state 0 occurring in *T*. *cristatus* ([40]: fig 42), in *T*. *carnifex* ([57]: fig 31) and in *T*. *macedonicus* ([91]: fig 6), while *T*. *dobrogicus* has state 1 to the extent that the fontanelle can disappear altogether ([57]: 307, fig 32); state 1 is also found in *T*. *karelinii* or *T*. *ivanbureschi* ([91]: fig 6).
  [57] (figs 20, 23) showed the suture between the vomers to continue caudal to the vomerine toothrow in *Lissotriton boscai* and *L*. *montandoni*. This disagrees with the drawings of *L*. *boscai* by [40,89]. In both species, we have not included the part caudal to the toothrow in our measurement, and have scored both as having state 0.
**35.** SR29: **Tooth-bearing caudal process of vomer S-shaped (0); ʃ-shaped (1); straight, forming a V with the process of the opposite side (2); transverse (3) (stepmatrix).** This character was parsimony-uninformative in SR, where it contained only states 0 and 2. We have recoded it to make it informative and encompass the range of shapes seen in the taxon sample. State 3 is new; it is the plesiomorphic state seen in the outgroups. The states form a state tree with the new state 1 in the middle; all transitions to or from state 1 take one step, all others need to pass through state 1 and therefore require two steps.
*Salamandra salamandra* has state 0, but *S*. *atra* shows state 1 instead ([40]; [49]: plate XXIII; [57]); we have scored both for *Salamandra*.
  State 1 further occurs in *Pleurodeles* ([57]: figs 11, 12), *Euproctus* (both species: [57]: figs 5, 6), *Ichthyosaura alpestris* ([57]: figs 27, 28) and *Salamandrina* [40,57].
*Notophthalmus* appears to pass from state 1 ([49]: plate XXVII; [101]) to state 2 ([57]: fig 16) in ontogeny (while the processes increase greatly in length), so we have kept its score of 2.
  States 1 and 2 appear to occur in *Taricha* ([41]: fig 13-3E; [57]: fig 15; [67]) as well as apparently in *Triturus marmoratus*, *Lissotriton helveticus* and *L*. *boscai* [40,57].
*Calotriton* passes from state 2 to state 1 in ontogeny ([57]: figs 1, 4); we have scored state 1.
  SR scored? *Ichthyosaura randeckensis* as having our state 1 or 2, but wrote on p. 69 that, of the entire palate, only the pterygoid is partially visible in the single known skull that is exposed in dorsal view; this is borne out by their fig 2. We have changed the score to unknown.
*Triturus (T*.*)* spans almost the whole range of shapes known in salamandrids; in particular, *T*. *(T*.*) macedonicus* has an unambiguous case of state 0 ([91]: fig 6). We have scored polymorphism.
*Neurergus* is polymorphic [40,57,74].
  The Enspel and Randeck specimens of *Chelotriton* are intermediate between states 1 and 2 [29,30]; we have scored partial uncertainty.
**36.** SR30-V12: **Orbitosphenoid without posterodorsal extension (0); with small extension (1); with large extension (2) (ordered).** This is a potentially continuous character, so we have ordered it.
**37.** SR31: **Jaw articulation near posterior margin of parasphenoid or caudal to it (0); level with carotid openings (1); anterior to basipterygoid ramus (2) (ordered).** This is a potentially continuous character, so we have ordered it. The addition of “or caudal to it” to state 0 is made necessary by *Dicamptodon* [104] and MB.Am.45.
*Triturus marmoratus* appears to have state 1 [40,57].
  The margins of the parasphenoid are so badly visible in the tiny photo that is fig 4 of [70] that we can only score partial uncertainty between states 1 and 2 for *Paramesotriton*.
**38.** SR33-V19-TL6-WÖ5: **Operculum composed of bone or mineralized cartilage (0) or of unmineralized cartilage (1).**
*Taricha* has state 1 ([58]: 17; [67]).
  We count the “partially mineralized plate” of *Pachytriton* ([59]: 89) as state 0.

#### Lower jaw

**39.** V17: **Dentary tooth row: long (0); at most about half the total length of the dentary (1).**

#### Hyobranchial skeleton

The hyobranchial characters describe the metamorphosed hyobranchium; paedomorphic hyobranchia (Brachycormus, Chelotriton) are scored as unknown.

**40.** TL28-WÖ17: **First basibranchial: bone (0); mineralized cartilage (1); unmineralized cartilage (2) (ordered).** Cartilage commonly mineralizes before it is replaced by bone, therefore we have ordered this character.
  TL assigned state 0 to *Dicamptodon* and state 1 to *Taricha*. In the metamorphosed specimens imaged by [67,104], however, the first basibranchial is entirely invisible, suggesting unmineralized cartilage and thus state 2. [58] (page 21) confirmed cartilage (state 2 or perhaps 1) for *Taricha*, but said that [77] had “reported some ossification”; rather than scoring states 0 and 2 for *Taricha* based on a source we have not seen, we speculate that [77] may have observed mineralizing cartilage instead of bone and have scored *Taricha* as having states 1 and 2.
**41.** V24-TL23-WÖ13: **Second basibranchial present (0); rudimentary (1); absent (2) (ordered).** This is a potentially continuous character, so we have ordered it.
  According to ÖW (page 100), *Euproctus* has states 1 and 2, while only state 2 is known from *Calotriton*.
**42.** V28-TL29-WÖ18: **First hypobranchial cartilaginous (0); bony (1).** Our sources called it a ceratobranchial.
*Archaeotriton* has state 1 (B).
**43.** TL31-WÖ19: **Second hypobranchial: bone (0); partially mineralized cartilage (1); unmineralized cartilage (2) (ordered).** Our sources called this element a ceratobranchial. Cartilage commonly mineralizes before it is replaced by bone, therefore we have ordered this character.
  TL assigned state 0 to *Ambystoma*, *Dicamptodon* and *Pleurodeles*. These taxa have state 2, 1 and 2 instead, respectively [68,69,71,104], as correctly scored by WÖ.
  TL and WÖ both assigned state 1 to *Taricha*. [67] shows state 2.
  Following ÖW (page 101), we have scored states 0 and 2 for *Euproctus*.
*Paramesotriton maolanensis* has state 0; this is an autapomorphy of this deeply nested species [70], so we have kept the score of 2 for the large clade *Paramesotriton*.
**44.** TL32-WÖ20: **Ceratohyal: partially ossified (0); entirely unmineralized cartilage (1).** Other states appear not to be attested.
**45.** V25-TL24-WÖ14: **One ceratobranchial present (0); all absent (1).** All of our sources called it an epibranchial.–The presence of more than one ceratobranchial is not known to occur in metamorphosed salamanders other than hynobiids; we have scored *Chelotriton* (two ossified ceratobranchials) and *Brachycormus* (four) as unknown.
**46.** TL25-WÖ39: **Ceratobranchials shorter (0) or longer (1) than hypobranchials.** TL and WÖ spoke of “epi-” and “ceratobranchials”.
**47.** V26-V27-TL26-TL27-WÖ15-WÖ16: **Two pairs of radii (0); single pair without interradial cartilage (1); single pair with small interradial cartilage (2); single pair with large interradial cartilage (3) (ordered).** Our sources kept the number of pairs of radii and the presence/size of the interradial cartilage separate characters, but WÖ (page 126; see also ÖW) stated: “The interradial cartilage is a functionally significant element found only in forms that have lost the first pair of radii.” The matrices of V and TL do not contradict this statement. We have accordingly merged these characters.–State 2 is limited to *Taricha*; because the character is ordered, state 2 is nonetheless informative.
*Taricha* was scored as having our state 2 by WÖ and, as specifically *T*. *granulosa*, by TL; this was stated to be the case and illustrated by ÖW (page 99, figs 3A, 3B), explicitly contradicting [49] who had illustrated state 0 for *T*. *torosa* in plate XXIV fig 91. (*Taricha* was absent from V’s matrix.) [58], however, reported on page 22 that different individuals of *T*. *torosa* have two (as in [58]: fig 16) or one pair of radii; he did not mention the interradial cartilage. We have scored *Taricha* as having states 0 and 2.

#### Hyobranchial musculature, tongue

**48.** V29-TL34-WÖ23: **M. rectus cervicis profundus inserts via a single head (0) or several (1).**
**49.** TL35-WÖ24: **Number of myocommata in the M. rectus cervicis profundus: 3 (0); 1 (1); 0 (2) (ordered).** We have ordered this character because it is potentially meristic.
**50.** V30-TL36-WÖ25: **M. hebosteypsiloideus more (0) or less (1) differentiated.**
**51.** V31-TL37-WÖ26: **M. inter ossa quadrata: all fibers fall short of the raphe (0); a few fibers extend to the medial raphe (1); the muscle is well developed (2) (ordered).** V dropped state 1, because he did not sample any taxa that showed this state. We have ordered this meristic character.
**52.** ÖW1: **M. subarcualis rectus I “rather delicate” (0); “weakest” among those with “increased strength” (1); stronger (2) (ordered).** A potentially continuous character. “The muscle is rather delicate in the species of *Salamandra*, *Chioglossa*, and *Salamandrina* […] The muscle is rather delicate in both *Pleurodeles* and *Tylototriton*. […] Increased strength characterizes the muscle of the remaining genera. The subarcualis rectus I is weakest in *Paramesotriton* and strongest in *Hypselotriton* and *Pachytriton* among these genera.” (ÖW: 108)–While the latter two are fully aquatic, *Paramesotriton* is not much less so, indicating to us that states 1 and 2 may not be fully correlated to a more aquatic lifestyle than seen in the taxa that have state 0.
  Because *Hypselotriton* is part of our *Cynops* OTU, using the “strongest” condition as a separate state would not have been parsimony-informative, so we have not separated it from state 2. State 2 occurs in those of our OTUs that ÖW (page 92) dissected, OTUs not mentioned there are scored as unknown.
**53.** V33-TL42-WÖ32: **Mm. geniohyoideus and genioglossus unconnected (0); attached by dense connective tissue at anterior ends of ceratohyals (1).**
*Taricha* was scored 1. It seems to have state 0 ([58]: 35, 37, figs 13, 14, 16).
  TL (page 151) coded the condition of *Pachytriton*, “the connection […] present but the genioglossus undifferentiated”, as a separate state. We would prefer to treat the differentiation of the M. genioglossus as a separate (and parsimony-uninformative) character; given that the connection is present, we have scored state 1 for *Pachytriton*.
**54.** TL39-WÖ28: **M. basiradialis “rather small and weak to relatively well developed” (0); “very well developed and strong” (1)** (quotes from WÖ: 127). We suspect that quantifying this character would have revealed more distinguishable states and thus more phylogenetic signal–we had no choice but to copy the scores of TL, which limit state 1 to *Salamandrina* and *Chioglossa*, while the condition is unknown in the outgroups.
**55.** TL45-WÖ35: **Mm. radioglossus and hyoglossus represented by a single, undifferentiated, unpaired muscle (0); two well-differentiated, well-developed muscles (1); a single hyoglossus and paired radioglossus (2); both greatly reduced (3) (unordered).** Even among the first three states, no sequence for ordering is evident, so we have kept this character unordered.
**56.** V34-TL44-WÖ34: **M. rectus cervicis profundus inserts neither on the first basibranchial nor on the radii (0); on the caudal part of the first basibranchial (1); on both the first basibranchial and the radii (2) (ordered).** This seems like a clear meristic character, so we have ordered it.
**57.** TL47-WÖ37: **M. subhyoideus not (0) attached to mandible (1).** TL and WÖ distinguished two modes of attachment, but one is an uninformative autapomorphy of *Chioglossa*.
*Taricha* is polymorphic ([58]: 34).
**58.** TL38-WÖ27: **Tongue without (0) or with (1) free caudal margins; large, free caudal flap (2) (ordered).** We have ordered this potentially continuous character. The original state 1 (“lack of differentiated tongue pad”) is a parsimony-uninformative autapomorphy of *Pachytriton*, which we prefer to score as unknown (inapplicable).

### Postcranial skeleton

#### Vertebrae.

**59.** SR34-V35: **Atlas centrum not shorter than trunk vertebrae (0) or much shorter (1).** SR34 was parsimony-uninformative because state 0 was restricted to *Salamandra*. State 0 is also present in *Ambystoma* [69], *Taricha* ([58]: fig 2; [67]), *Salamandrina* (V) and *Archaeotriton* (B).
**60.** YZ1-YZ4: **Cranial end of neural spine at cranial border of neural arch (0); between cranial border of neural arch and caudal margin of articular surfaces of prezygapophyses (1); at same level or caudal to caudal margin of articular surfaces of prezygapophyses (2) (ordered).** YZ treated this clearly continuous character as two binary ones.–All vertebral characters concern the sixth presacral (YZ); when that particular vertebra is not (or not certainly) available, we have used information from other mid-trunk vertebrae, even the unspecified trunk vertebrae drawn in [40], but we have generally tried not to stray too far from the sixth presacral.
*Neurergus kaiseri* has state 2, *N*. *strauchi* state 1 ([40]: figs 14, 17); because YZ, who scored state 0 for *Neurergus*, scored another species (probably *N*. *crocatus*: YZ: 133, fig 1.5), we have scored *Neurergus* as possessing all three states.
**61.** YZ2-YZ3: **Mediolateral width of neural crest similar along its craniocaudal length, not broadened into a spine table (0); much wider along a line between the diapophyses than cranial to the diapophyses, but dorsal surface not sculptured (1); dorsal surface sculptured (2) (ordered).** In the original, these two characters are exact duplicates of each other (they have the same states in the same taxa), but sculpture is absent (our state 1) on the spine tables of *Notophthalmus*, *Salamandrina* and *Neurergus*? *crocatus* according to YZ’s own fig 1 (confirmed for *Salamandrina* by [4]: fig 17F; [40]: 48, fig 29). B (page 271) further explicitly reported state 1 in *Archaeotriton*, and [4] (fig 21D) showed state 1 in *Lissotriton montandoni*; arguably, it also occurs in *Ambystoma gracile* [68], though not in *A*. *tigrinum* (which has state 0) [69]. Because sculpture does not occur when the neural spine or crest is not broadened into a spine table, we have merged the characters; we have ordered the resulting character because spine tables occur without sculpture but sculpture does not occur without spine tables.
  This character seems to be subject to considerable regional and/or ontogenetic variation. YZ scored and illustrated (YZ: fig 1.3) state 1 for the specimen of *Cynops pyrrhogaster* they had examined; V (fig 10G) showed state 2 in another specimen. Conversely, V (fig 10A) showed state 0 in a specimen of *Taricha granulosa*; YZ examined that same specimen and another, and illustrated and scored state 2! We have assigned state 2 to both *Cynops* and *Taricha* in order to avoid scoring skeletally immature individuals.
*Neurergus kaiseri* and *N*. *strauchi* have state 0 ([40]: figs 14, 17); because YZ scored another species (probably *N*. *crocatus*) (YZ: 133, fig 1.5), we have scored *Neurergus* as possessing states 0 and 1.
**62.** YZ5: **Neural spine height at tallest point of caudal half ≤ 0.35 times maximum vertebral length (0); > 0.35 times (1).**
**63.** YZ6: **Cranial edge of neural spine approximately vertical (parallel to dorsoventral axis) (0); clearly inclined cranioventrally to caudodorsally (1).**
*Ommatotriton ophryticus* has state 1 according to [40] (fig 50), but state 0 according to [78] (fig 13); the two drawings, as well as the accompanying descriptions, differ drastically in this character and the next, while being largely identical otherwise. Neither source states the ontogenetic age of the individual or the position of the illustrated vertebra in the column; our only choice is to accept both. YZ scored *O*. *vittatus*, of which we have not seen an illustration, as having state 0; we have scored *Ommatotriton* as polymorphic.
*Triturus karelinii*, and thus *T*. *(T*.*)*, is polymorphic ([78]: fig 9).
*Neurergus kaiseri* may have state 1 as scored for *Neurergus* ([40]: fig 14), but *N*. *strauchi* clearly has state 0 ([40]: fig 17). We have scored polymorphism.
*“Triturus” roehrsi* is apparently polymorphic ([33]: fig 25; [86]: figs 27, 28).
  We have counted the overhang of the referred *Koalliella* specimen depicted in fig 22D of [4] as state 0. Under the assumption that the referral is correct, we have ignored the lectotype ([4]: fig 22C) for the purpose of scoring this character, because it seems generally less mature–although it is considerably larger, making regional or indeed taxonomic variation an additional possibility.
**64.** YZ7: **Dorsal edge (or spine table) of caudal half of neural spine horizontal (0) or not (1).**
*Ommatotriton ophryticus* has state 1 according to [40] (fig 50), but state 0 according to [78] (fig 13); the two drawings, as well as the accompanying descriptions, differ drastically in this character and the preceding one, while being largely identical otherwise. Neither source states the ontogenetic age of the individual nor the position of the illustrated vertebra in the column; our only choice is to accept both. YZ scored *O*. *vittatus*, of which we have not seen an illustration, as having state 0; we have scored *Ommatotriton* as polymorphic.
*Triturus karelinii*, and thus *T*. *(T*.*)*, is polymorphic as well ([78]: fig 9).
**65.** YZ8: **Cranial edge of neural arch in the midline (“neural arch notch”) cranial (0) or caudal (1) to centres of articular surfaces of prezygapophyses.**
*Ommatotriton ophryticus* has state 1 ([40]: fig 50; [78]); because we have not seen an illustration of *O*. *vittatus* (scored as having state 0 by YZ), we have scored *Ommatotriton* as polymorphic.
*Triturus (T*.*)* is polymorphic ([78]: figs 7–9).
**66.** YZ10: **Articular surfaces of diapophysis clearly larger than that of parapophysis (0); similar in size (1); clearly smaller (2) (ordered).** Strikingly, YZ treated 0 and 2 as the same state.–We have ordered this continuous character.
**67.** YZ11: **Caudal end of dorsal lateral crest dorsal to diapophysis (0); at same level (1); ventral to diapophysis (2) (ordered).** We had to change the order of the states so we could order this clearly continuous character.
**68.** YZ12: **Notch between diapophysis and parapophysis present (0); absent (1).**
**69.** YZ13: **Diapophyses or parapophyses extend laterally beyond a line along the lateral edges of the zygapophyses by < 16.5% (0); 16.5%–22.5% (1); > 22.5% of the maximum width between any zygapophyses (2) (ordered).** A continuous character.
*Ommatotriton ophryticus* has state 0 according to ([40]: fig 50). The asymmetric vertebra illustrated in fig 13B of [78] has state 0 on the left but state 2 on the right side; the ostensibly same but symmetric vertebra in fig 13A of [78] has state 1 on both sides. We have not seen an illustration of *O*. *vittatus* (scored as having state 1 by YZ), but have scored *Ommatotriton* as having all three states.
  We have assigned state 2 to MB.Am.45; zygapophyses are not visible, but very long rib-bearers are.
**70.** YZ14: **Ventral curvature of centrum: concavity does not (0) or does (1) reach or exceed half of cotyle height.**
*Ommatotriton ophryticus* has state 0 according to [40] (fig 50), but state 1 according to [78] (fig 13). We have not seen an illustration of *O*. *vittatus* (scored as having state 1 by YZ), but have scored *Ommatotriton* as polymorphic.
  Strong curvature may be absent in *Triturus cristatus* ([78]: fig 7), as originally scored, but not in *T*. *dobrogicus* or *T*. *karelinii* ([78]: figs 8, 9); we have scored *T*. *(T*.*)* as polymorphic.
  We have scored *Pachytriton* as polymorphic as well: YZ illustrated and scored state 1 for the 7^th^ presacral vertebra of *P*. *brevipes*, while [59] illustrated a clear case of state 0 (almost no concavity at all) for the 9^th^ presacral of the same species.
*Koalliella* is polymorphic, assuming that the referred specimens really belong to it ([4]: fig 22).

#### Ribs

**71.** SR35: **Trunk ribs are short rods (0); as long as three vertebral centra (1).**
  The lost skeleton of *Salamandra “laticeps”* (cautiously referred to *S*. *sansaniensis* by [4]) had state 1 (the ribs are at least as long as four centra; [21]: plate VIII fig 2 = [4]: fig 16A). We have therefore scored *Salamandra* as polymorphic.
**72.** TL10-WÖ6: **Caudal ribs present (0); absent (1).** TL and WÖ called the ribs “caudosacral”, but neither these ribs nor their vertebrae participate in any kind of sacrum; such ribs on proximal caudal vertebrae are called “caudal” in other tetrapods sensu lato (e.g. [79]).
  We have not counted the usually present, mobile and free rib pair of the “second sacral” (first caudal) vertebra which is commonly present when other caudal ribs are absent [66].
  TL scored *Ambystoma* as unknown. [69] showed clearly that caudal ribs are absent (state 1) at least in *A*. *tigrinum*, confirming the score for *Ambystoma* in [44].
  TL also scored *Dicamptodon* as unknown, while [44] scored it as having our state 1. We have gone with the latter for the time being.
  [44] also scored our state 1 for *Tylototriton*, directly contradicting TL. Because [44] cited descriptions of the skeleton of two species of *T*. that were published long after TL, we have accepted this score. This restricts state 0 to *Pleurodeles* and *Salamandrina*.

#### Appendicular skeleton

**73.** SR36: **Ilium slender with elongated shaft (0); stout with short and broadened shaft (1).**
**74.** SR38: **Four or more ossified tarsals per foot (0); three or fewer (1).** Ossification was not specified in the original, but must have been meant.

### Soft anatomy

#### General

**75.** TL46-WÖ36: **M. depressor mandibulae: two heads (0); one head (1).** Within our state 0, TL and WÖ distinguished the condition of *Salamandrina* (a skeletal and a cutaneous head) from that in Salamandrinae (two skeletal heads), but no sequence for ordering is evident, so the condition of *Salamandrina* would be parsimony-uninformative. Assuming that the two heads are homologous between *Salamandrina* and Salamandrinae, which seems quite likely from ÖW (Fig 7), we have merged the states found in these taxa.
**76.** ÖW2: **M. depressor mandibulae “somewhat weaker” (0); “large and strong” (1); “very strong” (2) (ordered).** This potentially continuous character is only applicable when this muscle has a single head (state 75(1)). “The muscle is very strong in *Pleurodeles* and *Tylototriton* […]. The muscle is large and strong in *Paramesotriton*, *Hypselotriton*, and *Pachytriton*, and somewhat weaker in the other genera.” Following this statement from ÖW (page 105), we have scored this character for the taxa they treated; “*Tylototriton*” of ÖW included *Echinotriton* (they dissected both: ÖW: 92), and *Hypselotriton* is part of our *Cynops* OTU, which thus has states 0 and 1.
**77.** ÖW3: **Single head of M. depressor mandibulae: narrow (0); wide (1).** This, too, refers to state 75(1), and it, too, is scored after ÖW (page 105): “The muscle […] in *Pleurodeles* and *Tylototriton* […] with narrow origins […] *Triturus*, *Paramesotriton*, and *Pachytriton* have a depressor mandibulae with a narrow origin […]. *Euproctus*, *Notophthalmus*, *Neurergus*, *Taricha*, *Cynops*, and *Hypselotriton* have a triangular muscle with a broader origin.” For the compositions of these taxa we have again relied on their list of dissected species (ÖW: 92).
**78.** V32-TL40-WÖ30: **Mm. recti abdominis profundus and superficialis differentiated and separate (0); not distinct (1).**
**79.** V20-TL8-WÖ8: **Lungs well developed (0); weakly developed or absent (1).**
**80.** V21-TL9-WÖ9: **Skin smooth (0); rough or keratinized on at least part of the body in some ontogenetic stages (1).** This character was parsimony-uninformative before we added *Ambystoma* (which shares state 0 with *Salamandra*).
  State 0 is present in *Paramesotriton maolanensis*, but not any other species of *P*.; given the deeply nested position of *P*. *maolanensis*, this is clearly an autapomorphy, and *P*. is ancestrally rough [70]. We have kept the score of 1.
**81.** GB34: **Spur on the hindlimbs of adult males in the breeding season: present (0); absent (1).** Without further commenting on this character, GB (tables I, II) ascribed state 0 to *Tylototriton*, “*Euproctus*”, “*Triturus*”, *Neurergus*, *Notophthalmus*, *Taricha*, and *Hypselotriton* (part of our *Cynops* OTU), leaving state 1 to *Cynops* sensu stricto, *Paramesotriton* and *Pachytriton*. While state 0 is correct for *Euproctus*, *Calotriton* has state 1 [66]. We have scored polymorphism for *Cynops*. It is not at all clear which species of “*Triturus*” GB used to score this character; we have assumed that all species they used for their analysis of the phylogeny of “*Triturus*” have state 0, namely *Ommatotriton*, both of our *Triturus* OTUs, all five of our *Lissotriton* OTUs, and *Ichthyosaura alpestris* (GB: table V).
**82.** TL13-WÖ10: **Eggs “small” (0); “medium” (1); “large” (2) (ordered).** WÖ did not define these terms; with one exception, we have adopted their scores without changes or additions (other than those made by TL), except for ordering this potentially continuous character. The exception is *Echinotriton*: [8] (pages 324–325) called its eggs “large”, adding that they are deposited singly and on land, contrasting them with those of *Tylototriton*, which they called “small” and which were so scored by TL and WÖ. This description makes state 2 more likely than 1 for *Echinotriton*.
  [80] (Table 1) published the egg diameters of several salamandrid species. Frustratingly, these data correlate very poorly with the scores of TL and WÖ. We speculate that this character is meant to give some idea of the ratio of adult size to egg size. Rather than compiling different measures of adult size (snout-vent length, mass…) and comparing them to the egg size data of [80], we have ignored the latter, except for further confirmation that the eggs of *Echinotriton* (*E*. *chinhaiensis*: 3.4 mm [80]) most likely count as “large”.

#### Cloaca

**83.** V22-TL18: **Epidermal lining in anterior half of female cloacal chamber: present (0); absent (1).**
**84.** V23-TL19: **Pseudopenis absent (0); present (1).**
  Both states are found in *Euproctus* [66].
  TL and V scored state 1 for *Calotriton*, and indeed state 1 is explicitly stated to be present and illustrated by their source ([65]: 238–239, fig 10D). In spite of this, [66] (page 577) listed “the presence of a conspicuous pseudopenis” among the features that distinguish *Euproctus montanus* from *Calotriton*. We conclude that [66] merely did not think the pseudopenis of *Calotriton* was “conspicuous”; we have kept the score of 1 for it.
**85.** TL17-TL18: **Cranial half of cloacal chamber lined with ciliated mucosa (0); unciliated mucosa (1); keratinized epidermis (2) (ordered).** As the original source of this character [65] made clear, states 0 and 2 are extremes in a potentially continuous series; keratinized epidermis cannot bear cilia, and ciliated mucosa cannot be keratinized.
  Note that the original description of character TL17 (TL: page 150: absence of cilia ascribed to *Calotriton* and *Salamandra*) and the original scores of the same character (TL: page 129: state 1 –absence of cilia–scored for *Triturus (T*.*) karelinii* and *Lyciasalamandra*, with *Neurergus* and *Mertensiella* scored unknown and all other OTUs given state 0) contradict both each other and the statements by both of the cited sources: “Ciliated epithelium in the cloacal tube and/or anterior cloacal chamber of females” occurs in *Lyciasalamandra*, *Pleurodeles* and *Salamandrina* according to [64] (pages 172–173), while “I did not find cilia in females from the other nine genera examined”; [65] (page 233) repeated that cilia anywhere in the cloaca occur in *Lyciasalamandra*, *Pleurodeles* and *Salamandrina*, not mentioning any taxa as lacking cilia (except by stating that the “small cloacal chamber of *E*. *asper* [= *Calotriton*] is entirely lined with epidermis”). It has not escaped our attention that, in the matrix of TL (page 129), *T*. *(T*.*) karelinii* is one line below *Calotriton*, while *Lyciasalamandra* is one line above *Salamandra atra* (and two above *S*. *salamandra*, which is scored wholly identically to *S*. *atra*); maybe TL put the intended score into the wrong line twice. Similarly, the comments on character TL17, including the statements about [64,65], are correct for TL18.
  We have scored state 0 for *Pleurodeles* and *Salamandrina* (*Lyciasalamandra* is absent from our matrix) as well as for *Ambystoma* and *Dicamptodon* ([64]: Table 2), state 2 for *Salamandra*, *Euproctus* and *Calotriton*, and state 1 for *Tylototriton*, *Notophthalmus*, *Taricha*, *Cynops*, *Triturus (Triturus)*, *Lissotriton vulgaris*, *L*. *boscai*, *Ichthyosaura*, *Paramesotriton*, *Pachytriton* and *Chioglossa* because [65] (Table 1) examined specimens of them, and tentatively also for *Neurergus* because [64] (Table 1) cited a reference that described the anatomy of the female cloaca.
**86.** TL20: **Female cranioventral glands present (0); absent (1).**
**87.** TL21: **Tubular glands secreting into caudal angle of cloaca absent (0); present (1).**
**88.** TL22: **Male posterior ventral glands present (0); absent (1).**

### Reproductive behavior

**89.** TL14-HA1: **No amplexus (0); ventral amplexus (1); dorsal amplexus (2); restraint of the female by the male using tail, hind limbs and jaws (3) (unordered).** No evolutionary scenario is evident to us, so we have kept this character unordered.–State 3 is found in both *Euproctus* and *Calotriton* ([66]: 569, 571).
*Salamandra*, which is polymorphic with states 1 and 2 (dorsal capture followed by ventral amplexus), *Ommatotriton*, *Lissotriton* (all extant species) and *Triturus marmoratus*, all of which have state 0, *Paramesotriton*, which is polymorphic with states 0 and apparently 3, and *Chioglossa*, which has state 1, are scored after [81]; *Neurergus* and by implication *Pachytriton* have state 0 according to [82]. Many of these, including state 0 for *Pachytriton*, are confirmed by HA, who also showed that *Salamandra* has states 1 and 2 while *Notophthalmus* shows states 2 and 0.
  States 0 and 2 are found in different species of *Ambystoma* (TL, HA); however, the phylogenetic tree in [2] confirms the conclusion by HA that state 0 is plesiomorphic for the clade. We have therefore scored *Ambystoma* as possessing state 0 only.
**90.** HA2: **Sperm deposition: under female’s cloaca (0); in front of female’s snout (1); elsewhere (2) (unordered).** In an additional state, “the spermatophore is deposited directly on or near the female’s cloaca”–apparently always on the female’s body rather than the bottom–after which it “is massaged into the female’s cloaca by the male’s hind feet” (HA: 398). This occurs only in *Euproctus* and *Calotriton* [66], thus only together with state 89(3), and is presumably impossible with all other states of character 89; in particular, in ventral amplexus, the ventral side of the female faces the dorsal side of the male, dorsal amplexus creates the inverse problem, and absence of amplexus would make direct sperm transfer difficult–at least in water. We have therefore scored *Euproctus* and *Calotriton* as inapplicable (“unknown”).
  “*Dicamptodon* courtship has not been observed. All that we know of courtship is that many spermatophores are deposited and that these resemble the spermatophores of *Ambystoma* (R. Nussbaum, personal communication)” (HA: 409). This excludes state 0 and makes state 1 quite unlikely; we have accordingly scored *Dicamptodon* as having state 2.
  HA reported that states 0 and 2 occur in different *Tylototriton* species, but unfortunately did not mention any species by name. Being unable to reconstruct the ancestral state for *T*., we have scored *T*. as having both of these states.
*Neurergus* and “all species of *Triturus*”–meaning *T*. *(T*.*)*, *T*. *marmoratus*, *Ommatotriton* and all species of *Lissotriton*–are scored after [82] (the quote is from the abstract). For *Neurergus*, this agrees with the text of HA, but not with their table 10.2, which evidently contains an error.
**91.** HA3-AS15: **Sperm transfer: cloaca-nudging or tail-nudging walk (0); male turns 90° to brake female (1); pinwheel (2) (ordered).** This character is inapplicable when deposition equals transfer, which is the case for taxa with states 89(3) and 90(0).
  State 0 as well as a different one–“courtship frenzies”–are found in different species of *Ambystoma* (HA: 408); the phylogenetic tree in [2] confirms the conclusion by HA that state 0 (cloaca-nudging walk, as opposed to tail-nudging in *Cynops*, *Paramesotriton* and *Pachytriton*) is plesiomorphic for the clade. We have therefore scored *Ambystoma* as possessing state 0 only.
  The fine-tuned choreography of state 2 is unlikely for *Dicamptodon* (see character 90); we have accordingly scored partial uncertainty between states 0 and 1.
*Neurergus* has state 1 and additionally engages in a tail-nudging walk before spermatophore deposition [82]; this is not state 0 of the present character, agreeing with the text of HA, but not with their table 10.2. We have scored state 1 only.
**92.** HA4-AS1-AS3-AS4: **Tail-fanning display absent (0); “abbreviated” (1); present, not “abbreviated” (2); fanning and flicking (3); fanning and whipping (4); whipping only (5) (ordered).** Apparently inapplicable to *Euproctus* and *Calotriton* (HA: 403). HA described states 0 to 2 (mentioning but not quantifying state 1), AS described states 2 to 5 and tentatively suggested the ordering sequence. HA (fig 10.8A) illustrated a behavior they called “tail-fanning display” in *Triturus (T*.*) cristatus*, while AS and [81] stated that tail-fanning is absent in *T*. *(T*.*) cristatus*; judging from AS, the behavior shown is “rocking”/“tail-snapping” (character AS3) and probably not homologous to fanning.
**93.** AS2-AS5-AS6: **Side-on display: absent (0); wave posture (1); lean-in display with cat-buckle posture (2); cat-buckle posture also occurs outside of lean-in display (3) (ordered).** AS did not suggest the ordering of states 0 to 2, but the lean-in display seems to consist of the wave posture with additions and without subtractions (AS: 652–653).–Most likely inapplicable to *Euproctus* and *Calotriton*.
**94.** AS7: **Rocking/tail-snapping absent (0); present (1).** Most likely inapplicable to *Euproctus* and *Calotriton*.
**95.** AS10-AS12: **Wiggling of upwards-bent tail tip (“distal lure”) absent (0); present (1); present, additionally combined with flamenco (2) (ordered).** The homology of the flamenco is somewhat speculative; we have followed AS in not including the posture sometimes seen in *Triturus marmoratus*, where the tail tip does not move.–Most likely inapplicable to *Euproctus* and *Calotriton*.
**96.** AS11: **Retreat display absent or rare (0); common (1).** Retreat display can be difficult to recognize, so it may have been overlooked in many species where it is not common (AS: 655); we have evaded this problem by not distinguishing rare occurrences from absence.–Most likely inapplicable to *Euproctus* and *Calotriton*.
**97.** AS16: **Pushback: absent (0); present (1).** This is only applicable to taxa with state HA3(1).

### Geographic distribution

**98.** New character: **Geographic distribution: North America (0); Europe, Mediterranean, Iran (1); eastern Asia (2) (unordered).** Different orderings would be appropriate for the Paleogene (1-0-2) and the Neogene (1-2-0), and yet another order (0-1-2) would be necessary for dispersal processes that, say, began in the Eocene and were completed in the Miocene; we have simplified this by making this character unordered.

### Excluded characters

These characters occur in our sources, but are parsimony-uninformative for our taxon sample. To this list we have added a new character that was a promising candidate for inclusion but turned out to be parsimony-uninformative as well.

SR32-V18: **Prearticular-angular not (0) fused (1) with articular.** In both SR and V, this character was parsimony-uninformative (ignoring the hypothetical all-zero outgroup in V), with state 0 being restricted to *Salamandra*. [44] (page 12 of the supplementary information) reported that state 1 of this character, identical to state 1 of their character 29, is found in *Dicamptodon* (in the cases where the articular ossifies at all, which is not common ([104]: comments on the sagittal cutaway view), and that *Ambystoma* is polymorphic. With state 0 being limited to *Salamandra* (part of the ingroup) and part of *Ambystoma* (itself part of the outgroup, together with *Dicamptodon*), this character is parsimony-uninformative for the current taxon sample.

SR37: **Neural spines low and posterodorsally ascending (0); tall with thin crest forming straight horizontal dorsal margin (1).** This character was an attempt to express the variation coded in characters 62–64, which vary (to some degree) independently of each other: for instance, *Cynops pyrrhogaster* has tall, thin, posterodorsally ascending neural spines that have a horizontal posterodorsal margin (B: fig 9).

TL7-WÖ7: **Fifth toe present (0); absent (1).** As TL stated on page 149, state 1 is restricted to *Salamandrina*, making the character parsimony-uninformative.

TL11-WÖ29: **Rib protrusion absent (0); present (1).** As TL stated on page 149, state 1 is restricted to *Pleurodeles* in their taxon sample, making the character parsimony-uninformative there. In our expanded sample, rib protrusion occurs in *Echinotriton* [8,45]. However, rib protrusion requires long ribs; this character is therefore inapplicable to taxa with state 70(0). (*Salamandrina* has ribs of intermediate length–claims of rib protrusion have turned out to be false: [8]; TL: 149.) Furthermore, as a behavioral/soft-tissue character, it is only applicable to extant taxa (absent histological investigations of rib tips that might reveal muscle attachments and thus state 0). This leaves only *Pleurodeles*, *Echinotriton* and *Tylototriton*; the first two have state 1, making state 0 an autapomorphy of *Tylototriton* [8]–the character is parsimony-uninformative after all.

TL12: **Cutaneous papilla projecting dorsally over base of tail absent (0); present (1).** TL scored state 1 only for *Mertensiella* and *Lyciasalamandra*, both absent from our matrix. HA (page 398), contradicting TL, claimed state 1 also for *Salamandra*; if this is correct, state 1 would be an autapomorphy of *S*. in our matrix, so the character would still be parsimony-uninformative.

TL15: **Oviparity (0); ovoviviparity (1).** State 1 occurs in *Mertensiella*, *Lyciasalamandra* and *Salamandra*; with the first two absent from our matrix, the character becomes parsimony-uninformative.

TL16: **Dorsal glands in male cloaca not (0) bifurcated (1).** State 1 is limited to *Lyciasalamandra* and *Salamandra*, making the character parsimony-uninformative in our matrix.

TL30-WÖ21: **First hypobranchial does not (0) extend beyond caudal tip of second hypobranchial (1).** As TL stated on page 150, state 1 is restricted to *Chioglossa*, making the character parsimony-uninformative.

TL33-WÖ22: **Distinct lengthening loop of M. rectus cervicis profundus absent (0); present (1).** As TL stated on page 150, state 1 is restricted to *Salamandrina*, making the character parsimony-uninformative.

TL41-WÖ31: **Medial fibers of M. genioglossus inserting near tips of ceratohyals (0); medial fibers absent, lateral fibers well developed (1).** State 1 is limited to *Mertensiella*, *Lyciasalamandra* and *Salamandra*, making the character parsimony-uninformative in our matrix.

TL48-WÖ38: **M. interradialis well developed (0); not well developed (1); absent (2) (ordered).** We would thus have exchanged states 0 and 1 compared to our sources so as to be able to order this potentially continuous character.–Our state 0 is limited to *Salamandrina*, making this state uninformative. State 2 is limited to *Mertensiella*, *Lyciasalamandra* and *Salamandra*, making the character wholly parsimony-uninformative in our matrix.

YZ9: **Zygosphene and zygantrum absent (0); present (1).** Among salamandrids, state 1 occurs only in *Salamandrina* and in *Chelotriton pliocenicus*, which is not included in this matrix; the character is therefore uninformative.

AS13: **Creep present.** No other state occurs in the original sample, or probably in any salamanders with internal but not direct fertilization; the character is thus parsimony-uninformative.

AS14: **Tail-touching present.** No other state occurs in the original sample, or probably in any salamanders with internal but not direct fertilization; the character is thus parsimony-uninformative.

New character: **At least one spine on quadratojugal-quadrate bone: absent (0); present (1).** This would have been an obvious character to add: state 1 occurs in *Echinotriton* [8,45] and, as pointed out in [30], the Enspel and Randeck specimens of *Chelotriton*. Unfortunately, this character is inapplicable unless the quadratojugal-quadrate bone forms a substantial part of the ventrolateral skull margin, and it is only visible in ventral view in the Enspel and Randeck specimens of *Chelotriton* [30]. MB.Am.45 is preserved in dorsal view, as are apparently all specimens of *Brachycormus* where this bone is visible, so they would have to be scored as unknown; the same would apply to *Salamandra*, because *S*. “*laticeps*”–to which this character might be applicable–is likewise preserved in dorsal view. This means that state 0 could only be scored for *Tylototriton*, making the character parsimony-uninformative.

## Supporting Information

S1 Data Data matrix for analysis of salamandrid phylogeny in NEXUS format (plain text). Our analyses are coded in the PAUP block–executing the file repeats the constrained analysis with character 98 (geographic distribution) included, unless the PAUP block is modified or commented out first. To repeat an unconstrained analysis, replace “enforce = yes” by “enforce = no” in the line that begins with “hsearch”; to exclude character 98, add “exclude 98;” above that line.
(NEX)

S1 Fig Backside of MB.Am.45.1, showing the specimen number, the accession number and the locality. (TIF)

S2 Fig Plaster cast MB.Am.45.2. (TIF)

S3 Fig Detail of plaster cast MB.Am.45.2, showing the head. Photograph taken by holding a camera to an ocular of a binocular microscope. Note that what looks like a pterygoid in the left orbitotemporal fenestra is an artefact of abrasion, as shown by comparison to MB.Am.45.1 and MB.Am.45.3.
(TIF)

S4 Fig Strict consensus of the most parsimonious trees from our unconstrained analysis with character 98 included. This figure differs from Fig 16 in that no taxa were pruned.
(PDF)

S5 Fig Adams consensus of the most parsimonious trees from our unconstrained analysis with character 98 included. No taxa were pruned.
(PDF)

S6 Fig Adams consensus of the most parsimonious trees from our constrained analysis without character 98 (see Fig 17 for the strict consensus). No taxa were pruned.
(PDF)

S7 Fig Strict consensus of the most parsimonious trees from our constrained analysis with character 98 included. This figure differs from Fig 19 in that no taxa were pruned.
(PDF)

S8 Fig Adams consensus of the pruned most parsimonious trees from our constrained analysis with character 98 included. The same taxa were pruned as in Fig 19.
(PDF)

## References

[1] Zhang P, Papenfuss JP, Wake MH, Qu L, Wake DB. Phylogeny and biogeography of the family Salamandridae (Amphibia: Caudata) inferred from complete mitochondrial genomes. Mol Phyl Evol. 2008; 49:586–597. 10.1016/j.ympev.2008.08.020 18801447

[2] Pyron RA. Biogeographic analysis reveals ancient continental vicariance and recent oceanic dispersal in amphibians. Syst Biol. 2014; 63:779–797. 10.1093/sysbio/syu042 24951557

[3] Venczel M. A new salamandrid amphibian from the Middle [sic] Miocene of Hungary and its phylogenetic relationships. J Syst Palaeont. 2008; 6:41–59.

[4] Estes R. Gymnophiona, Caudata Part 2 of Wellnhofer P., editor. Handbuch der Paläoherpetologie | Encyclopedia of Paleoherpetology. Stuttgart and New York: Gustav Fischer; 1981. [English.]

[5] Milner AR. Mesozoic and Tertiary Caudata and Albanerpetontidae In: Heatwole H, Carroll RL, editors. Palaeontology. Volume 4 of Heatwole H, editor. Amphibian Biology. Chipping Norton: Surrey Beatty & Sons; 2000 pp. 1412–1444.

[6] Marjanović D, Laurin M. Fossils, molecules, divergence times, and the origin of lissamphibians. Syst Biol. 2007; 56:369–388. 17520502 10.1080/10635150701397635

[7] Marjanović D, Laurin M. An updated paleontological timetree of lissamphibians, with comments on the anatomy of Jurassic crown-group salamanders (Urodela). Hist Biol. 2013, paginated and printed 2014; 26:535–550.

[8] Nussbaum RA, Brodie ED Jr. Partitioning of the salamandrid genus *Tylototriton* Anderson (Amphibia: Caudata) with a description of a new genus. Herpetologica. 1982; 38:320–332.

[9] Buckley D, Sanchiz B. Untrained *versus* specialized palaeontological systematics: A phylogenetic validity test using morphostructural conspicuity as character weight. Span J Palaeont. 2012; 27:131–142.

[10] Herre W. *Palaeopleurodeles hauffi* nov. gen. nov. spec., ein fossiler Schwanzlurch aus dem Miocän Süddeutschlands. Zool Anz. 1941; 134:1–17. [German.]

[11] Westphal F. Miozäne Salamandriden aus dem Randecker Maar (Schwäbische Alb). Ber Naturf Ges Freiburg Breisgau. 1977; 67:393–403. [German with English abstract.]

[12] Roček Z. A review of the fossil Caudata of Europe. Abh Ber Naturkunde. 1994; 17:51–56.

[13] Roček Z. Heterochrony: response of Amphibia to cooling events. Geolines. 1995; 3:55–58.

[14] Roček Z. The salamander *Brachycormus noachicus* from the Oligocene of Europe, and the role of neoteny in the evolution of salamanders. Palaeontology. 1996; 39:477–495.

[15] Roček Z. Skull of the neotenic salamandrid amphibian *Triturus alpestris* and abbreviated development in the Tertiary Salamandridae. J Morphol. 1996; 230:187–197. 29852670 10.1002/(SICI)1097-4687(199611)230:2<187::AID-JMOR6>3.0.CO;2-E

[16] Böhme M. *Archaeotriton basalticus* (v. Meyer, 1859) (Urodela, Salamandridae) aus dem Unteroligozän von Hammerunterwiesenthal (Freistaat Sachsen). Abh Staatl Mus Mineralogie Geologie Dresden. 1998; 43/44:265–280. [German with English abstract.]

[17] Westphal F. *Chelotriton robustus* n. sp., ein Salamandride aus dem Eozän der Grube Messel bei Darmstadt. [*Chelotriton robustus* n. sp., a salamandrid from the Eocene of the Messel pit near Darmstadt.] Senckenb lethaea. 1980; 60:475–487. [German with English abstract.]

[18] Bailon S. Les amphibiens et les reptiles du Pliocène supérieur de Balaruc II (Hérault, France). Palaeovertebrata. 1989; 19:7–28. [French with English abstract.]

[19] Böhme M. Ectothermic vertebrates (Actinopterygii, Allocaudata, Urodela, Anura, Crocodylia, Squamata) from the Miocene of Sandelzhausen (Germany, Bavaria) and their implications for environment reconstruction and palaeoclimate. Paläont Z. 2010; 84:3–41.

[20] Pomel A. Catalogue méthodique et descriptif des vertébrés fossiles découverts dans le bassin hydrographique supérieur de la Loire Paris: J. B. Baillières; 1853. [French; not seen]

[21] von Meyer H. Salamandrinen aus der Braunkohle am Rhein und in Böhmen. Palaeontographica. 1860; VII:47–73 + plates VIII, IX fig 1. [German.]

[22] Noble GK. Two new fossil Amphibia of zoogeographic importance from the Miocene of Europe. Am Mus Novitates. 1928; 303:1–13.

[23] Herre W. Neue Tatsachen zur Stammesgeschichte der Schwanzlurche. Zool Jahrb (Abt Syst). 1949; 78:217–236. [German.]

[24] Westphal F. *Tylototriton* (Amphibia, Urodela) aus dem Obermiozän von Öhningen. [*Tylototriton* (Amphibia, Urodela) from the Late [sic] Miocene of Öhningen.] N Jb Geol Paläont, Monatsh. 1978; 1978:491–501. [German with English abstract.]

[25] Hellmund M, Böhme W. Zweiter Fund eines vollständigen Exemplares von *Chelotriton paradoxus* Pomel, 1853 aus dem Oberoligozän von Rott bei Bonn. Salamandra. 1987; 23:142–152. [German with very short English abstract.]

[26] Rage J-C, Hossini S. Les Amphibiens du Miocène moyen de Sansan. In: Ginsburg L, editor. La faune miocène de Sansan et son environnement. Mém Mus natl Hist nat. 2000; 183:177–217. [French with English abstract.]

[27] Miklas PM. Die Amphibienfauna (Amphibia: Caudata, Anura) der obermiozänen Fundstelle Götzendorf an der Leitha (südliches Wiener Becken, Niederösterreich). Ann Naturhist Mus Wien. 2002; 103A:161–211. [German with English abstract.]

[28] Venczel M, Ştiucă E. Late middle Miocene amphibians and squamate reptiles from Tauţ, Romania. Geodiversitas. 2008; 30:731–763.

[29] Roček Z, Wuttke M. Amphibia of Enspel (Late Oligocene, Germany). Palaeobio Palaeoenv. 2010; 90:321–340.

[30] Schoch RR, Poschmann M, Kupfer A. The salamandrid *Chelotriton paradoxus* from Enspel and Randeck Maar (Oligocene–Miocene, Germany). Palaeobio Palaeoenv. 2015; 95:77–86.

[31] Roček Z. Late Miocene Amphibia from Rudabánya. Palaeontographia Italica. 2005; 90:11–29.

[32] Ivanov M. Early Miocene amphibians (Caudata, Salientia) from the Mokrá-Western Quarry (Czech Republic) with comments on the evolution of Early Miocene amphibian assemblages in Central Europe. Geobios. 2008; 41:465–492.

[33] Sanchiz B. Vertebrates from the Early [sic] Miocene lignite deposits of the opencast mine Oberdorf (Western Styrian Basin, Austria): 2. Amphibia. Ann Naturhist Mus Wien. 1998; 99A:13–29.

[34] Herre W. Die Schwanzlurche der mitteleocänen (oberlutetischen) Braunkohle des Geiseltales und die Phylogenie der Urodelen unter Einschluß der fossilen Formen. Zoologica (Stuttgart). 1935; 33:1–85. [German.]

[35] Estes R, Hoffstetter R. Les Urodèles du Miocène de La Grive-Saint-Alban (Isère, France). Bull Mus natl Hist nat 3^e^ sér. 1976; 398 (Sci Terr 57):297–344. [French with English abstract.]

[36] Veith M, Degani G, Seitz A. Discordance of genetical and morphological variation of *Salamandra salamandra* (L.) in Israel. Zool Anz. 1992; 229:63–72. [English with German summary.]

[37] von Koenigswald W, Martin T, Mörs T, Pfretzschner HU. Die oberoligozäne Wirbeltierfauna von Rott bei Hennef am Siebengebirge–Synonymien und Literatur 1828–1991. Decheniana. 1992; 145:312–240. [German with English abstract.]

[38] Wang Y, Evans SE. A new short-bodied salamander from the Upper Jurassic/Lower Cretaceous of China. Acta Palaeont Pol. 2006; 51:127–130.

[39] Böhme M. Ectothermic vertebrates (Teleostei, Allocaudata, Urodela, Anura, Testudines, Choristodera, Crocodylia, Squamata) from the Upper [sic] Oligocene of Oberleichtersbach (Northern Bavaria, Germany). Cour Forsch-inst Senckenb. 2008; 260:161–183.

[40] Haller-Probst M, Schleich HH. Vergleichende osteologische Untersuchungen an einigen Urodelen Eurasiens (Amphibia: Urodela, Salamandridae, Proteidae). Cour Forsch-inst Senckenb. 1994; 173:23–77. [German with shortened English abstract.]

[41] Duellman WE, Trueb L. Biology of Amphibians New York: McGraw-Hill; 1986.

[42] Witzmann F, Scholz H, Müller J, Kardjilov N. Sculpture and vascularization of dermal bones, and the implications for the physiology of basal tetrapods. Zool J Linn Soc 2010; 160:302–340.

[43] Rose CS. The developmental morphology of salamander skulls In: Heatwole H, Davies M, editors. Osteology. Volume 5 of Heatwole H, editor. Amphibian Biology Chipping Norton: Surrey Beatty & Sons; 2003 pp. 1684–1781.

[44] Gao K-Q, Shubin NH. Late Jurassic salamandroid from western Liaoning, China. Proc Natl Acad Sci USA. 2012; 109:5767–5772. 10.1073/pnas.1009828109 22411790 PMC3326464

[45] Hou M, Wu Y, Yang K, Zheng S, Yuan Z, Li P. A missing geographic link in the distribution of the genus *Echinotriton* (Caudata: Salamandridae) with description of a new species from southern China. Zootaxa. 2014; 3895:89–102. 10.11646/zootaxa.3895.1.5 25543556

[46] Ivachnenko MF. Urodelans from the Triassic and Jurassic of Soviet Central Asia. Paleont J. 1978, translated 1979; 1978:362–368.

[47] Reilly SM, Lauder GV. Atavisms and the homology of hyobranchial elements in lower vertebrates. J Morphol. 1988; 195:237–245. 29874889 10.1002/jmor.1051950302

[48] Witzmann F. Phylogenetic patterns of character evolution in the hyobranchial apparatus of early tetrapods. Earth Env Sci Trans R Soc Edinb. 2013; 104:145–167.

[49] Wiedersheim R. Das Kopfskelet der Urodelen. [Gegenbaurs] Morphol Jb. 1877; 3:352–448 + plates XIX–XXIII and 459–548 + plates XXIV–XXVII. [German.]

[50] Özeti N, Wake DB. The morphology and evolution of the tongue and associated structures in salamanders and newts (Family Salamandridae). Copeia. 1969; 1969:91–123.

[51] Wake DB, Özeti N. Evolutionary relationships in the family Salamandridae. Copeia 1969; 1969:124–137.

[52] Titus TA, Larson A. A molecular phylogenetic perspective on the evolutionary radiation of the salamander family Salamandridae. Syst Biol. 1995; 44:125–151.

[53] Francis ETB. The Anatomy of the Salamander. Oxford: Clarendon; 1934.

[54] Maddison WP, Maddison DR. Mesquite: a modular system for evolutionary analysis [Software]. Version 2.75; 2011. Available: http://mesquiteproject.wikispaces.org.

[55] Swofford DL. PAUP*–Phylogenetic Analysis Using Parsimony (*and Other Methods) [Software]. Version 4.0a136; 2014. Distributed by the author to alpha-testers in cooperation with Sinauer Associates (Sunderland, Massachusetts).

[56] Wright AM, Hillis DM. Bayesian analysis using a simple likelihood model outperforms parsimony for estimation of phylogeny from discrete morphological data. PLOS ONE. 2014; 9:e109210 10.1371/journal.pone.0109210 25279853 PMC4184849

[57] Bolkay SJ [= IJ]. Die Schädel der Salamandrinen, mit besonderer Rücksicht auf ihre systematische Bedeutung. Z ges Anat, I. Abt. 1928; 86:259–319. [German.]

[58] Kalusa JW. Cranial and cervical myology and osteology of the adult California Newt (Taricha torosa) in relation to its feeding. M.Sc. thesis, California State University (Northridge). 1976. Available: http://scholarworks.csun.edu/bitstream/handle/10211.2/5438/KalusaJohn1976.pdf

[59] Wu Y, Wang Y, Hanken J. Comparative osteology of the genus *Pachytriton* (Caudata: Salamandridae) from southeastern China. Asian Herpetol Res. 2012; 3:83–102.

[60] Schoch RR, Rasser MW. A new salamandrid from the Miocene Randeck Maar, Germany. J Vert Paleont. 2013; 33:58–66.

[61] Arntzen JW, Sparreboom M. A phylogeny for the Old World newts, genus *Triturus*: biochemical and behavioural data. J Zool. 1989; 219:645–664.

[62] Houck LD, Arnold SJ. Courtship and mating behavior In: Sever DM, editor. Reproductive Biology and Phylogeny of Urodela. Enfield: Science Publishers; 2003 pp. 383–424.

[63] Giacoma C, Balletto E. Phylogeny of the salamandrid genus *Triturus* . Boll Zool. 1988; 55:337–360.

[64] Sever DM. Comparative anatomy and phylogeny of the cloacae of salamanders (Amphibia: Caudata).–I. Evolution at the family level. Herpetologica. 1991; 47:165–193.

[65] Sever DM. Comparative anatomy and phylogeny of the cloacae of salamanders (Amphibia: Caudata)–IV. Salamandridae. Anat Rec. 1992; 232:229–244. 10.1002/ar.1092330206 1605387

[66] Carranza S, Amat F. Taxonomy, biogeography and evolution of *Euproctus* (Amphibia: Salamandridae), with the resurrection of the genus *Calotriton* and the description of a new endemic species from the Iberian peninsula. Zool J Linn Soc. 2005; 145:555–582.

[67] DigiMorph Staff. *Taricha torosa*, California Newt. Digital Morphology; 2008. Available: http://www.digimorph.org/specimens/Taricha_torosa/head/ and http://www.digimorph.org/specimens/Taricha_torosa/whole/. Both accessed 18 December 2013–8 September 2014.

[68] AmphibiaTree. *Ambystoma gracile*, Northwestern Salamander. Digital Morphology; 2008. Available: http://www.digimorph.org/specimens/Ambystoma_gracile/head/ and http://www.digimorph.org/specimens/Ambystoma_gracile/whole/. Accessed 16 December 2013–8 September 2014.

[69] DigiMorph Staff. *Ambystoma tigrinum*, Tiger Salamander. Digital Morphology; 2008. Available: http://www.digimorph.org/specimens/Ambystoma_tigrinum/head/ and http://www.digimorph.org/specimens/Ambystoma_tigrinum/whole/. Accessed 16 December 2013–8 September 2014.

[70] Gu X, Chen R, Tian Y, Li S, Ran J. A new species of *Paramesotriton* (Caudata: Salamandridae) from Guizhou Province, China. Zootaxa. 2012; 3510:41–52.

[71] AmphibiaTree, Gosselin-Ildari A. *Pleurodeles waltl*, Spanish Ribbed Newt. Digital Morphology; 2007. Available: http://www.digimorph.org/specimens/Pleurodeles_waltl/head/ and http://www.digimorph.org/specimens/Pleurodeles_waltl/whole/. Both accessed 18 December 2013–8 September 2014.

[72] Yu P, Zhao E. Research of the skeleton system of *Tylototriton kweichowensis* . Sichuan J Zool. 2007; 26:133–140.

[73] Whitmore SS, Losee S, Meyer L, Spradling TA. Conservation genetics of the central newt (*Notophthalmus viridescens*) in Iowa: the importance of a biogeographic framework. Conserv Genet. 2013; 14:771–781.

[74] Akia F, Rastegar-Pouyani R, Faizi H. The comparison of cranial osteology of *Neurergus microspilotus* and *Salamandra infraimmaculata semenovi* (Amphibia: Salamandridae). Russian J Herpetol. 2010; 17:179–184.

[75] Wu Y, Jiang K, Hanken J. A new species of newt of the genus *Paramesotriton* (Salamandridae) from southwestern Guangdong, China, with a new northern record of *P*. *longliensis* from western Hubei. Zootaxa. 2010; 2494:45–58.

[76] Wu Y, Wang Y, Jiang K, Hanken J. Significance of pre-Quaternary climate change for montane species diversity: Insights from Asian salamanders (Salamandridae: *Pachytriton*). Mol Phyl Evol. 2013; 66:380–390. 10.1016/j.ympev.2012.10.011 23110935

[77] Hilton WA. Preliminary remarks on the skeletons of Salamandridae. J Entom Zool. 1947; 39:37–43. [not seen]

[78] Ratnikov VYu, Litvinchuk SN. Comparative morphology of trunk and sacral vertebrae of tailed amphibians of Russia and adjacent countries. Russian J Herpetol. 2007; 14:177–190.

[79] Klembara J, Bartík I. The postcranial skeleton of *Discosauriscus* Kuhn, a seymouriamorph tetrapod from the Lower Permian of the Boskovice Furrow (Czech Republic). Trans R Soc Edinb: Earth Sci. 2000; 90:287–316.

[80] Desnitskiy AG, Litvinchuk SN. Comparative and phylogenetic perspectives of the cleavage process in tailed amphibians. Zygote. 2014 10.1017/S0967199414000379 25180466

[81] Halliday TR. The evolution of courtship behavior in newts and salamanders In: Slater PJB, Milinski M, editors. Advances in the Study of Behavior. Volume 19 San Diego: Academic Press; 1990 pp. 137–169.

[82] Sparreboom M, Steinfartz S, Schultschik G. Courtship behavior of *Neurergus* (Caudata: Salamandridae). Amphibia-Reptilia. 2000; 21:1–11.

[83] Ratnikov VYu, Litvinchuk SN. Atlantal vertebrae of tailed amphibians of Russia and adjacent countries. Russian J Herpetol. 2009; 16:57–68.

[84] AmphibiaWeb: information on amphibian biology and conservation. Available: http://amphibiaweb.org/. Accessed 10 September 2014.

[85] Frost DR. Amphibian Species of the World: an Online Reference. Version 6.0. Available: http://research.amnh.org/vz/herpetology/amphibia/. Accessed 10 September 2014 and 17 December 2014.

[86] Venczel M, Hír J. Amphibians and squamates from the Miocene of Felsőtárkány Basin, N-Hungary. Palaeontographica Abt A: Palaeozool–Stratigr. 2013; 300:117–158.

[87] Martín C, Alonzo-Zaragaza MA, Sanchiz B. Nomenclatural notes on living and fossil amphibians. Graellsia. 2012; 68:159–180.

[88] Jacisin J, Hopkins S. *Taricha* or *Palaeotaricha*? The evolutionary enigma of North American newts. J Vert Paleont. 2014; Program and Abstracts 2014:153.

[89] González-Fernández JE. Morfología comparada y relaciones filogenéticas de *Lissotriton boscai* (Caudata: Salamandridae). González-Fernández JE, editor. Madrid: Fuenlabrada; 2012. [Edited version of the author’s *licenciatura* thesis presented in 1985 or 1986 at the Universidad Complutense, Madrid.] Available: http://digital.csic.es/handle/10261/63066. [Spanish with English, French and German foreword.]

[90] Ivanović A, Arntzen JW. Evolution of skull and body shape in *Triturus* newts reconstructed from three-dimensional morphometric data and phylogeny. Biol J Linn Soc. 2014; 113:243–255.

[91] Ivanović A, Džukić G, Kalezić M. A phenotypic point of view of the adaptive radiation of crested newts (*Triturus cristatus* superspecies, Caudata, Amphibia). Intl J Evol Biol. 2012; 2012:740605. 10.1155/2012/740605 PMC3270399 22315697

[92] Marjanović D, Laurin M. A reevaluation of the evidence supporting an unorthodox hypothesis on the origin of extant amphibians. Contr Zool. 2008; 77:149–199.

[93] Young C-c [= Yang Z]. On the first occurrence of the fossil salamanders from the upper Miocene of Shantung, China. Acta Palaeont Sin. 1965; 13:455–462. [Chinese with extensive English summary.]

[94] Skutschas PP, Gubin YuM. A new salamander from the late Paleocene–early Eocene of Ukraine. Acta Palaeont Pol. 2012; 57:135–148.

[95] Bolt JR. Dissorophoid relationships and ontogeny, and the origin of the Lissamphibia. J Paleont. 1977; 51:235–249.

[96] Bolt JR. Lissamphibian origins In: Schultze H-P, Trueb L, editors. Origins of the Higher Groups of Tetrapods—Controversy and Consensus. Ithaca: Cornell University; 1991 pp. 194–222.

[97] Pawley K. The postcranial skeleton of temnospondyls (Tetrapoda: Temnospondyli). Ph.D. thesis, LaTrobe University (Melbourne). 2006. Available: http://arrow.latrobe.edu.au:8080/vital/access/manager/Repository/latrobe:19649

[98] Anderson JS. The origin(s) of modern amphibians. Evol Biol. 2008; 35:231–247.

[99] Smirnov SV. Extra bones in the Pelobates skull as evidence of the paedomorphic origin in the anurans. Zhurnal obshchey biologii. 1995; 56:315–328. [English with Russian abstract; lack of italics in the original.]

[100] Roček Z, Rage J-C. Anatomical transformations in the transition from temnospondyl to proanuran stages In Heatwole H, Carroll RL, editors. Palaeontology. Volume 4 of Heatwole H, editor. Amphibian Biology Chipping Norton: Surrey Beatty & Sons; 2000 pp. 1274–1282.

[101] Reilly SM. Ontogeny of cranial ossification in the Eastern newt, *Notophthalmus viridescens* (Caudata: Salamandridae), and its relationship to metamorphosis and neoteny. J Morphol. 1986; 188:315–326. 3735436 10.1002/jmor.1051880306

[102] Reilly SM, Lauder GV. Metamorphosis of cranial design in tiger salamanders (*Ambystoma tigrinum*): a morphometric analysis of ontogenetic change. J Morphol. 1990; 204:121–137. 29865725 10.1002/jmor.1052040202

[103] Reilly SM, Altig R. Cranial ontogeny in *Siren intermedia* (Caudata: Sirenidae): paedomorphic, metamorphic, and novel patterns of heterochrony. Copeia. 1996; 1996:29–41.

[104] Wake D. *Dicamptodon ensatus*, Pacific Giant Salamander. Digital Morphology; 2001. Available: http://www.digimorph.org/specimens/Dicamptodon_ensatus. Accessed 16 December 2013–8 September 2014.

[105] Witzmann F, Pfretzschner HU. Larval ontogeny of *Micromelerpeton credneri* (Temnospondyli, Dissorophoidea). J Vert Paleont. 2003; 23:750–76.

[106] Witzmann F, Scholz H. Morphometric study of allometric skull growth in the temnospondyl *Archegosaurus decheni* from the Permian/Carboniferous of Germany. Géobios. 2007; 40:541–554.

[107] Klembara J, Berman DS, Henrici AC, Čerňanský A, Werneburg R, Martens T. First description of skull of Lower Permian *Seymouria sanjuanensis* (Seymouriamorpha: Seymouriidae) at an early juvenile growth stage. Ann Carnegie Mus. 2007; 76:53–72.

[108] Vasilyan D, Böhme M. Pronounced peramorphosis in lissamphibians—*Aviturus exsecratus* (Urodela, Cryptobranchidae) from the Paleocene–Eocene Thermal Maximum of Mongolia. PLOS ONE. 2012; 7:e40665 10.1371/journal.pone.0040665 23028420 PMC3446925

[109] Panchen AL. The homologies of the labyrinthodont centrum. Evolution. 1967; 21:24–33. 28556114 10.1111/j.1558-5646.1967.tb00127.x

[110] Wiens JJ, Bonett RM, Chippindale PT. Ontogeny discombobulates phylogeny: paedomorphosis and higher-level salamander relationships. Syst Biol. 2005; 54:91–110. 15805013 10.1080/10635150590906037

