# Supplementary figures and images for: An Extremely Peramorphic Newt (Urodela: Salamandridae: Pleurodelini) from the Latest Oligocene of Germany, and a New Phylogenetic Analysis of Extant and Extinct Salamandrids
Source: PLoS One. 2015 Sep 30;10(9):e0137068. doi: 10.1371/journal.pone.0137068 (PMC4589347; doi:10.1371/journal.pone.0137068)

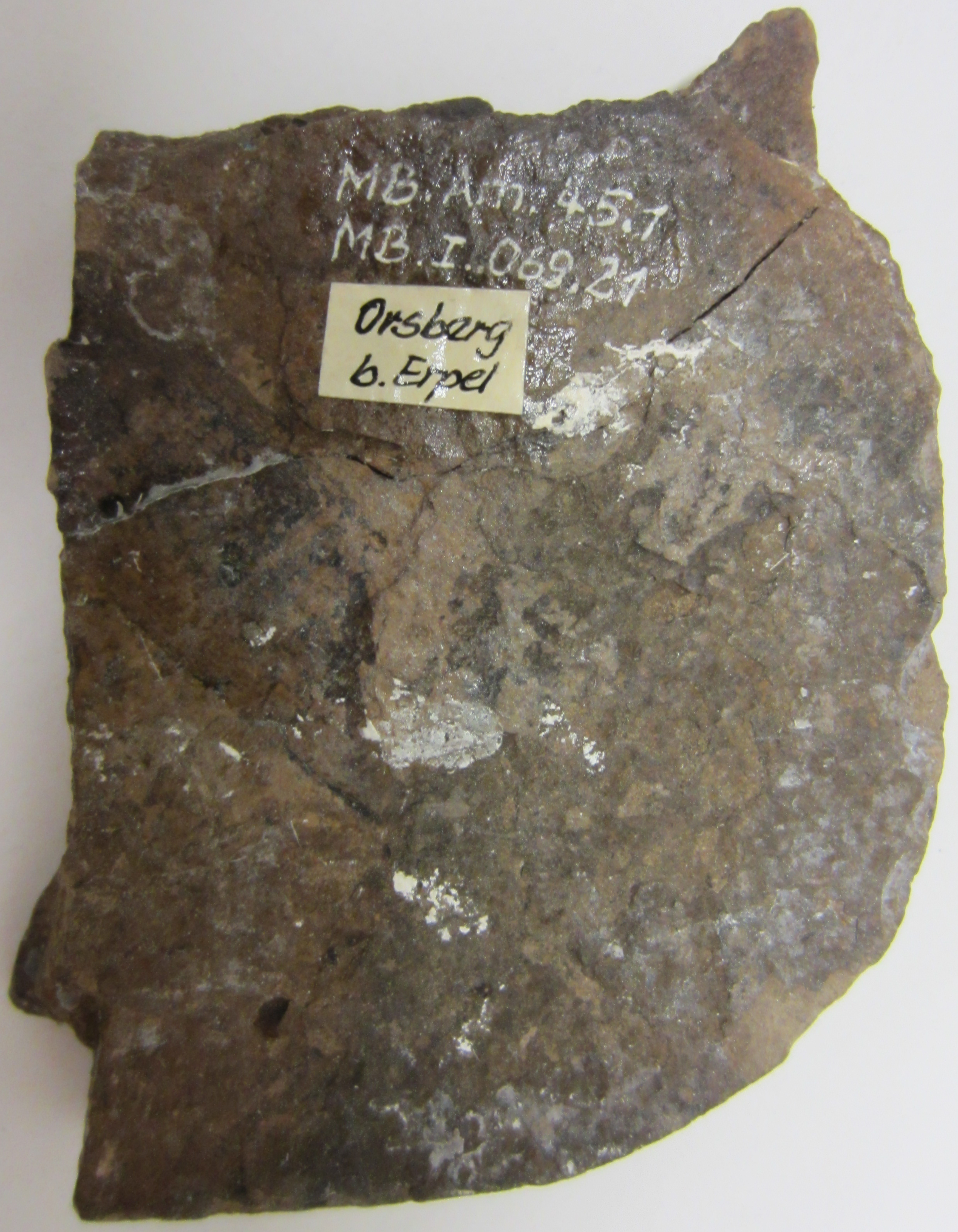

Supplement: S1 Fig — (TIF) [file pone.0137068.s002.tif]

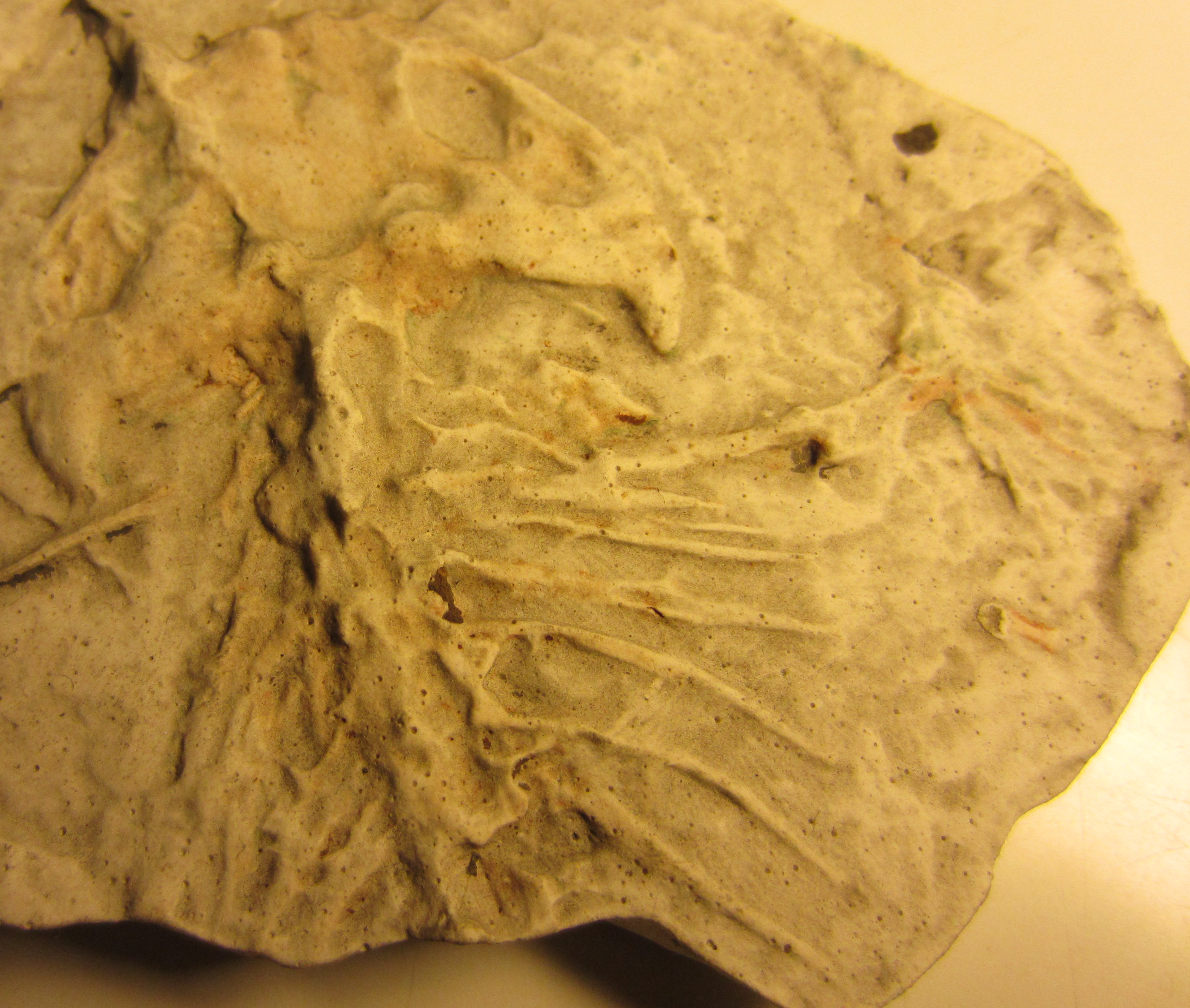

Supplement: S2 Fig — (TIF) [file pone.0137068.s003.tif]

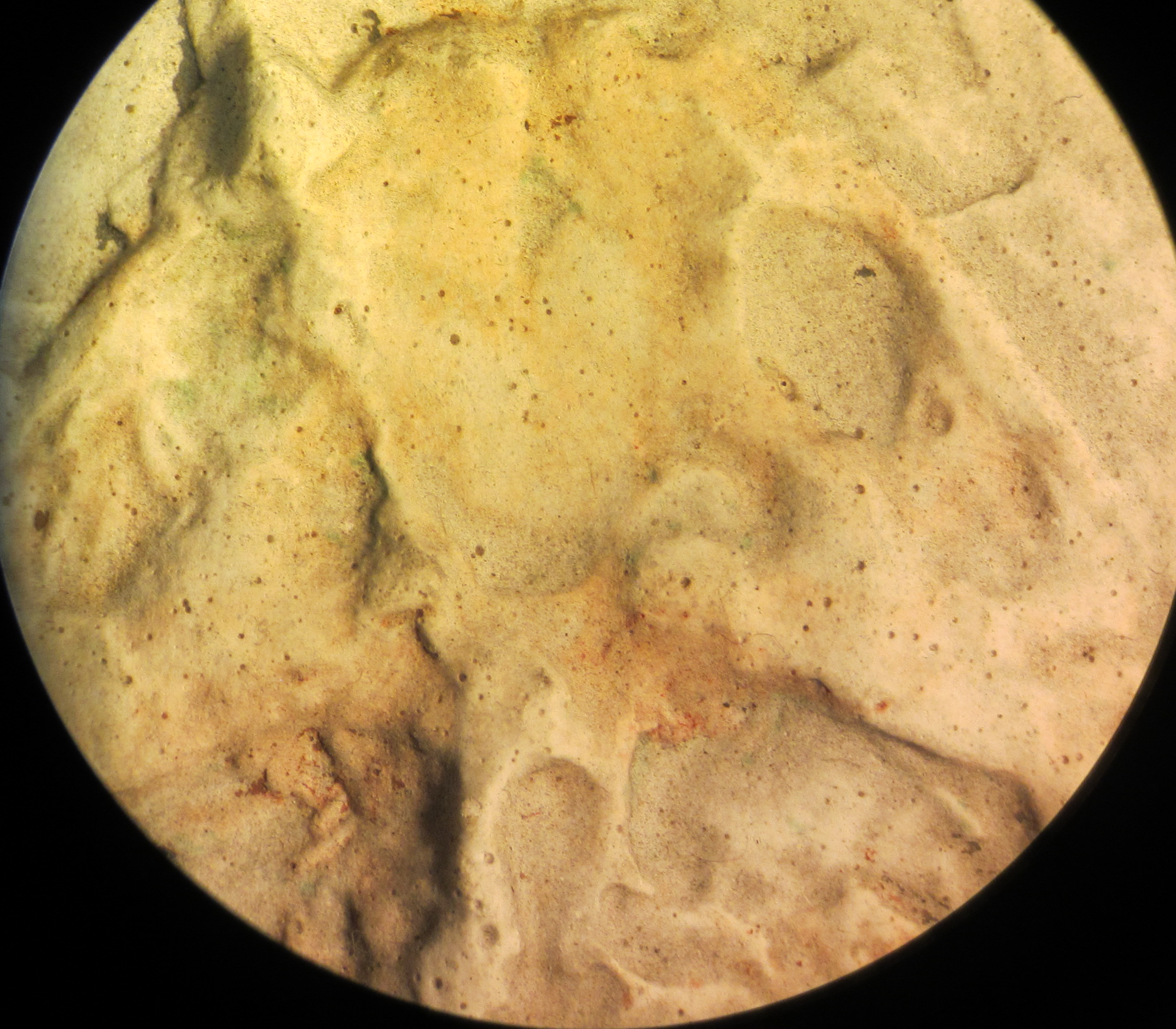

Supplement: S3 Fig — Photograph taken by holding a camera to an ocular of a binocular microscope. Note that what looks like a pterygoid in the left orbitotemporal fenestra is an artefact of abrasion, as shown by comparison to MB.Am.45.1 and MB.Am.45.3. (TIF) [file pone.0137068.s004.tif]

Strict consensus tree

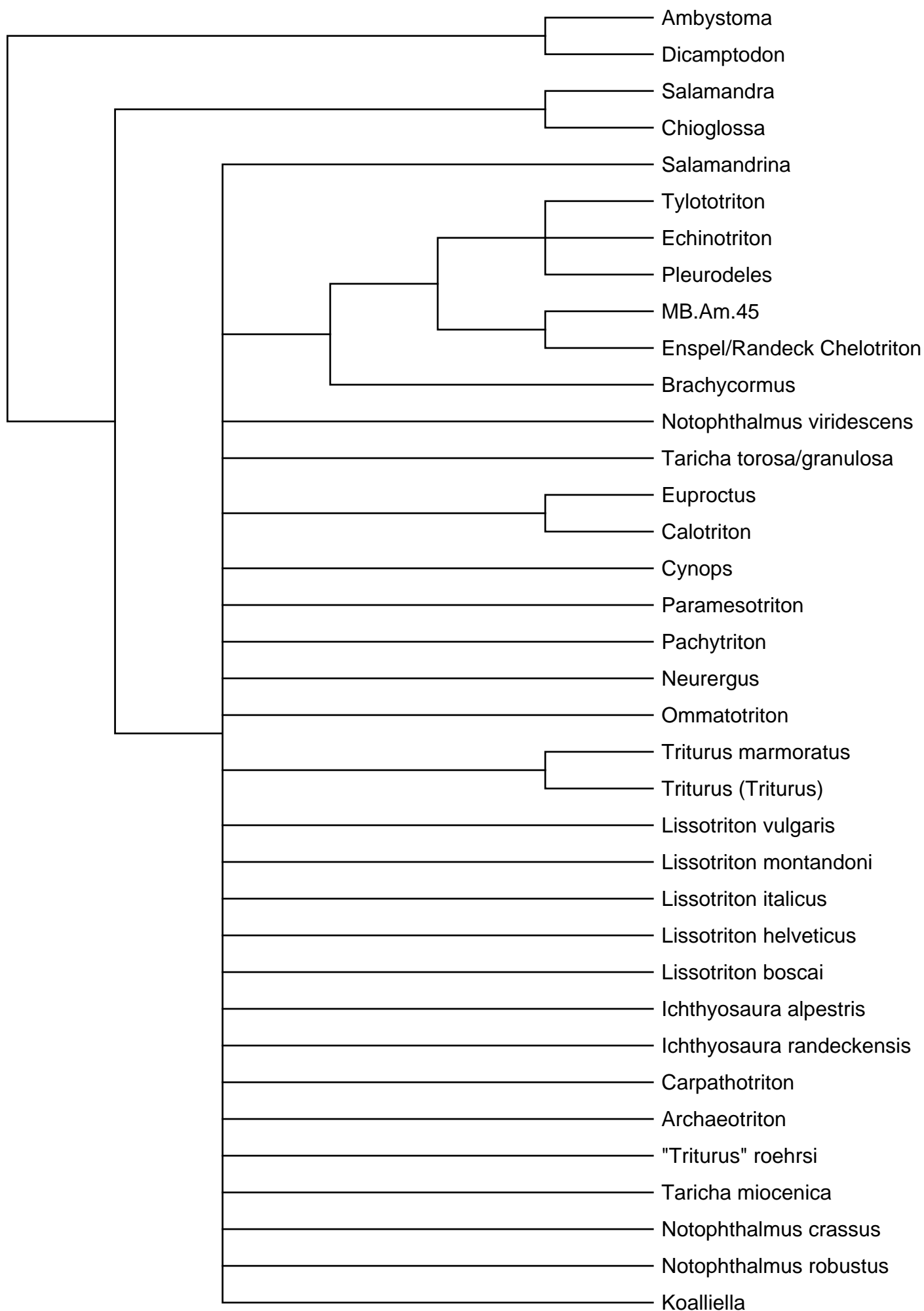

Supplement: S4 Fig — This figure differs from Fig 16 in that no taxa were pruned. (PDF) [file pone.0137068.s005.pdf]

Adams consensus tree

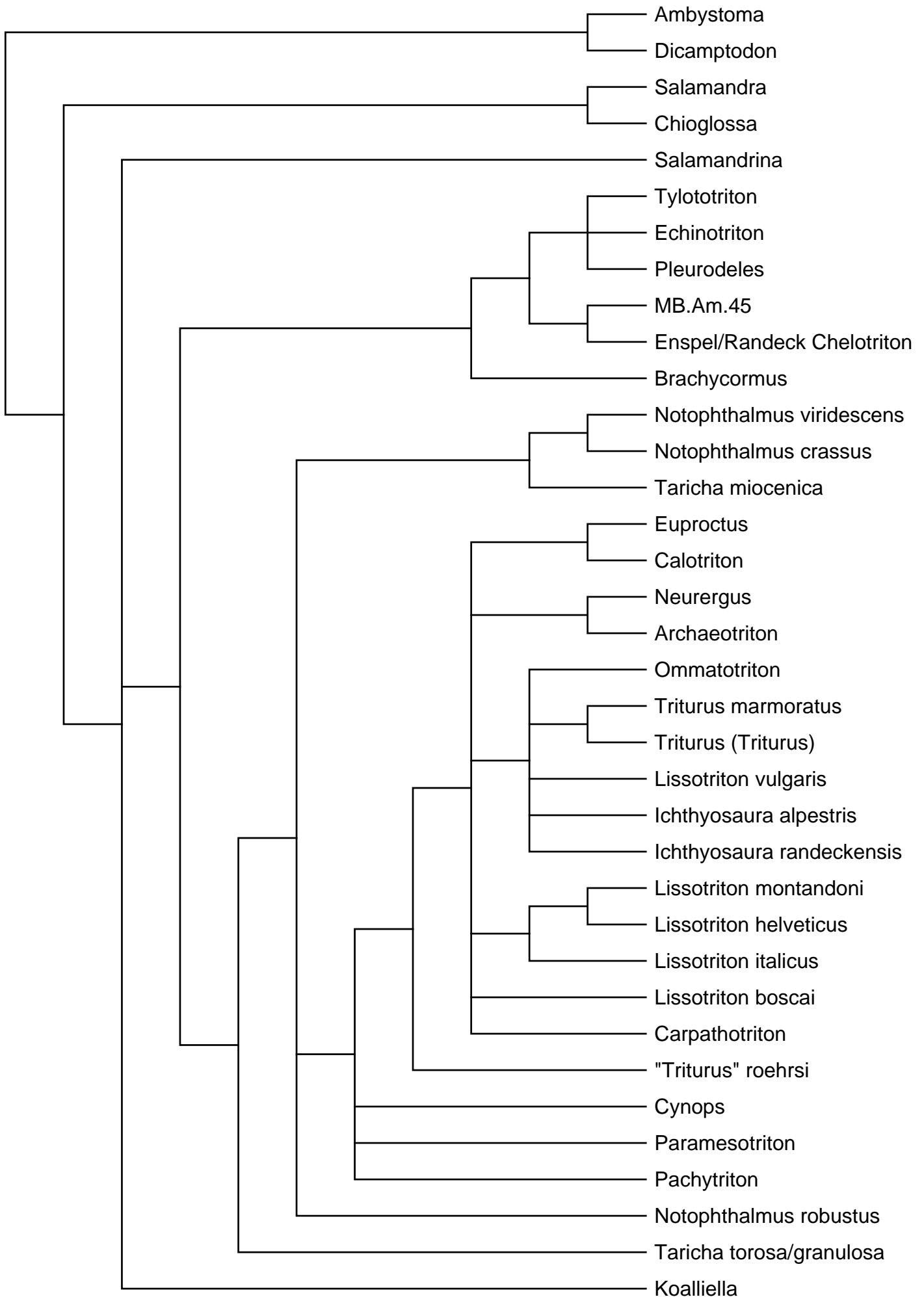

Supplement: S5 Fig — No taxa were pruned. (PDF) [file pone.0137068.s006.pdf]

Adams consensus tree

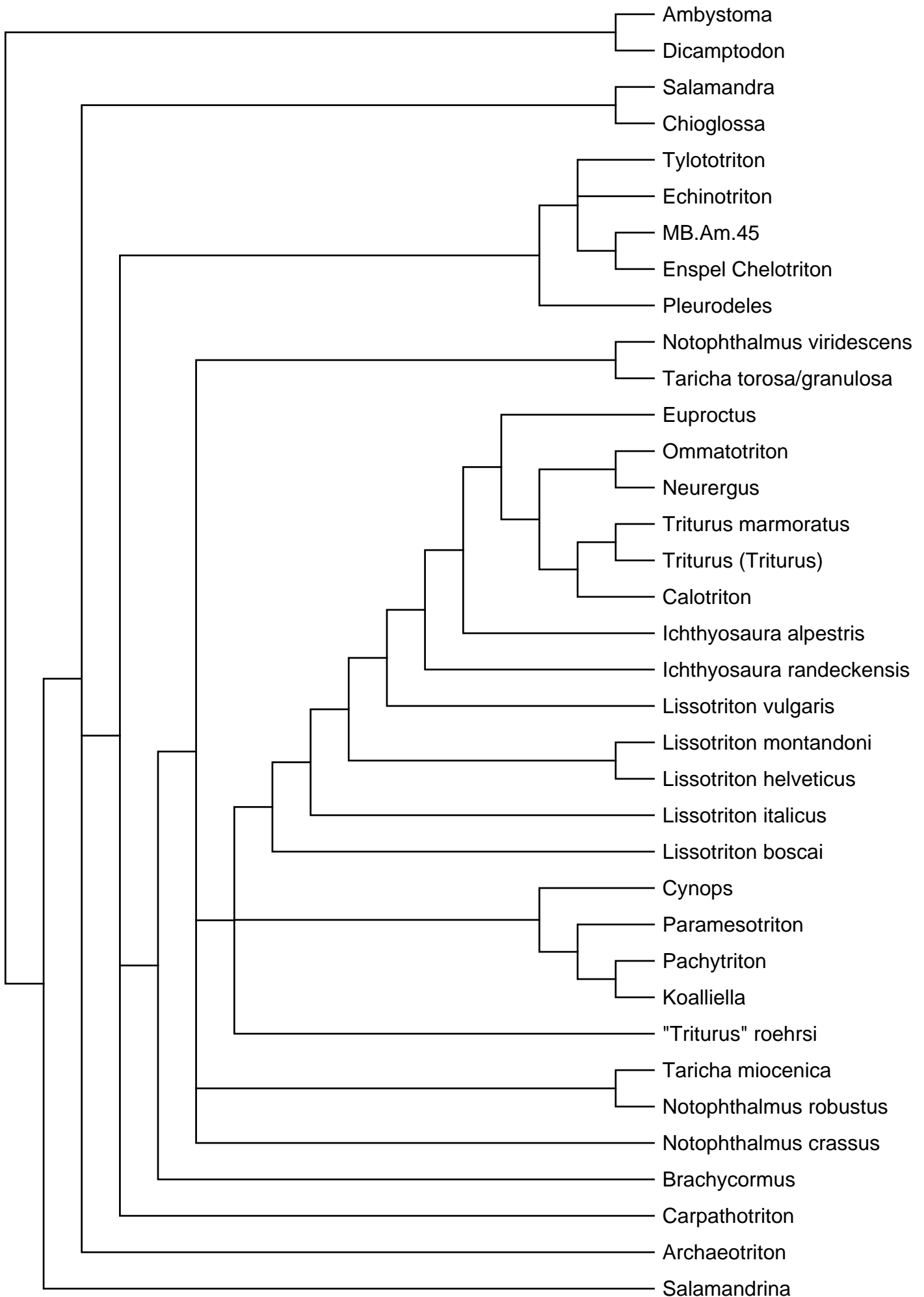

Supplement: S6 Fig — No taxa were pruned. (PDF) [file pone.0137068.s007.pdf]

Strict consensus tree

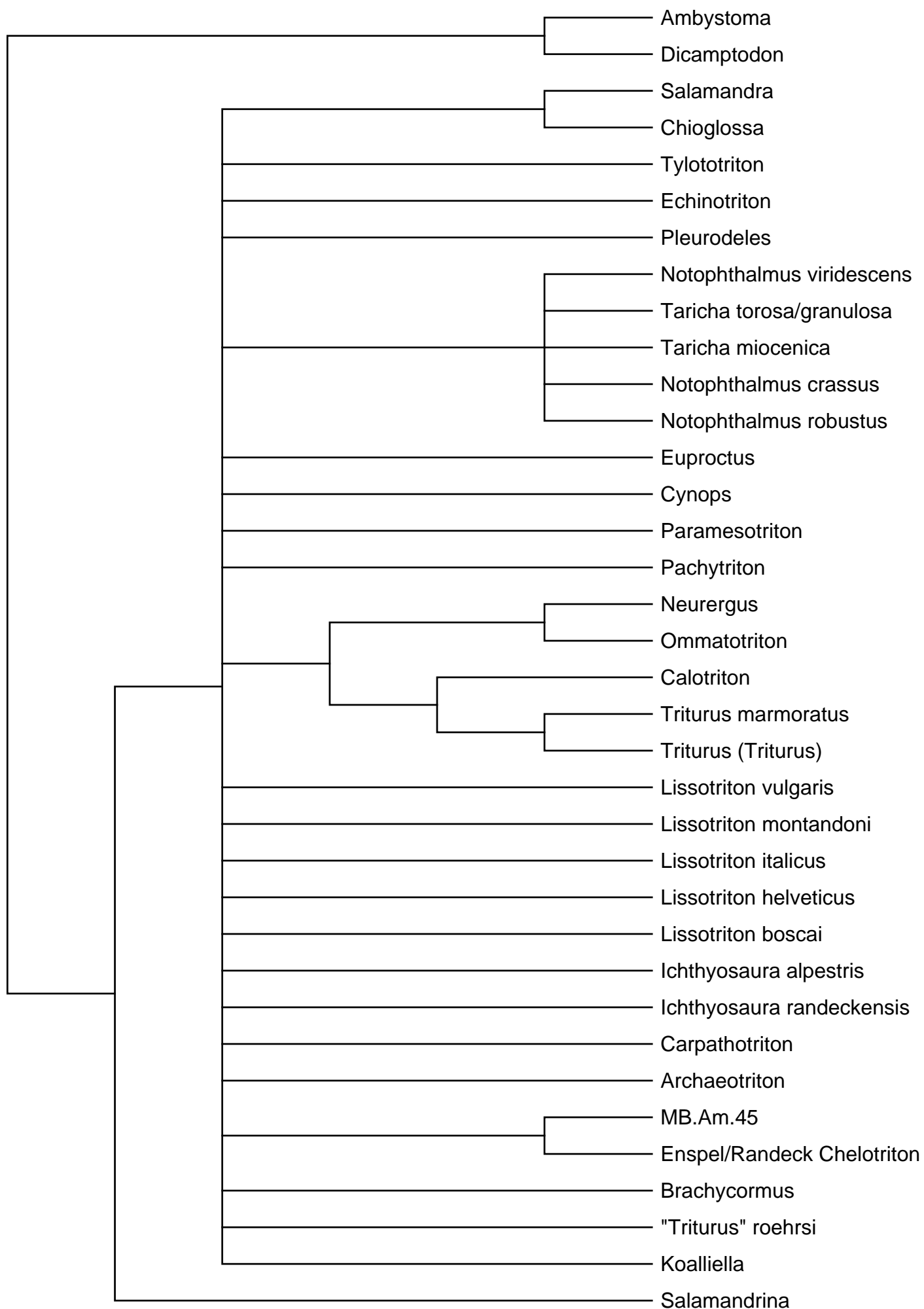

Supplement: S7 Fig — This figure differs from Fig 19 in that no taxa were pruned. (PDF) [file pone.0137068.s008.pdf]

Adams consensus tree

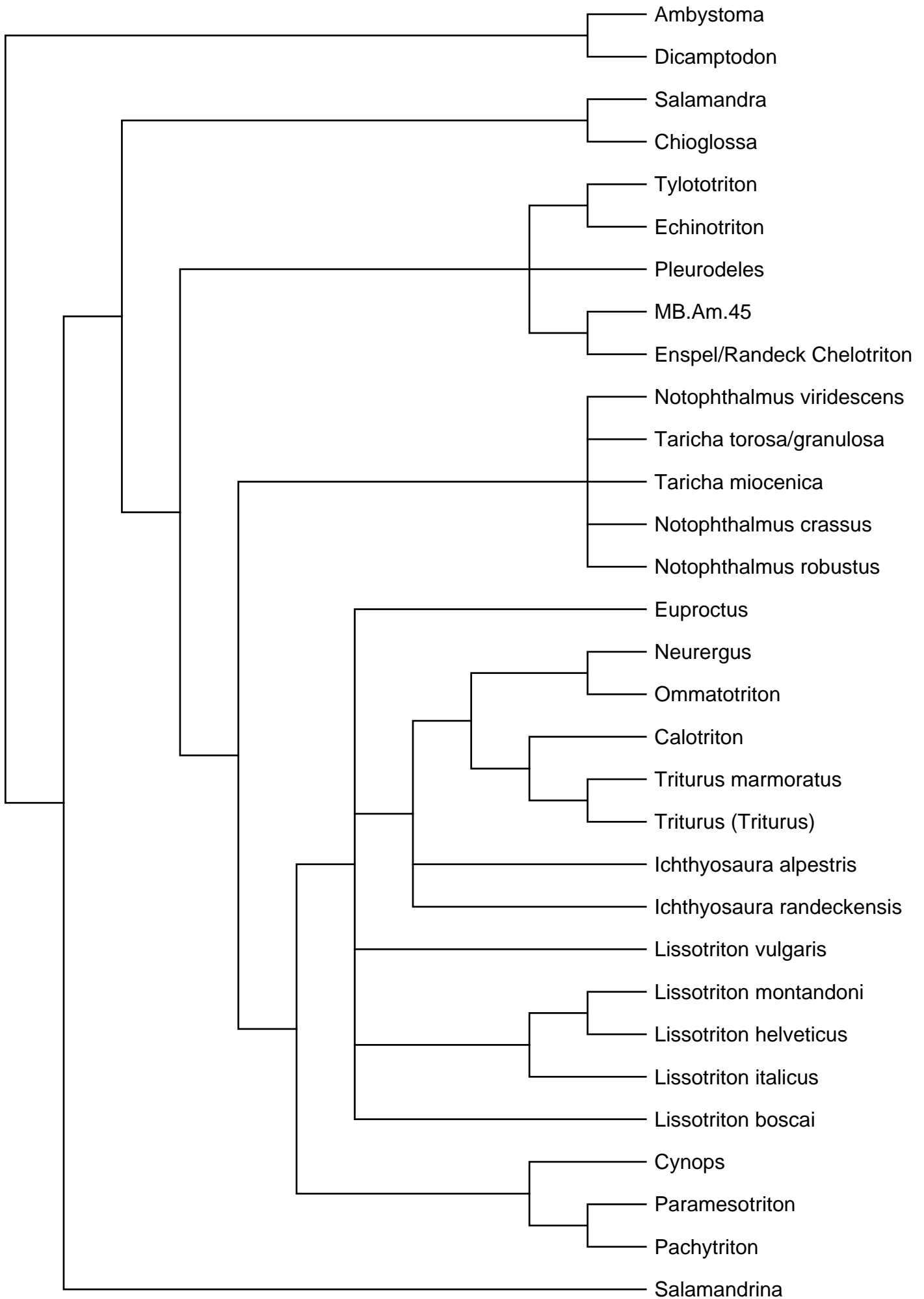

Supplement: S8 Fig — The same taxa were pruned as in Fig 19. (PDF) [file pone.0137068.s009.pdf]
